# Optimization of OPM-MEG Layouts with a Limited Number of Sensors

**DOI:** 10.3390/s25092706

**Published:** 2025-04-24

**Authors:** Urban Marhl, Rok Hren, Tilmann Sander, Vojko Jazbinšek

**Affiliations:** 1 Institute of Mathematics, Physics and Mechanics, SI-1000 Ljubljana, Slovenia; urban.marhl@imfm.si (U.M.); vojko.jazbinsek@imfm.si (V.J.); 2 Faculty of Mathematics and Physics, University of Ljubljana, SI-1000 Ljubljana, Slovenia; 3 Syreon Research Institute, 1142 Budapest, Hungary; 4 Physikalisch-Technische Bundesanstalt, 10587 Berlin, Germany; tilmann.sander-thoemmes@ptb.de

**Keywords:** magnetoencephalography, optically pumped magnetometers, sensor optimization, sequential selection algorithm, auditory-evoked fields, minimum norm estimation, magnetic field maps

## Abstract

Magnetoencephalography (MEG) is a non-invasive neuroimaging technique that measures weak magnetic fields generated by neural electrical activity in the brain. Traditional MEG systems use superconducting quantum interference device (SQUID) sensors, which require cryogenic cooling and employ a dense array of sensors to capture magnetic field maps (MFMs) around the head. Recent advancements have introduced optically pumped magnetometers (OPMs) as a promising alternative. Unlike SQUIDs, OPMs do not require cooling and can be placed closer to regions of interest (ROIs). This study aims to optimize the layout of OPM-MEG sensors, maximizing information capture with a limited number of sensors. We applied a sequential selection algorithm (SSA), originally developed for body surface potential mapping in electrocardiography, which requires a large database of full-head MFMs. While modern OPM-MEG systems offer full-head coverage, expected future clinical use will benefit from simplified procedures, where handling a lower number of sensors is easier and more efficient. To explore this, we converted full-head SQUID-MEG measurements of auditory-evoked fields (AEFs) into OPM-MEG layouts with 80 sensor sites. System conversion was done by calculating a current distribution on the brain surface using minimum norm estimation (MNE). We evaluated the SSA’s performance under different protocols, for example, using measurements of single or combined OPM components. We assessed the quality of estimated MFMs using metrics, such as the correlation coefficient (CC), root-mean-square error, and relative error. Additionally, we performed source localization for the highest auditory response (M100) by fitting equivalent current dipoles. Our results show that the first 15 to 20 optimally selected sensors (CC > 0.95, localization error < 1 mm) capture most of the information contained in full-head MFMs. Our main finding is that for event-related fields, such as AEFs, which primarily originate from focal sources, a significantly smaller number of sensors than currently used in conventional MEG systems is sufficient to extract relevant information.

## 1. Introduction

Noninvasive recording of brain activity is a rapidly expanding field. Its primary purpose is to improve brain pathology diagnostics and the study of brain physiology. One of the neuroimaging methods is magnetoencephalography (MEG) [1]. MEG measures weak magnetic fields around the head generated by neural activity. MEG traditionally employs superconducting quantum interference devices (SQUID) to measure magnetic fields [2]. Despite their unparalleled signal-to-noise ratio (SNR), these sensors have several drawbacks. They rely on superconductivity, which requires cooling with liquid helium to operate in a superconducting regime. This results in high maintenance costs [3]. Additionally, these sensors must be inside a bulky dewar, preventing them from being positioned close to the subject’s head. During measurements with SQUID-MEG, head movement must be minimized, which can be uncomfortable, especially for longer recordings.

In the last decade, optically pumped magnetometers (OPM) have become commercially available and used for MEG [4,5]. These sensors do not require cooling; therefore, we can place them closer to the scalp, which increases SNR [6,7,8]. Commercial OPMs often have a small footprint (<2 cm^2^) and can be placed arbitrarily on the head [9]. MEG systems with OPM sensors are still under development [10,11], and commercial complete OPM-MEG systems are rare. Most research groups use individual sensors with different approaches to place them on the subject’s head. Among the most established is the method of 3D printing a custom sensor holder for each subject based on the subject’s outer head surface determined from an MRI [6,12].

Over the last few years, OPM-MEG systems have proven valuable in clinical research [13]. Since OPMs, compared to SQUIDs, are not inside a bulky dewar, they allow for measurement where subject movement is required [14]. This proves extremely useful when measuring children who have difficulty keeping still for long periods. They have shown that OPM-MEG can be used to diagnose epileptic patients [15,16]. Moreover, MEG recordings of brain responses to auditory stimuli—specifically the M50, M100, and M200 components occurring approximately 50 ms, 100 ms, and 200 ms after stimulus onset—serve as valuable biomarkers for investigating neural processing in children with autism spectrum disorder (ASD) [17,18,19,20,21,22]. In this population, delays or atypical latencies, most notably in the M100 component, have been consistently observed, suggesting altered auditory cortical maturation or connectivity in ASD.

The current market price for commercial OPM sensors is several thousand euros per sensor. Like commercial SQUID-MEG systems, if we want to cover the entire scalp area, we need at least 50 sensors, which requires a significant financial investment. In this study, we explore if we can optimize a layout with a low sensor count to perform almost as well as a full head coverage layout. There are methods to optimize the placement of sensors to maximize the information measured [23]. Our work explores the feasibility of the sequential selection algorithm (SSA) for calculating an optimal layout for OPM-MEG. This method was initially developed for multi-channel electrocardiographic (ECG) systems [24]. SSA determines which channels contribute unique information from a database of multi-channel measurements. Previous studies have shown that this algorithm can be applied to MCG and SQUID-MEG [25,26,27,28]. This method requires a large database of MFMs. Currently, we do not have an OPM-MEG system that covers the whole head for obtaining a large database. We have, therefore, transformed the whole head SQUID-MEG measurements of auditory evoked fields (AEF) to OPM-MEG measurement sites. We used the best-performing transformation technique from a recent study [12]. This technique is based on the minimum norm estimate (MNE).

## 2. Materials and Methods

### 2.1. Measurements of Auditory Evoked Fields (AEFs)

In this study, we measured the AEFs using the SQUID-MEG system. The measurement setup was very similar to the one presented in [12] and was the following: the subject was lying inside the magnetically shielded room (MSR) on a bed, and its head was inside the SQUID-MEG system. The MSR consists of two layers of mu-metal and is located at the Physikalisch-Technische Bundesanstalt (PTB) in Berlin (AK3b, VAC, Hanau, Germany). The SQUID-MEG system is a prototype produced by KIT (Kanazawa Institute of Technology, later marketed by the company Yokogawa, Musashino, Japan) and employs 125 first-order gradiometers covering most of the subject’s scalp [29].

We obtained a magnetic resonance image (MRI) of the head for each subject using a clinical 3-Tesla scanner (Verio, Siemens Healthcare, Munich, Germany). The MRI images were later segmented with the FreeSurfer software (version 7.2) [30] to obtain individual layers of, e.g., the brain, skull, and scalp. These surfaces were used for co-registration and for advanced source reconstruction. Co-registration was based on five marker coils, which were attached to the subject’s head and localized inside the MEG system. The geometrical information of coils in relation to the subject’s head was determined using the CMS-HS motion analysis system made by the company Zebris Medical GmbH (Isny im Allgäu, Germany).

The tones for auditory stimulation were presented through non-magnetic earphones. The tones were generated outside the MSR by the Etymotic ER-30 speakers (www.etymotic.com). They were connected to two silicone tubes that were routed inside the MSR to the subject’s ears. The other ends of the silicon tubes were connected to 3D-printed earphones with added foam padding, which were placed inside the subject’s ears. The tone generator was connected to the data acquisition unit to mark individual times of auditory stimulation. We used the following protocol to obtain the average AEFs: the subject was presented with 500 auditory stimuli, each consisting of a 1 kHz tone with a duration of 500 ms. The interstimulus interval was 1.2 s.

The raw SQUID-MEG data was preprocessed. First, we visually inspected the data to identify and exclude bad channels. The raw time series were band-pass filtered (4–40 Hz) using a finite impulse response (FIR) filter implemented in MNE-Python (version 0.24.0) [31]. The average evoked response was computed using the events of the trigger channel linked to auditory stimulation.

We measured AEFs with the SQUID-MEG system on 9 healthy normal subjects; in 7 participants, the measurements were performed twice; the age group was from 26 to 58 years (Table 1). Non-removable metallic objects outside or inside the body were an exclusion criterion except for standard dental work with non-magnetic materials.

### 2.2. Transforming SQUID-MEG Measurements to the OPM-MEG System

One of the significant applications of the SSA algorithm is to optimize layouts of modular OPM-MEG systems where the sensor geometry is not predetermined. Since we do not have enough OPM sensors to cover the whole head and thus create whole-head OPM-MEG measurements, we transformed the whole-head SQUID-MEG measurements into a hypothetical OPM-MEG system with a chosen sensor geometry. A recent study [12] showed that this transformation between SQUID-MEG and OPM-MEG systems is feasible. The main concept is to perform source reconstruction with one system, which involves solving the inverse problem to determine the origin of the measured magnetic field maps (MFM) on a specific time interval. Once the inverse solution (source-time course) is obtained for one system, we compute the corresponding magnetic fields in the other system. We used the MNE algorithm and BEM method available in the MNE-Python (version 0.24.0) software for source reconstruction and forward modeling [31,32]. Theoretical background of MEG forward modeling of sources inside a multi-layer shell model and MNE method are in Appendix A.1 and Appendix A.2. Detailed information on the system transformation method is included in Appendix A.3.

**Figure 1:**
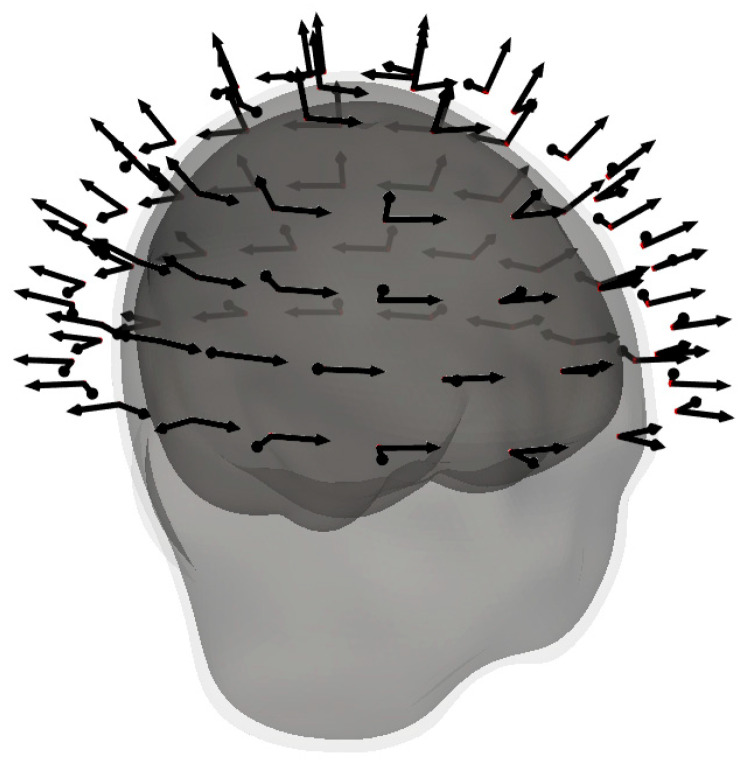
The hypothetical whole-head OPM-MEG system, where each arrow represents a sensing direction. The gray surfaces represent reconstructed BEM surfaces for one subject.

The hypothetical whole-head OPM-MEG system comprises 80 sensors with two orthogonal sensing directions (radial and tangential). This sensor geometry of the OPM-MEG system is the same for all subjects. Figure 1 shows a 3D image of the OPM-MEG system with BEM surfaces for one example subject. The geometry of the OPM-MEG system was generated by joining the reconstructed outer surfaces of the heads of all subjects together. We then fitted circles to cover all the heads at different heights. OPM sensors were equidistantly placed on these circles. The radial component of the sensor was oriented towards the center of the lowest circle, and the sensors were pushed inward toward this center to be as close to the joint outer surfaces as possible. For the bottom row of sensors, the minimal distance from the center to the sensor is 8.9 cm, and the maximum distance is 12.1 cm; the average distance is 10.0 cm. For comparison, if we fit a circle to the bottom row of the Yokogawa SQUID system sensors (only the closer magnetometer from the gradiometer pair), the minimal distance from the center to the sensor is 11.6 cm, and the maximum distance is 12.8 cm; the average distance is 12.1 cm.

### 2.3. Sequential Selection Algorithm (SSA)

**Figure 2:**
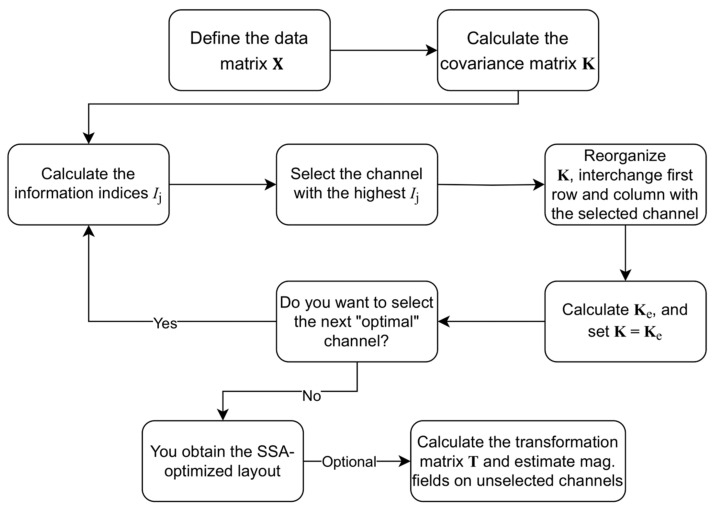
Schematic flowchart of the sequential statistical approximation (SSA) algorithm for optimal channel selection. Selecting optimal channels with SSA begins with the computation of the correlation matrix C and the covariance matrix K from the magnetic field data matrix X. In each iteration, an information index $I_{j}$ is computed for every non-selected channel to quantify its overall correlation with other channels. The channel with the highest $I_{j}$ is selected and repositioned to the top-left corner of K(i.e., the first row and column). The matrix is then partitioned into four blocks, and the covariance of estimated error $K_{e}$ is calculated. For choosing the next channel, $K_{e}$ is set as K, and the loop is repeated until the desired number of channels is selected. After channel selection, a transformation matrix T can be computed to estimate the magnetic field at the unselected channels.

The algorithm is based on a sequential statistical comparison of non-selected channels. This algorithm is based on work by Lux and colleagues [24,33,34,35]. The schematic overview of the SSA procedure is in Figure 2. We first define a data matrix X, which consists of M recorded magnetic field maps (MFMs). Each MFM represents measurements from N channels at a specific time point t. Consequently, each element of this matrix corresponds to a measured magnetic field value: $X_{i,k}=B_{i}(t_{k})$, where index i denotes the individual channels (i=1,2,…,N), and k represents the time instance of each MFM (k=1,2,…,M). From X, we compute the correlation matrix C and the covariance matrix K to assess channel relationships:

(1) $$K_{ij}=\sigma_{i}\sigma_{j}C_{ij}=\frac{1}{M}\sum_{k=1}^{M}\left(X_{i,k}-{\bar{X}}_{i}\right)\left(X_{j,k}-{\bar{X}}_{j}\right),$$

where $\sigma_{i}$ represents the standard deviation:

(2) $$\sigma_{i}^{2}=\frac{1}{M}\sum_{k=1}^{M}{\left(X_{i,k}-{\bar{X}}_{i}\right)}^{2}.$$

The trace of the covariance matrix tr(**K**), which is the sum of variances ($\sum_{i}\sigma_{i}^{2}$), represents the total statistical power in the entire N-dimensional space of measurement channels.

The optimal channel selection follows an iterative approach (as illustrated in Figure 2). For each channel j, we determine an information index [24]:

(3) $$I_{j}=\sum_{i=1}^{N}\sigma_{i}^{2}C_{ij}^{2}=\sum_{i=1}^{N}K_{ij}^{2}/\sigma_{j}^{2},$$

which indicates how much the j-th channel is, on average, correlated with all other measurement channels. The channel with the highest $I_{j}$ value is identified as the most informative and selected. The matrix K is then reorganized by placing the selected channel’s index in the first row and column without reordering the full matrix by $I_{j}$ values. The restructured matrix is partitioned into four blocks:

(4) $$K=\left(\begin{array}{cc} K_{11} & K_{12}\\ K_{21} & K_{22} \end{array}\right),$$

where $K_{11}$ is of size 1 × 1. Then the covariance of estimated error $K_{e}$ is calculated [24]:

(5) $$K_{e}=K_{22}-{K_{12}}^{T}{K_{11}}^{-1}K_{12}.$$

Next, we set $K=K_{e}$ (dimension is reduced by 1)**,** recompute the information indices, and select the next optimal channel. The procedure continues iteratively until the required number of channels is chosen ($N_{s}$).

Once we finish with channel selection, we can calculate the transformation matrix T. This matrix allows us to estimate the expected values of the magnetic field $X^{e}$ at the unselected measuring channels based on the measurements $X^{s}$ from the selected channels [24]:

(6) $$X^{e}=K_{\mathrm{us}}K_{ss}^{-1}X^{s}=TX^{s}.$$

This formula was derived using the least mean-squared criterion for linear transformation that estimates data on unselected channels from the data measured with the selected channels. The matrix T represents a linear transformation defined by the product of the inverse of the covariance matrix $K_{ss}$ of the selected (measured) channels and the cross-covariance matrix $K_{us}$ between the selected and unselected channels. A brief summary of the SSA procedure above is in figure cation of the SSA selection flowchart (Figure 2).

During the SSA iteration, we can monitor two measures. Using $K_{e}$, we can calculate the RMS (root mean square) error as:

(7) $${\mathrm{RMS}}_{\mathrm{err}}=\sqrt{\frac{tr\left(K_{e}\right)}{N-1}},$$

where $tr(K_{e})$ is the trace of the matrix $K_{e}$. Additionally, we can calculate the relative statistical power:

(8) $$\mathrm{Rel}.\;\mathrm{stat}.\;\mathrm{power}=\frac{tr\left(K\right)-tr\left(K_{e}\right)}{tr\left(K\right)},$$

which reflects the proportion of the total statistical power that is contained in the selected $N_{s}$-dimensional subspace relative to the entire N-dimensional space.

The value of the total statistical power tr(K) depends on the database selected for training (the “training database”), specifically the number of MFMs (M) and their variances. The variances are expected to be highest for MFM near the strongest responses, particularly around M100.

### 2.4. Protocols for Applying the SSA on OPM-MEG MFMs

In our work, we consider four different systems. One system is the real SQUID MEG system with which we have made measurements, and the other three systems are hypothetical and based on OPMs, the geometry of which is presented in Section 2.2. Our potential OPM-MEG system consists of magnetometers measuring radial and tangential components relative to the head surface. We investigate the applicability of the SSA algorithm when considering only radial (OPM-RAD; 80 channels), only tangential (OPM-TAN; 80 channels), or both components simultaneously (OPM-2AX; 160 channels).

Several approaches for the OPM-2AX system are possible for applying the SSA algorithm.

- We treat the radial and tangential channels as independent, giving 160 channels, 80 radial and 80 tangential. This approach selects $N_{s}$ channels using the SSA algorithm.
- During the SSA selection, we chose channels (same as with approach I). Finally, we add the channel pair (exact location, different measuring component) that has not yet been selected. This gives $N_{m}$ of selected measurement sites and $N_{s}=2\;N_{m}$ channels.
- This approach is a combination of approaches I and II. When we select one channel (radial or tangential) during the SSA, we also choose the channel pair. Similarly, as II, this gives us $N_{m}$ of selected measurement sites and $N_{s}=2\;N_{m}$ channels.
- With this approach, we combine the radial and tangential MFMs into a common basis with twice the number of all MFMs. We consider the measurement system to be 80-channel. The transfer matrix (T) in Equation (6) is the same for radial and tangential channels.

For calculating results in Section 3, we use approach III; the results for other approaches are presented in Supplementary Material S.1.

### 2.5. Evaluation Parameters

#### 2.5.1. Root-Mean-Square Error, Relative Difference, and Correlation Coefficient

Diagnostic information contained in MFMs has yet to be fully elucidated; however, the differences in signal information can be quantified using the following parameters: average RMS (root mean square) errors (9), average relative differences (RD) (10) and average correlation coefficients (CC) (11) between measured MFM $Y^{m}$ and estimated $Y^{e}$ on different time intervals $t_{j}\in \left[0,400\right]$, $t_{j}\in \left[42,240\right]$, $t_{j}\in M100\pm 12$, and $t_{j}\in M50\pm 6\;\mathrm{ms}$:

(9) $$\mathrm{RMS}=\frac{1}{M}\sum_{j=1}^{M}\mathrm{RMS}\left(t_{j}\right),\;\mathrm{where}\;\mathrm{RMS}\left(t_{j}\right)=\sqrt{\sum_{i=1}^{N_{u}}{\left(Y_{i,j}^{e}-Y_{i,j}^{m}\right)}^{2}/N_{u}};$$

(10) $$\mathrm{RD}=\frac{1}{M}\sum_{j=1}^{M}\mathrm{RD}\left(t_{j}\right),\;\mathrm{where}\;\mathrm{RD}\left(t_{j}\right)=\sqrt{\sum_{i=1}^{N_{u}}{\left(Y_{i,j}^{e}-Y_{i,j}^{m}\right)}^{2}/\sum_{i=1}^{N_{u}}{\left(Y_{i,j}^{m}\right)}^{2}};$$

(11) $$\mathrm{CC}=\frac{1}{M}\sum_{j=1}^{M}\mathrm{CC}\left(t_{j}\right),\;\mathrm{where}\;\mathrm{CC}\left(t_{j}\right)=\sum_{i=1}^{N_{u}}Y_{i,j}^{e}Y_{i,j}^{m}/\sqrt{\sum_{i=1}^{N_{u}}{\left(Y_{i,j}^{e}\right)}^{2}{\left(Y_{i,j}^{m}\right)}^{2}},$$

where $N_{u}$ denotes the number of unselected measuring sites.

#### 2.5.2. Localization Error

**Figure 3:**
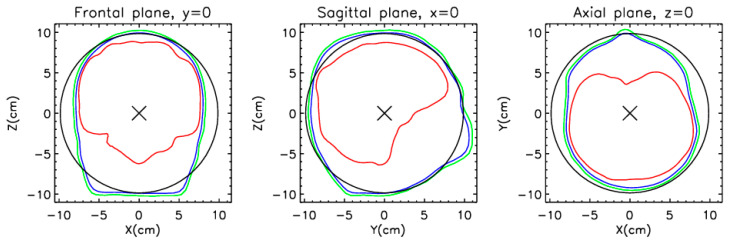
Homogeneous conducting sphere model—three planes of intersection. The black circle represents the sphere’s edge, the green and blue the outer and inner sides of the skull, and the red the brain surface (see Figure 1). The origin of the coordinate system (×) is in the sphere’s center.

To check the impact of the number of measurement sites on the source localization, we fitted either one or two equivalent current dipoles (ECD). As a direct model, we have used a model that assumes that we have an ECD inside a homogeneous spherically symmetric volume conductor. The magnetic field around a spherically symmetric volume conductor generated by a current dipole can be calculated analytically [36]. The equation is given in Appendix A.1. Despite the significant assumptions made in this model, the result of the direct model is usable for MEG source localization. Since, in this work, we are not interested in the exact sources in the brain, but only in how the localization error varies with the selected sensors relative to the case where all sensors are considered, this model is perfectly sufficient. It is interesting to note that the solution is independent of the conductivity and the sphere’s size, and the current dipole’s radial component does not contribute to the magnetic field outside the sphere. The placement of the sphere’s center is important because this affects the calculated field. In this study, we have determined the sphere that most closely matches the outer surface of the head. Figure 3 shows a model of a homogeneous conducting sphere in three intersection planes: frontal, sagittal, and axial.

To solve the inverse problem, we use the following methodology. At a specific time t, we have measured values $B=(B_{1},\ldots ,B_{N})$ of N channels. We assume that the source is one or two ECDs. We can write B as the sum of contributions from the first and second ECD and the residual ε:

(12) $$B=L({\vec{r}}_{p_{1}})\cdot \vec{p_{1}}+L({\vec{r}}_{p_{2}})\cdot \vec{p_{2}}+\varepsilon ,$$

where L is the lead-field matrix, which includes the direct model; ${\vec{r}}_{p}$ represents the location of the ECD and $\vec{p}$ the direction and intensity of the ECD. In the case where only one dipole is assumed, the contribution of the other dipole is neglected ($\left|\vec{p_{2}}\right|=0$). We want to estimate (fit) the parameters ${\vec{r}}_{p_{1}}$, $\vec{p_{1}}$, ${\vec{r}}_{p_{2}}$, and $\vec{p_{2}}$ that the difference between the measured magnetic field (B) and the values predicted by the model (9) is minimal. We solve this by applying a non-linear least squares curve fitting using the Levenberg–Marquardt algorithm (LMA) [37,38,39,40]. The main disadvantage of this algorithm is that the result of finding the global minimum can be trapped in the local minimum. This is avoided by choosing good initial conditions. We calculate the initial conditions by fixing the locations of ${\vec{r}}_{{p_{1}}_{0}}$ and ${\vec{r}}_{{p_{2}}_{0}}$ (one in left and one in right hemisphere). So, we are only looking for the parameters $\vec{p_{1}}$ and $\vec{p_{2}}$. We use the solutions of this linear problem as the initial conditions for solving LMA.

## 3. Results

### 3.1. Interval with the Largest Statistical Power

**Figure 4:**
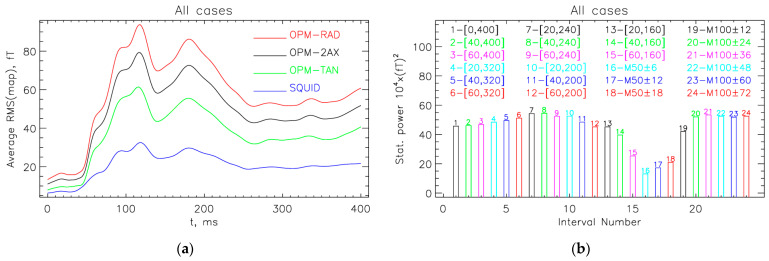
(**a**) Average RMS values of auditory evoked MFMs for different measurement systems: OPM-2AX (dual-axis OPM sensors—160 channels), OPM-RAD (radial OPM sensors—80 channels), OPM-TAN (tangential OPM sensors—80 channels), and SQUID (gradiometers in radial direction—125 channels). Notably, the SQUID MFMs represent real measurements, whereas the data for the OPM systems were derived through the transformation of the SQUID recordings. The RMS values begin to increase approximately 40 ms after the onset of acoustic stimulation, peaking around 100 ms (M100). (**b**) Statistical power of the OPM-2AX system across different time intervals. The highest statistical power is observed between 40 and 240 ms, aligning with the typical M100 response window and clinical relevance. Both figures (**a**,**b**) are averaged for all 16 measurements.

Figure 4a compares the average RMS of MFM values for different measurement systems. The results show that the RMS starts to increase about 40 ms after the start of the acoustic stimulation, reaching a peak around 100 ms (M100). The comparison between systems shows that, on average, the signals are highest for the radial components of the OPM (OPM-RAD) and about one-third lower for the tangential components (OPM-TAN). As expected, the SQUID signals are smaller, about one-third of the OPM-RAD value. This is mainly due to the greater distance from the brain sources, as the SQUID sensors have to be cooled with liquid helium, and partly due to the gradiometer configuration of the SQUID system (the baseline length of axial-type gradiometers is 5 cm).

**Figure A1:**
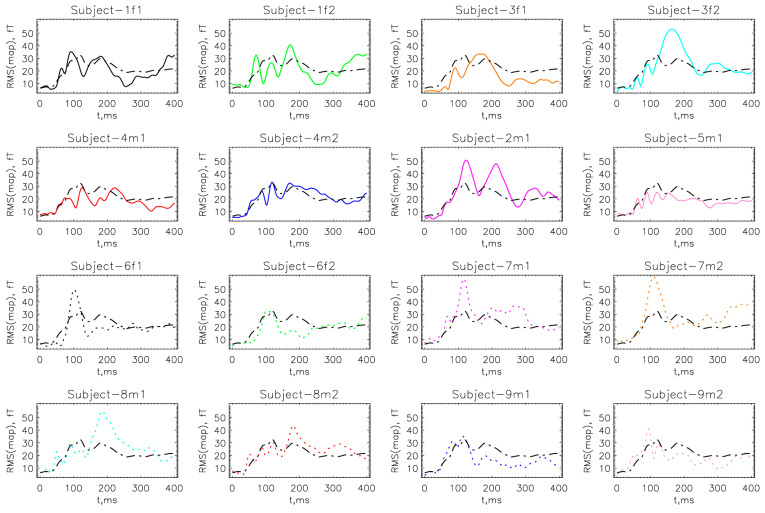
Each subject’s average RMS values of MFMs for the SQUID system. The individual cases are shown with different colors and lines (the first 8 with solid lines and the second 8 with dashed lines). The average RMS of all measured MFM from Figure 4a are displayed with a bold dash-dot-dashed line.

We analyzed measurements for individual subjects (Figure A1); we noticed that the RMS (map) values of M100 responses vary considerably from person to person. The highest peak is for Subject 7m, about twice as high as the average. For some subjects, a peak appears later (M200), which may be even higher than the M100 (3f, 8m) or comparable to the M100 (1f, 2m, 4m). For most cases, we also observe a weak first response (M50), which for some subjects (1f, 4m, 5m) is comparable to the M100.

Figure 4b shows the statistical power for the OPM-2AX system at different time intervals calculated for all 16 measurements. The highest statistical power is obtained if MFMs on the time interval between 40 and 240 ms are included, which is also the interval of clinical interest [17,18,19,20,21,22]. This is also consistent with the average RMS(map) results in Figure 4a. We wanted a round number of MFMs per subject. Our datasets have a sampling frequency of 500 Hz; therefore, we chose a slightly modified interval between 42 and 240 ms to obtain 100 MFMs per subject, 1600 MFMs in total.

### 3.2. Optimal Locations for the OPM-2AX System

**Figure 5:**
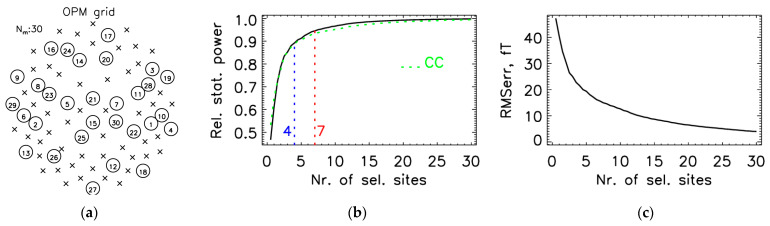
The selection procedure for the first 30 measurement sites (60 channels) for the OPM-2AX system. The SSA selection was made using the entire database (all 16 measurements) in the time interval from 42 to 240 ms (a total of 1600 MFMs), using protocol III for SSA (Section 2.4). (**a**) OPM grid with selected (circle) and unselected (cross) measuring sites: the circled number represents the selection priority during the SSA selection iterations. (**b**) Relative statistical power as a function of the number of selected measurement sites; the curve demonstrates that the statistical power exceeds 0.90 and 0.95 after the fourth and seventh selected sites, respectively; or reference, the CC (correlation coefficient) value obtained during training is indicated with a green dashed line. (**c**) RMS error (RMSerr) as a function of the number of selected measurement sites: both measures (**b**) and (**c**) were calculated during the SSA selection process.

**Figure 6:**
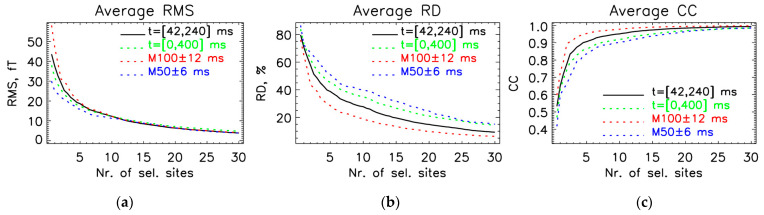
Average evaluation parameters were calculated between the values of measured and estimated (6) data on unselected measurement sites: (**a**) RMS (9), (**b**) RD (10), and (**c**) CC (11) for the parameter “number of selected sites” for four different time intervals. The time interval for SSA selection was from 42 to 240 ms. The results are for the OPM-2AX system, using protocol III for SSA selection. The parameters were averaged using the entire database (all 16 measurements).

For SSA protocol III (Section 2.4), which proved to be the best, we show results obtained on the whole database at an interval between 42 and 240 ms, i.e., a total of 1600 160-channel MFMs. Figure 5a shows the distribution of the first 30 selected measurement sites. Figure 5b shows the relative statistical power (8) and Figure 5c RMSerr (7) as a function of the number of selected measurement sites. Figure 6a shows the evaluation parameters for different time intervals. For Figure 6, the evaluation measures were calculated between the measured and estimated data on unselected measurement sites. The estimated values were obtained using Equation (6) from the SSA method. For comparison, the measure CC (11) at the learning interval of [42, 240] ms from Figure 5b is also shown in Figure 6c with a green dashed line; the two curves approximately match. The relative statistical power exceeds 0.90 and 0.95 after the fourth and seventh optimally chosen measurement sites. The exact values of average evaluation parameters RMS, RD, and CC for different numbers of selected channels for different learning intervals are stated in Table 2.

### 3.3. Localization of Sources for the M50 and M100 Peaks

**Figure 7:**
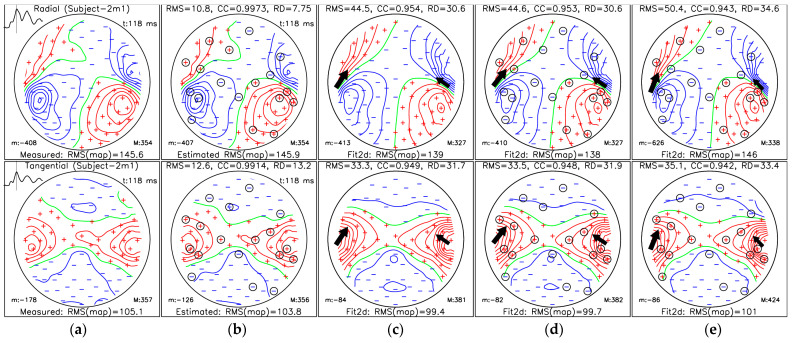
Magnetic field maps for the M100 AEF peak (at 118 ms) of Subject-2m1: (**a**) measured data (reference map); (**b**) SSA-estimated data; (**c**) reconstructed data using dual dipole fit (Fit2d) of measured data; (**d**) reconstructed data using dual dipole fit of SSA-estimated data; (**e**) reconstructed data using dual dipole fit of measured data using only $N_{m}=18$ selected sites. The top row shows the radial component, and the bottom row shows the tangential component of the magnetic field. Panels (**c**)–(**e**) present dipole fit reconstructions, with RMS error, correlation coefficient (CC), and relative difference (RD%) reported for each, in comparison to the reference map (**a**). The circled signs in (**b**,**d**,**e**) represent SSA-selected sites. “m:” indicates the minimum, and “M:” is the maximum value of the MFM. Positive, zero, and negative isolines are presented with red, green, and blue lines, respectively. Measuring sites with positive and negative field values are labelled with red plus and blue minus symbols, respectively.

**Figure 8:**
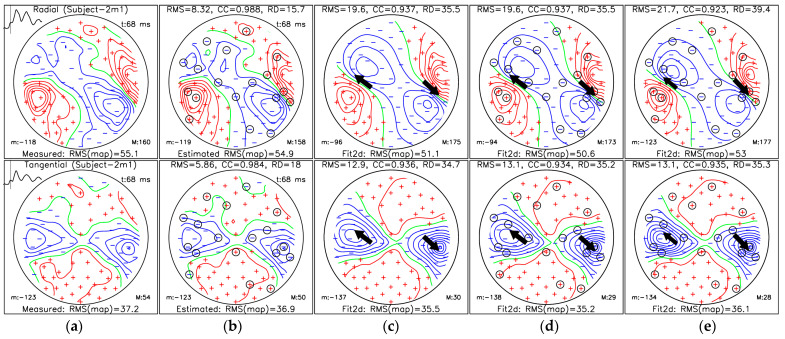
Magnetic field maps for the M50 AEF peak (at 68 ms) of Subject-2m1: (**a**) measured data (reference map)**;** (**b**) SSA-estimated data; (**c**) reconstructed data using dual dipole fit (Fit2d) of measured data; (**d**) reconstructed data using dual dipole fit of SSA-estimated data; (**e**) reconstructed data using dual dipole fit of measured data using only $N_{m}=18$ selected sites. The top row shows the radial component, and the bottom row shows the tangential component of the magnetic field. Panels (**c**–**e**) present dipole fit reconstructions, with RMS error, correlation coefficient (CC), and relative difference (RD%) reported for each, in comparison to the reference map (**a**). The circled signs in (**b**,**d**,**e**) represent SSA-selected sites. “m:” indicates the minimum, and “M:” is the maximum value of the MFM. Positive, zero, and negative isolines are presented with red, green, and blue lines, respectively. Measuring sites with positive and negative field values are labelled with red plus and blue minus symbols, respectively.

The results of ECD fits (Section 2.5.2), as well as measured and SSA-estimated MFMs, for subject 2m1 are shown in Figure 7 (M100 AEF peak) and Figure 8 (M50 AEF peak). Figures for other subjects and cases are shown in Supplementary Materials S.2. In Figure 7 and Figure 8, we show five topographical maps of:

(a) MFM for measured data;
(b) MFM of estimated data from $N_{m}=18$ selected measuring sites (36 channels) using protocol III for the OPM-2AX system;
(c) MFM estimated for the ECD fit using measured data from all measuring sites;
(d) MFM estimated for the ECD fit using SSA-estimated data from $N_{m}=18$ selected measuring sites;
(e) MFMs estimated for the ECD fit using data only from selected measuring sites.

We selected the $N_{m}=18$ to ensure that the CC value for the interval [42, 240] ms exceeded 95% (Table 2).

In Figure 7 and Figure 8, each topographical map is labeled with the MFM type and the RMS value of the whole map (RMS(map)) in the subheading; the minimum value of the magnetic field is indicated by “m:” in the bottom left and the maximum value of the MFM is indicated by “M:” in the bottom right. All field values are in fT units. The time in ms for the MFM is shown in the top right corner. On the measured MFM, the RMS values of the maps are plotted on the top left over the whole time interval between 0 and 400 ms; the vertical bar indicates the time of the displayed folder. For the measured MFM, the data type (radial and tangential) is displayed in the title, and the subject code is in brackets. The header shows the quantitative comparison with the measured map: RMS, CC, and RD in % (panels (c)–(e)). In panels (c)–(e)**,** the fitting results (location and moment of one or two current dipoles) are also displayed.

Table 3 and Table 4 state the details of the dual dipole fit presented in Figure 7 (M100 AEF peak) and 7 (M50 AEF peak). In the tables, we have the following dipole parameters: dipole locations. ${\vec{r}}_{p1}$, ${\vec{r}}_{p2}$; magnitudes and directions ${\vec{p}}_{1}$, ${\vec{p}}_{2}$. Additionally, we calculate the following differences measures in comparison to the dipole fit of the reference case (panel (**c**)): localization errors $∆r_{1}$ and $∆r_{2}$, which are the distances of the first and second dipoles from the reference; Euclidean norm for both localization errors $∆r_{c}=\sqrt{∆r_{1}^{2}+∆r_{2}^{2}}$; and direction errors $∆\varphi_{1}$ and $∆\varphi_{2}$ (angles between directions).

**Figure 9:**
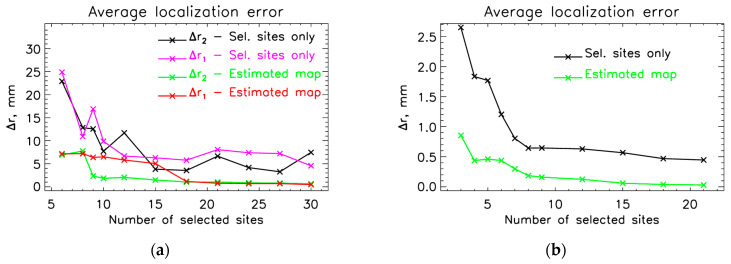
Average localization error for a different number of selected sites using the reference case (dipole fit with all measuring sites). The localization error was calculated for two cases: using channels from both (**a**) or from the right (**b**) hemisphere. $\Delta r_{1}$ and $\Delta \;r_{2}$ are calculated for two cases: using only selected and using selected and SSA-estimated measuring sites.

We have extended the fitting results to see how the localization error changes with the number of selected measuring sites (Figure 9). Some measurements do not show simultaneous AEF response in both hemispheres, and therefore, the ECD can be successfully fitted on only one side of the brain. The details about the SSA selections from the 43 sites that cover the right hemisphere can be found in Supplementary Materials S.1.6. We calculated the ECD fits using channels only from the right hemisphere (Figure 9b) and channels from both hemispheres (Figure 9a). The results are compared for two cases: fitting ECDs on the SSA estimated map and fitting ECDs using only the selected sites. For Figure 9a, we used data from 10 measurements. For this case, the corresponding values of the mean localization error with standard deviation and the average RMS, RD, and CC for the estimated M100 maps are in Table 5. Corresponding values for Figure 9b are in Table 6. Results of the evaluation of estimated M100 displayed in Table 6 show that, on average, the CC is above 0.97, and the localization error is below 0.5 mm after only 6 SSA-selected sites.

In Supplementary Materials S.3, we displayed results for the M100 and M50 peaks for all measurements using nine selected sites from the right hemisphere.

## 4. Discussion

Our study demonstrates that SSA is an effective tool for optimizing the layout of OPM-MEG systems with a limited number of sensors. We identified sensor placements that maximize information capture while reducing redundancy by applying SSA to OPM-MEG datasets, which were transformed from the SQUID-MEG system measurements of AEF [12].

Our findings indicate that a relatively small number of optimally placed OPM sensors can approximate the information captured by full-head SQUID-MEG recordings. Specifically, the first 15 to 20 optimally selected sites achieved CC greater than 0.95, with localization errors below 1 mm (Figure 6 and Figure 9). This suggests that for event-related fields (ERFs), such as AEFs, which primarily originate from focal sources, a significantly smaller number of sensors than conventional MEG systems would be sufficient for accurate source localization and MFM estimation. This is even more pronounced when we use AEF data from only one hemisphere (Figure 9b and Table 6), where the CC measure was on average above 0.97 and the localization error was below 0.5 mm after only 6 optimal sites selected with SSA protocol III.

The main difference between protocols II (Figures S.3 and S.4), III (Figure 5 and Figure 6), and IV (Figures S.9 and S.10) is in the selection of the first 10–15 leads, after which the average RMS, RD, and CC are similar. These results corroborate the finding of Lux et al. [24] that the “optimal” lead selection is not unique, i.e., a slight shift in the position of the first few leads can produce quite different lead sets that perform equally well. We obtained the best results for the first 10–15 selected sites with protocol III, where the relative statistical power exceeds 0.90 and 0.95 after only the 4th and 7th “optimal” selection (Figure 4), for protocol II after the 6th and 9th (Figure S.3), and for protocol IV (Figure S.10) after the 7th and 12th, respectively. Therefore, we used protocol III as the default approach in our study.

The ability of SSA to effectively optimize OPM sensor layouts has substantial implications for the development of cost-effective, portable MEG systems. Commercial OPM sensors remain expensive, and reducing the required number of sensors while maintaining measurement quality can facilitate broader adoption, particularly in clinical and research environments with budgetary constraints. Moreover, by enabling subject-specific sensor layouts, SSA could enhance individualized MEG recordings, improving both signal quality and patient comfort.

Furthermore, the ability of SSA to incorporate both radial and tangential sensor components suggests that dual-axis OPM-MEG configurations offer significant advantages. Our results show that incorporating both components results in more accurate reconstructions of MFMs, particularly when using Protocol III for SSA selection.

Several limitations must be considered when interpreting our results. First, our study relies on transformed SQUID-MEG data rather than direct full-head OPM-MEG measurements. While the transformation methodology using MNE has been validated in previous studies, minor discrepancies may exist between actual and estimated OPM signals. This transformation provides a useful proxy for assessing potential gains in sensor placement and signal detection; however, it may not fully replicate the distinct characteristics of OPM-MEG systems, including differences in sensor geometry, spatial arrangement constraints, motion robustness, and dynamic range. As such, future studies should incorporate empirical data from full-head OPM-MEG measurements, enabling direct validation of the SSA-optimized layouts. Second, the SSA approach assumes that the selected sensor locations remain fixed throughout the recording session. However, in practice, subject movement and variations in sensor placement may introduce additional variability. Future studies should explore how this would affect SSA results and develop strategies to account for such variations.

Finally, while our study focused on AEFs, further investigation is needed to determine the generalizability of our findings to other types of brain activity, such as event related fields from other brain regions, spontaneous oscillations, or pathological activity in clinical populations (e.g., epilepsy).

Future research should validate these findings with direct full-head OPM-MEG measurements and assess the impact of SSA-optimized sensor layouts in practical experimental and clinical settings. Additionally, expanding SSA to incorporate real-time feedback for sensor placement optimization could further enhance the utility of OPM-MEG in applications requiring flexible measurement setups.

Moreover, investigating the integration of SSA with machine learning approaches may provide more robust sensor selection strategies, potentially allowing for automated, data-driven optimization of sensor configurations based on individual subject characteristics.

Another potential future research could be the utilization of MFMs for analyzing AEFs. Like body surface potential mapping in electrocardiography [41,42,43,44,45,46,47,48,49,50,51,52,53,54] and magnetic field mapping in magnetocardiography [55,56,57,58,59], MFMs derived from MEG measurements could enable the precise visualization of spatiotemporal neural dynamics. By leveraging MFMs, researchers could potentially track dynamic changes in neural activity and improve the identification of abnormal auditory processing patterns in clinical populations. Future studies should explore the integration of MFM-based analysis with SSA-optimized sensor layouts to enhance both the resolution and interpretability of OPM-MEG auditory response measurements.

## 5. Conclusions

This study shows the potential of SSA for optimizing OPM-MEG sensor layouts, demonstrating that a limited number of strategically placed sensors can effectively capture critical neural activity. By reducing hardware costs and increasing measurement flexibility, SSA-based sensor selection could facilitate the broader adoption of OPM-MEG technology across neuroscience and clinical applications.

## Figures and Tables

**Table 1:** List of recordings.

N	Recording ^1^	Date	Age
1	Subject-1f1	8 October 2018	30
2	Subject-1f2	7 May 2019	31
3	Subject-2m1	16 October 2019	26
4	Subject-3f1	27 June 2019	31
5	Subject-3f2	10 April 2019	31
6	Subject-4m1	9 October 2018	36
7	Subject-4m2	10 May 2019	37
8	Subject-5m1	8 January 2020	31
9	Subject-6f1	5 July 2018	33
10	Subject-6f2	5 April 2019	34
11	Subject-7m1	7 May 2019	54
12	Subject-7m2	17 October 2019	54
13	Subject-8m1	11 October 2019	26
14	Subject-8m2	18 October 2019	26
15	Subject-9m1	18 June 2018	57
16	Subject-9m2	12 June 2019	58

^1^ f—female, m—male.

**Table 2:** Average evaluation parameters RMS, RD, and CC for different numbers of selected sites ($N_{m}=12,\;18,\;24,\;and\;30$) for different learning intervals. The results are for the OPM-2AX system, using protocol III for SSA.

$N_{m}=12$	[0, 400] ms	[42, 240] ms	M100 ± 12 ms	M50 ± 6 ms
RMS [fT]	15.4 ± 4.2	16 ± 4.2	16.2 ± 3.9	13.6 ± 6.9
RD [%]	39.4 ± 18.6	32.7 ± 15.3	23.2 ± 10.3	44 ± 18.4
CC	0.897 ± 0.115	0.932 ± 0.076	0.968 ± 0.031	0.875 ± 0.108
$N_{m}=18$				
RMS [fT]	12.4 ± 3.2	12.4 ± 3.1	13 ± 3	11.2 ± 3.9
RD [%]	32.5 ± 16.8	25.8 ± 13.1	18.4 ± 7.9	37.3 ± 16.2
CC	0.929 ± 0.087	0.957 ± 0.055	0.98 ± 0.02	0.914 ± 0.077
$N_{m}=24$				
RMS [fT]	10 ± 2.7	9.5 ± 2.2	9.7 ± 2.4	9.5 ± 3.7
RD [%]	26.5 ± 14.9	20.1 ± 11.1	13.7 ± 5.3	31.7±13.6
CC	0.952 ± 0.065	0.973 ± 0.039	0.989 ± 0.009	0.939 ± 0.053
$N_{m}=30$				
RMS [fT]	8.3 ± 2.1	7.7 ± 1.5	7.9 ± 1.8	7.9 ± 2.4
RD [%]	22.6 ± 13.6	16.4 ± 9.8	11.1 ± 3.8	27.2 ± 12.8
CC	0.964 ± 0.051	0.981 ± 0.03	0.993 ± 0.005	0.954 ± 0.046

Results for other protocols and systems are shown in Supplementary Materials S.1.

**Table 3:** Dual ECD fit results for the M100 AEF peak (Figure 7). Dipole parameters include locations (${\vec{r}}_{p1}$, ${\vec{r}}_{p2}$) and magnitudes/directions (${\vec{p}}_{1}$, ${\vec{p}}_{2}$). Differences from the reference fit (Figure 7c) are quantified by localization errors ($∆r_{1}$, $∆r_{2}$), their Euclidean norms ($∆r_{c}$), and direction errors $∆\varphi_{1}$, $∆\varphi_{2}$).

Measures	Measured Map Fit (c)	Estimated Map Fit (d)	Selected Chan. Fit (e)
${\vec{r}}_{p1}$ [mm]	(44.5, 0.9, 15.2)	(44.8, 0.9, 15.4)	(51.8, −1.1, 14.6)
${\vec{r}}_{p2}$ [mm]	(−37.8, 3.0, 6.2)	(−38.2, 4.1, 7.6)	(−41.6, 0.4, 5.3)
${\vec{p}}_{1}$ [µAm]	(10.5, −12.1, −30.1)	(10.4, −11.4, −29.6)	(5.9, −11.3, −22.0)
${\vec{p}}_{2}$ [µAm]	(−8.1, −26.3, −36.7)	(−9.4, −23.2, −34.8)	(−3.8, −25.9, −28.1)
$∆r_{1}$ [mm]	/	0.3	7.5
$∆r_{2}$ [mm]	/	1.9	4.7
$∆r_{c}$ [mm]	/	1.9	8.9
$∆\varphi_{1}$ [°]	/	0.8	6.8
$∆\varphi_{2}$ [°]	/	3.2	8.3

**Table 4:** Dual ECD fit results for the M50 AEF peak (Figure 8). Dipole parameters include locations (${\vec{r}}_{p1}$, ${\vec{r}}_{p2}$) and magnitudes/directions (${\vec{p}}_{1}$, ${\vec{p}}_{2}$). Differences from the reference fit (Figure 8c) are quantified by localization errors ($∆r_{1}$, $∆r_{2}$), their Euclidean norms ($∆r_{c}$), and direction errors $∆\varphi_{1}$, $∆\varphi_{2}$).

Measures	Measured Map Fit (c)	Estimated Map Fit (d)	Selected Chan. Fit (e)
${\vec{r}}_{p1}$ [mm]	(49.7, −6.4, 27.3)	(50.9, −6.6, 26.1)	(51.2, −5.1, 28.7)
${\vec{r}}_{p2}$ [mm]	(−34.1, 2.1, 41.7)	(−35.3, 2.0, 42.8)	(−43.6, 0.3, 44.8)
${\vec{p}}_{1}$ [µAm]	(−3.2, 2.9, 6.5)	(−2.8, 2.6, 6.2)	(−2.7, 3.0, 5.4)
${\vec{p}}_{2}$ [µAm]	(5.4, −3.3, 4.6)	(5.1, −3.1, 4.3)	(3.6, −2.4, 3.5)
$∆r_{1}$ [mm]	/	1.7	2.4
$∆r_{2}$ [mm]	/	1.7	10.2
$∆r_{c}$ [mm]	/	2.4	10.5
$∆\varphi_{1}$ [°]	/	1.6	5.0
$∆\varphi_{2}$ [°]	/	0.2	3.8

**Table 5:** Average localization error [mm] with standard deviations and evaluation parameters (RMS [fT], RD [%], and CC) of SSA-estimated M100 MFMs.

	Evaluation of Estimated M100			Localization— Estimated		Localization— Sel. Sites Only	
$N_{m}$	RMS±SD [fT]	RD±SD [%]	CC±SD	$∆r_{1}\pm SD$ [mm]	$∆r_{2}\pm SD$ [mm]	$∆r_{1}\pm SD$ [mm]	$∆r_{2}\pm SD$ [mm]
6	17.3 ± 4.4	22.6 ± 10.1	0.971 ± 0.026	7.2 ± 8.6	6.9 ± 4.4	24.9 ± 23.7	22.9 ± 16.6
8	14.8 ± 3.4	19 ± 7.5	0.980 ± 0.015	7.2 ± 10.6	7.7 ± 13.1	10.9 ± 8.5	12.8 ± 18.1
9	13.7 ± 3.4	17.6 ± 7.2	0.983 ± 0.015	6.4 ± 10.7	2.3 ± 2.0	16.9 ± 11.8	12.5 ± 16.2
10	12.3 ± 3.1	16.4 ± 6.2	0.985 ± 0.013	6.5 ± 11.8	1.8 ± 1.1	9.9 ± 8.4	7.7 ± 7.0
12	10.2 ± 2.9	13.5 ± 4.6	0.990 ± 0.006	5.8 ± 12.3	2.0 ± 1.2	6.7 ± 4.7	11.7 ± 15.7
15	8.7 ± 2.2	11.3 ± 3.4	0.993 ± 0.004	5.0 ± 12.2	1.5 ± 1.2	6.3 ± 5.0	3.8 ± 3.4
18	7.3 ± 1.8	9.3 ± 3.0	0.995 ± 0.003	1.1 ± 1.2	1.0 ± 0.8	5.8 ± 4.9	3.5 ± 2.0
21	6.1 ± 1.3	7.9 ± 2.5	0.997 ± 0.002	0.8 ± 0.7	1.0 ± 1.0	8.1 ± 7.8	6.6 ± 7.6
24	5.1 ± 1.0	6.6 ± 2.1	0.998 ± 0.001	0.7 ± 0.5	0.8 ± 0.9	7.4 ± 8.0	4.2 ± 3.4
27	4.6 ± 1.0	5.8 ± 1.8	0.998 ± 0.001	0.7 ± 0.5	0.8 ± 0.7	7.2 ± 7.9	3.3 ± 2.6
30	4.1 ± 0.8	5.4 ± 1.9	0.998 ± 0.001	0.5 ± 0.3	0.6 ± 0.6	4.6 ± 3.3	7.5 ± 14.1

**Table 6:** Average localization error [mm] with standard deviations and evaluation parameters (RMS [fT], RD [%], and CC) of SSA-estimated M100 MFMs for sensors on the right hemisphere only.

	Evaluation of Estimated M100			Estimated	Selected
$N_{m}$	RMS±SD [fT]	RD±SD [%]	CC±SD	∆r±SD [mm]	∆r±SD [mm]
3	25.5 ± 6.7	32.3 ± 14.3	0.946 ± 0.047	0.9 ± 0.5	2.7 ± 3.1
4	19.2 ± 3.7	25.4 ± 13.5	0.963 ± 0.044	0.4 ± 0.4	1.8 ± 2.4
5	18.4 ± 3.4	24 ± 12.4	0.968 ± 0.035	0.5 ± 0.4	1.8 ± 2.4
6	17.2 ± 4.6	22.3 ± 11.6	0.971 ± 0.03	0.4 ± 0.4	1.2 ± 1.3
7	14.7 ± 4.7	18.9 ± 9.8	0.980 ± 0.019	0.3 ± 0.3	0.8 ± 0.6
8	12.9 ± 3.7	17.6 ± 8.6	0.984 ± 0.015	0.2 ± 0.2	0.6 ± 0.7
9	11.8 ± 3.6	16.2 ± 8.3	0.985 ± 0.016	0.2 ± 0.2	0.6 ± 0.7
12	8.3 ± 2.5	11.2 ± 6.3	0.993 ± 0.009	0.1 ± 0.2	0.6 ± 0.6
15	6.0 ± 1.9	7.6 ± 3.7	0.997 ± 0.004	0.1 ± 0.1	0.6 ± 0.6
18	4.6 ± 1.1	6.0 ± 2.8	0.998 ± 0.002	0.0 ± 0.0	0.5 ± 0.5
21	3.5 ± 0.8	4.5 ± 2.3	0.999 ± 0.001	0.0 ± 0.0	0.4 ± 0.5

## Data Availability

The data presented in this study are available on request from the corresponding author. The data are not publicly available due to the nonstandard file formats.

## Supplementary Materials

The following supporting information can be downloaded at: https://www.mdpi.com/article/10.3390/s25092706/s1, S.1: Comparing SSA for different protocols. S.1.1: Optimal locations for the OPM-2AX system using protocol I. S.1.2: Optimal locations for the OPM-2AX system using protocol II. S.1.3: Optimal locations for the OPM-2AX system using protocol III. S.1.4: Optimal locations for the OPM-2AX system using protocol I. S.1.5: Optimal locations for the SQUID system. S.1.6: Optimal locations for the OPM-2AX system sensors layout covering right hemisphere only. S.2: Localizations of M100 and M50 for all measurements. S.3: Localizations of M100 and M50 for all measurements using data from the right hemisphere only.

## Abbreviations

The following abbreviations are used in this manuscript:

MDPI	Multidisciplinary Digital Publishing Institute
MEG	Magnetoencephalography
SQUID	Superconducting quantum interference device
OPM	Optically pumped magnetometer
MFM	Magnetic field map
ROI	Region of interest
SSA	Sequential selection algorithm
AEF	Auditory evoked field
MNE	Minimum norm estimation
ECD	Equivalent current dipole
CC	Correlation coefficient
RMS	Root mean square
MSR	Magnetically shielded room
MRI	Magnetic resonance imaging
ECG	Electrocardiography
SNR	Signal-to-noise ratio
BEM	Boundary element method

## Appendix A

### Appendix A.1. Theoretical Background of Meg Forward Modeling of Sources Inside a Multi-Layer Shell Model

The total electrical current within the brain resulting from neuronal activity is divided into primary $\vec{J_{p}}$ and secondary (volume) currents $\vec{J_{s}}$. Primary currents represent ionic currents passing through the cell membrane, while secondary currents are extracellular currents not described by primary currents. Primary currents give rise to a macroscopic electric field $\vec{E}(\vec{r})$, and secondary currents ($\vec{J_{s}}(\vec{r})$) arise due to Ohm’s law in tissue with macroscopic conductivity $\sigma (\vec{r})$. By considering a quasi-static approximation, we can express the electric field as a gradient of electric potential with a negative sign $\left(\vec{E}=-\nabla V\right)$. For a chosen location within the brain ($\vec{r}$), we can write the total electric current as:

(A1) $$\vec{J}(\vec{r})=\vec{J_{p}}(\vec{r})+\vec{J_{s}}(\vec{r})=\vec{J_{p}}(\vec{r})+\sigma (\vec{r})\vec{E}(\vec{r})=\vec{J_{p}}(\vec{r})-\sigma (\vec{r})\nabla V(\vec{r}).$$

The forward model is the mathematical formulation used to calculate the magnetic field and electric potential for a known electric current. In general, to calculate the magnetic field density ($\vec{B}$) at a desired location $\vec{r}$, we use the Biot–Savart law, which is derived from Maxwell’s equations

(A2) $$\vec{B}\left(\vec{r}\right)=\frac{\mu_{0}}{4\pi }\int \vec{J}\left({\vec{r}}^{\prime }\right)\times \frac{\vec{r}-\vec{r}\prime }{{\left|\vec{r}-\vec{r}\prime \right|}^{3}}dv^{\prime },$$

where $\mu_{0}$ is the induction constant, $\vec{r}\prime$ is the local vector to the sources $\vec{J}(\vec{r}\prime )$ within the volume v′ over which we integrate. First, we need to calculate the current $\vec{J}$. By considering Equation (A1) and the continuity equation, which is with the quasi-static approximation equal to $\nabla \cdot \vec{J}=0$, we derive the electric potential as a result of the primary current:

(A3) $$\nabla \cdot (\sigma \nabla V)=\nabla \cdot \vec{J_{p}}$$

This relation (the electrical problem) is not generally analytically solvable. The simplest analytical solution assumes that we have a source in an infinite homogeneous space (σ=const.).

A very commonly used analytical solution is for the case of magnetic field density ($\vec{B}$) outside a homogeneous spherical conductor generated by a current dipole $\vec{p}$ at a point $\vec{r_{p}}$ in the interior. The equation was derived by Sarvas [36]:

(A4) $$\vec{B}\left(\vec{r}\right)=\frac{\mu_{0}}{4\pi F^{2}}\left(F\left(\vec{p}\times \vec{r_{p}}\right)-\left(\vec{p}\times \vec{r_{p}}\cdot \vec{r}\right)\nabla F\right),$$

(A5) $$F=\left|\vec{a}\right|\left(\left|\vec{r}\right|\left|\vec{a}\right|+\vec{a}\cdot \vec{r}\right),$$

(A6) $$\nabla F=\left(\frac{F}{{\left|\vec{a}\right|}^{2}}+\left|\vec{a}\right|+\left|\vec{r}\right|\right)\vec{a}+\left(\frac{{\left|\vec{a}\right|}^{2}}{\left|\vec{r}\right|}+\left|\vec{a}\right|\right)\vec{r},$$

where $\vec{a}=\vec{r}–{\vec{r}}_{p}$.

Next, we present the forward model that assumes that the current sources are inside a volume conductor with several homogeneous layers $\;G_{i}$ (i=1,…,m) with an outer surface $\;S_{i}$. Each i-th layer has a constant conductivity ($\sigma_{i})$. $\vec{B}$ in this case, can be calculated at the location $\vec{r}$ using the formula derived by Geselowitz:

(A7) $$\vec{B}(\vec{r})=\vec{B_{0}}(\vec{r})-\frac{\mu_{0}}{4\pi }\sum_{i=1}^{m}({\sigma_{i}}^{-}-{\sigma_{i}}^{+})\int_{S_{i}}V(\vec{r}\prime )\vec{n}(\vec{r}\prime )\times \frac{\vec{r}-\vec{r}\prime }{{\left|\vec{r}-\vec{r}\prime \right|}^{3}}dS\prime$$

where $\vec{n}(\vec{r}\prime )$ is the normal to the boundary surface S′; $\vec{r}\prime$ is the local vector of the i-th surface $S_{i}$ and V is the electric potential on this surface. ${\sigma_{i}}^{-}$ and ${\sigma_{i}}^{+}$ are the conductivities on the inside and outside of $S_{i}$. $\vec{B_{0}}(\vec{r})$ is the magnetic field density for the primary current $\vec{J_{p}}$ in the infinite homogeneous medium:

(A8) $${\vec{B}}_{0}\left(\vec{r}\right)=\frac{\mu_{0}}{4\pi }\int_{V}{\vec{J}}_{p}\left(\vec{r\prime }\right)\frac{\left(\vec{r}-\vec{r\prime }\right)}{{\left|\vec{r}-\vec{r\prime }\right|}^{3}}d\vec{V\prime }$$

In this case, the electrical problem cannot be solved analytically. The BEM method is the most suitable method for the calculation, and the detailed solution is clearly described in [60]. We calculate the potential V at the boundary surfaces between the regions of different conductivities (A3). For a single homogeneous layer, we assume a constant σ, and we consider two boundary conditions. At the boundary of two layers, both the electric potential ($V^{-}=V^{+}$) and the electric current in the direction normal ($\vec{n}$) to the boundary surface S $\left(\sigma^{-}\partial \frac{V^{-}}{\partial \vec{n}}=\sigma^{+}\partial \frac{V^{+}}{\partial \vec{n}}\right)$, are continuous.

### Appendix A.2. Theoretical Background of the Minimum Norm Estimate (MNE) Method

The inverse problem in MEG can be solved with several approaches, here we present a solution using the minimum norm method (MNE). The methodology described below is adapted from [36]. First, we must define a source space for which we want to reconstruct the brain’s activity. Typically, we distribute these sources over the brain’s surface, m denotes the number of sources and n the number of sensors. For each i-th sensor, we can write the m-array of transfer functions as:

(A9) $$L_{i}=\left({\vec{L}}_{i}\left({\vec{r}}_{p_{1}}\right),{\vec{L}}_{i}\left({\vec{r}}_{p_{2}}\right),\ldots ,{\vec{L}}_{i}\left({\vec{r}}_{p_{m}}\right)\right),$$

where the value of the transfer function of j-th source (j=1,2,…,m) depends on the location of the source (${\vec{L}}_{i}({\vec{r}}_{p_{j}})$). We can write the amplitudes and directions of all the sources (primary current) in the form of a m-array of vectors:

(A10) $$P={\left({\vec{p}}_{1},{\vec{p}}_{2}\ldots ,{\vec{p}}_{m}\right)}^{T}.$$

The measured magnetic field of i-th sensor with sensing direction ${\vec{e}}_{i}$ is then

(A11) $$B_{i}={\vec{B}}_{i}\cdot {\vec{e}}_{i}=\sum_{j}{\vec{L}}_{i}\left({\vec{r}}_{p_{j}}\right)\cdot {\vec{p}}_{j}=L_{i}P.$$

We are searching for sources $\vec{p}*$, which explain our measurement and are of shapes:

(A12) $${\vec{p}}^{*}=\sum_{i=1}^{n}w_{i}{\vec{L}}_{i}\left({\vec{r}}_{p_{j}}\right),$$

where $\vec{p}*$ is an element of the space of all possible primary current sources. The weight is defined as the $w=(w_{1},w_{2},\ldots ,w_{n})$. We write the individual elements of the transfer function matrix as:

(A13) $$\Gamma_{kl}=L_{k}\cdot L_{l}.$$

Combining Equations (A11)–(A13), we obtain the following expression:

(A14) $$B_{i}=\sum_{l=1}^{n}\Gamma_{il}w_{l},$$

which we can rewrite as:

(A15) $$w=\Gamma^{-1}B,$$

under the assumption that there is a solution $\Gamma^{-1}$. Often the solution $\Gamma^{-1}$ is not well conditioned since the vectors of the transfer function $L_{i}$ may be linearly dependent on each other. Therefore, the matrix Γ can contain very small values, which can cause an error in calculating w. Consequently, we regularize the matrix Γ by decomposing it into eigenvalues $\Gamma =U\Lambda V^{T}$. Where Λ is a diagonal matrix with diagonal elements $\Lambda =(\lambda_{1},\lambda_{2},\ldots ,\lambda_{n})$. The inverse of the decomposed and regularized matrix Γ is:

(A16) $${\tilde{\Gamma }}^{-1}=V{\tilde{\Lambda }}^{-1}U^{T},$$

with diagonal elements of the matrix ${\tilde{\Lambda }}^{-1}=(\lambda_{1}^{-1},\lambda_{2}^{-1},\ldots ,\lambda_{K}^{-1},0,\ldots ,0)$. The index  K is determined by the magnitude of the noise in the measurements. The regularized solution (amplitudes and directions of all sources) is $\tilde{P}={\left({\tilde{\Gamma }}^{-1}B\right)}^{T}L$

### Appendix A.3. Implementation Details of the System Transformation Using MNE and BEM from the Software Package Mne-Python

In our work, we want to investigate how many sensors are needed in an OPM-MEG system to capture the most information. We used the SSA algorithm to select the optimal sites and calculate the information density. As we do not currently have measurements with more than 20 OPM sensors, in this work, we used measurements from SQUID systems with 125 sensors, and we transformed these measurements to a hypothetical OPM-MEG system, which covers the whole head (80 sensors). Here, we present the implementation details of the transformation method we used in our work.

The transformation between two systems works as follows: first, we calculated the source reconstruction with the first system (from which we want to transform the data). We calculated the magnetic fields on the second system with the reconstructed source model and a forward model. For this calculation, both systems must be in the same coordinate system as the source model. Our data transformation in this work uses the MNE method (Appendix A.2) to solve the inverse problem and a direct model, which assumes sources inside a multi-layer shell based on individual anatomical information (Appendix A.1). For the computation of the forward model and inverse problem, we used the implementations within the MNE-Python software package [31,32]. All the implementation details of individual functions from the MNE-Python are stated on the website https://mne.tools (accessed on 24 February 2025). When calculating the forward model, we considered the realistic sensor geometry of the QZFM Gen-2 sensor (QuSpin, Inc., Louisville, CO, USA). The magnetic field was calculated by integrating over 8 points within the sensor gas cell (cube nodes) with equal weights. We used MRI head measurements to incorporate the subjects’ individual anatomical data. The MRIs were segmented using the FreeSurfer software (command “recon-all”) to obtain individual layers of the head and brain (scalp, skull, white matter, etc.) [61]. These reconstructed layers are used in combination with MNE-Python functions. The generated source space is distributed over the gray matter surface for both hemispheres. Each hemisphere has approximately 4098 sources. We used the command “mne.setup_source_space()” to generate the source space object.

Next, we must create the forward model. First, we created surfaces suitable for calculation with the BEM method. We used the function “mne.bem.watershed_bem”; this creates triangulated surfaces of the brain, inner skull, outer skull, and scalp. Next, we made the BEM model with the “mne.make_bem_model()” function. Our BEM model contains three surfaces: the outer scalp, the outer skull, and the brain surface. Each surface consists of 5120 triangles with a total of 2562 nodes. The software default conductivities were used: for the scalp and brain region, a conductivity of 0.3 S/m was used. The conductivity of the skull is much smaller, with a value of 0.006 S/m. We used the function “mne.make_bem_solution()” to calculate the BEM solution, which uses the linear collocation approach. To calculate the forward solution for our generated source space and for both systems (OPM-MEG and SQUID-MEG), we used the function “mne.make_forward_solution()”.

To calculate the inverse solution with the MNE method, we first created the inverse operator with the function “mne.minimum_norm.make_inverse_operator()”. We used the parameter “fixed=True”, which fixes the source orientations of the source space normal to the cortical mantle. Additionally, we included the covariance noise matrix for the SQUID-MEG measurements. To create this matrix, we used the data of the prestimulation time window (−0.2 s to 0.0 s relative to auditory stimulation). To calculate the source estimates (inverse solution) for the whole time interval of the evoked signal (−0.2 s to 0.5 s relative to auditory stimulation), we used the function “mne.minimum_norm.apply_inverse()”. We set the parameters to “method= ‘MNE’” and “lambda2 = 1/9”; the latter represents the regularization parameter. To calculate and transform the data on the hypothetic OPM-MEG system, we used the calculated source estimates and the forward model with the function “mne.apply_forward()”.
