# Supplementary material for: Optimization of OPM-MEG Layouts with a Limited Number of Sensors
Source: Sensors (Basel). 2025 Apr 24;25(9):2706. doi: 10.3390/s25092706 (PMC12074169; doi:10.3390/s25092706)
Supplement: Supplementary file 1 [file sensors-25-02706-s001.zip › sensors-3558196-supplementary.pdf]

S Supplementary Materials

Optimization of OPM-MEG layouts with a limited number of sensors

Urban Marhl, Rok Hren, Tilmann Sander, and Vojko Jazbinšek

Contents

S.1 Comparing SSA for different protocols . . . 3
S.1.1 Optimal locations for the OPM-2AX system using protocol I. . . . 3
S.1.2 Optimal locations for the OPM-2AX system using protocol II. . . . 4
S.1.3 Optimal locations for the OPM-2AX system using protocol III. . . . 5
S.1.4 Optimal locations for the OPM-2AX system using protocol IV. . . . 6
S.1.5 Optimal locations for the SQUID system. . . . 8
S.1.6 Optimal locations for the OPM-2AX system sensors' layout covering right hemisphere only. . . . 9
S.2 Localization of M100 and M50 for all measurements . . . 10
S.3 Localization of M100 and M50 for all measurements using data from the right hemisphere only . . . 26

List of Figures

S.1 Selection of 30 channels for OPM-2AX on time interval 42 – 240 ms, protocol I. **I** . . . 3
S.2 SSA protocol **I**, evaluation measures RMS, RD and CC. . . . 3
S.3 Selection of 30 measuring sites for OPM-2AX on time interval 42 – 240 ms, protocol **II** . . . 4
S.4 SSA protocol **II**, evaluation measures RMS, RD and CC. . . . 4
S.5 Selection of 30 measuring sites for OPM-2AX on time interval 42 – 240 ms, protocol **III** . . . 5
S.6 SSA protocol **III**, evaluation measures RMS, RD and CC. . . . 5
S.7 Selection of 30 measuring sites for OPM-2AX on time interval 42 – 240 ms, protocol **IV** . . . 6
S.8 SSA protocol **IV**, evaluation measures RMS, RD and CC. . . . 6
S.9 Selection with protocol **IV** – recalculation with pairs of channels on each selected site. . . . 7
S.10 SSA protocol **IV**, evaluation with recalculation using pairs of channels on each selected site. . . . 7
S.11 Selection of 30 measuring sites for SQUID-MEG on time interval 42 – 240 ms. . . . 8
S.12 SSA on SQUID data, evaluation measures RMS, RD and CC. . . . 8
S.13 Selection of 21 measuring sites on the right hemisphere for OPM-2AX on time interval 42 – 240 ms, protocol **III** 9
S.14 SSA protocol **III**, right hemisphere only, evaluation measures RMS, RD and CC. . . . 9
S.15 Subject-1f1: 18 selected sites using protocol **III**, fitting M100 with 2 dipoles . . . 10
S.16 Subject-1f1: 18 selected sites using protocol **III**, fitting M50 with 2 dipoles . . . 10
S.17 Subject-1f2: 18 selected sites using protocol **III**, fitting M100 with 1 dipole . . . 11
S.18 Subject-1f2: 18 selected sites using protocol **III**, fitting M50 with 2 dipoles . . . 11
S.19 Subject-2m1: 18 selected sites using protocol **III**, fitting M100 with 2 dipoles . . . 12
S.20 Subject-2m1: 18 selected sites using protocol **III**, fitting M50 with 2 dipoles . . . 12
S.21 Subject-3f1: 18 selected sites using protocol **III**, fitting M100 with 2 dipoles . . . 13
S.22 Subject-3f1: 18 selected sites using protocol **III**, fitting M50 with 2 dipoles . . . 13
S.23 Subject-3f2: 18 selected sites using protocol **III**, fitting M100 with 2 dipoles . . . 14
S.24 Subject-3f2: 18 selected sites using protocol **III**, fitting M50 with 2 dipoles . . . 14
S.25 Subject-4m1: 18 selected sites using protocol **III**, fitting M100 with 2 dipoles . . . 15
S.26 Subject-4m1: 18 selected sites using protocol **III**, fitting M50 with 2 dipoles . . . 15
S.27 Subject-4m2: 18 selected sites using protocol **III**, fitting M100 with 2 dipoles . . . 16
S.28 Subject-4m2: 18 selected sites using protocol **III**, fitting M50 with 2 dipoles . . . 16
S.29 Subject-5m1: 18 selected sites using protocol **III**, fitting M100 with 2 dipoles . . . 17
S.30 Subject-5m1: 18 selected sites using protocol **III**, fitting M50 with 2 dipoles . . . 17
S.31 Subject-6f1: 18 selected sites using protocol **III**, fitting M100 with 2 dipoles . . . 18
S.32 Subject-6f1: 18 selected sites using protocol **III**, fitting M50 with 2 dipoles . . . 18
S.33 Subject-6f2: 18 selected sites using protocol **III**, fitting M100 with 2 dipoles . . . 19
S.34 Subject-6f2: 18 selected sites using protocol **III**, fitting M50 with 2 dipoles . . . 19
S.35 Subject-7m1: 18 selected sites using protocol **III**, fitting M100 with 2 dipoles . . . 20
S.36 Subject-7m1: 18 selected sites using protocol **III**, fitting M50 with 2 dipoles . . . 20
S.37 Subject-7m2: 18 selected sites using protocol **III**, fitting M100 with 2 dipoles . . . 21
S.38 Subject-7m2: 18 selected sites using protocol **III**, fitting M50 with 1 dipole . . . 21
S.39 Subject-8m1: 18 selected sites using protocol **III**, fitting M100 with 2 dipoles . . . 22
S.40 Subject-8m1: 18 selected sites using protocol **III**, fitting M50 with 2 dipoles . . . 22
S.41 Subject-8m2: 18 selected sites using protocol **III**, fitting M100 with 2 dipoles . . . 23
S.42 Subject-8m2: 18 selected sites using protocol **III**, fitting M50 with 2 dipoles . . . 23
S.43 Subject-9m1: 18 selected sites using protocol **III**, fitting M100 with 2 dipoles . . . 24

S.44 Subject-9m1: 18 selected sites using protocol **III**, fitting M50 with 2 dipoles 24
S.45 Subject-9m2: 36 selected channels using protocol **III**, fitting M100 with 1 dipole 25
S.46 Subject-9m2: 18 selected sites using protocol **III**, fitting M50 with 2 dipoles 25
S.47 Subject-1f1: 18 selected channels using protocol **III** on right hemisphere only, fitting M100 with 1 dipole 26
S.48 Subject-1f1: 18 selected channels using protocol **III** on right hemisphere only, fitting M50 with 1 dipole 26
S.49 Subject-1f2: 18 selected channels using protocol **III** on right hemisphere only, fitting M100 with 1 dipole 27
S.50 Subject-1f2: 18 selected channels using protocol **III** on right hemisphere only, fitting M50 with 1 dipole 27
S.51 Subject-2m1: 18 selected channels using protocol **III** on right hemisphere only, fitting M100 with 1 dipole 28
S.52 Subject-2m1: 18 selected channels using protocol **III** on right hemisphere only, fitting M50 with 1 dipole 28
S.53 Subject-3f1: 18 selected channels using protocol **III** on right hemisphere only, fitting M100 with 1 dipole 29
S.54 Subject-3f1: 18 selected channels using protocol **III** on right hemisphere only, fitting M50 with 1 dipole 29
S.55 Subject-3f2: 18 selected channels using protocol **III** on right hemisphere only, fitting M100 with 1 dipole 30
S.56 Subject-3f2: 18 selected channels using protocol **III** on right hemisphere only, fitting M50 with 1 dipole 30
S.57 Subject-4m1: 18 selected channels using protocol **III** on right hemisphere only, fitting M100 with 1 dipole 31
S.58 Subject-4m1: 18 selected channels using protocol **III** on right hemisphere only, fitting M50 with 1 dipole 31
S.59 Subject-4m2: 18 selected channels using protocol **III** on right hemisphere only, fitting M100 with 1 dipole 32
S.60 Subject-4m2: 18 selected channels using protocol **III** on right hemisphere only, fitting M50 with 1 dipole 32
S.61 Subject-5m1: 18 selected channels using protocol **III** on right hemisphere only, fitting M100 with 1 dipole 33
S.62 Subject-5m1: 18 selected channels using protocol **III** on right hemisphere only, fitting M50 with 1 dipole 33
S.63 Subject-6f1: 18 selected channels using protocol **III** on right hemisphere only, fitting M100 with 1 dipole 34
S.64 Subject-6f1: 18 selected channels using protocol **III** on right hemisphere only, fitting M50 with 1 dipole 34
S.65 Subject-6f2: 18 selected channels using protocol **III** on right hemisphere only, fitting M100 with 1 dipole 35
S.66 Subject-6f2: 18 selected channels using protocol **III** on right hemisphere only, fitting M50 with 1 dipole 35
S.67 Subject-7m1: 18 selected channels using protocol **III** on right hemisphere only, fitting M100 with 1 dipole 36
S.68 Subject-7m1: 9 selected sites using protocol **III** on right hemisphere only, fitting M50 with 1 dipole 36
S.69 Subject-7m2: 18 selected channels using protocol **III** on right hemisphere only, fitting M100 with 1 dipole 37
S.70 Subject-7m2: 18 selected channels using protocol **III** on right hemisphere only, fitting M50 with 1 dipole 37
S.71 Subject-8m1: 18 selected channels using protocol **III** on right hemisphere only, fitting M100 with 1 dipole 38
S.72 Subject-8m1: 18 selected channels using protocol **III** on right hemisphere only, fitting M50 with 1 dipole 38
S.73 Subject-8m2: 18 selected channels using protocol **III** on right hemisphere only, fitting M100 with 1 dipole 39
S.74 Subject-8m2: 18 selected channels using protocol **III** on right hemisphere only, fitting M50 with 1 dipole 39
S.75 Subject-9m1: 18 selected channels using protocol **III** on right hemisphere only, fitting M100 with 1 dipole 40
S.76 Subject-9m1: 18 selected channels using protocol **III** on right hemisphere only, fitting M50 with 1 dipole 40
S.77 Subject-9m2: 18 selected channels using protocol **III** on right hemisphere only, fitting M100 with 1 dipole 41
S.78 Subject-9m2: 18 selected channels using protocol **III** on right hemisphere only, fitting M50 with 1 dipole 41

S.1 Comparing SSA for different protocols

S.1.1 Optimal locations for the OPM-2AX system using protocol I.

Protocol I. We treat the radial and tangential channels as independent, giving 160 channels, 80 radial and 80 tangential. This approach selects N_s channels using the SSA algorithm.

Figure S.1a shows the first 30 selected channels using protocol **I**. There are 29 out od 30 radial channels selected, and only one tangential. This is somehow expected, since the SSA optimizes $\mathrm{RMS_{err}}$, which are quite higher for OPM-RAD compared to OPM-TAN (see, Fig. 3a in the main paper). Figure S.1b shows relative statistical power vs. number of selected channels. Figure S.2 displays results of evaluation on different time interval.

For comparison, the CC at learning time interval of [42,240] ms from figure S.1b is also shown S.1c. Relative statistical power exceeds values of 0.90 and 0.95 after 9th and 15th optimally selected channels.

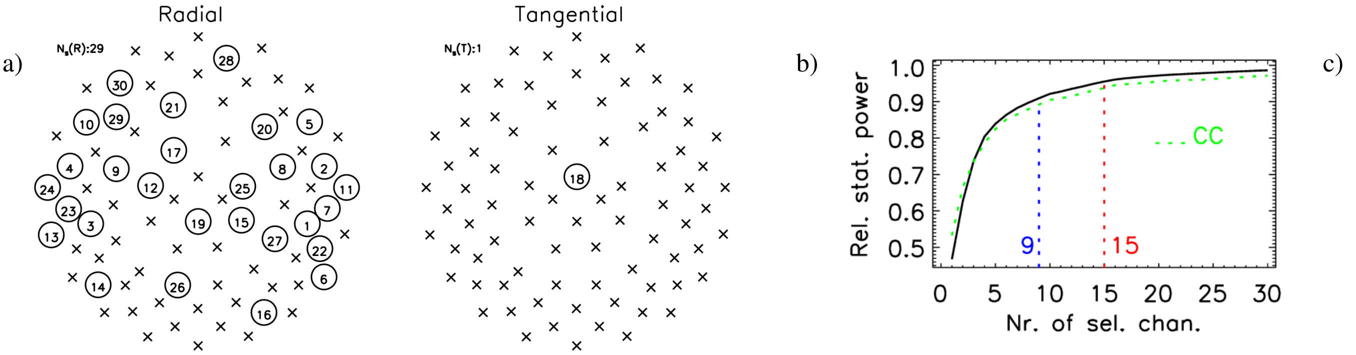

Fig. S.1: Selection of 30 channels for OPM-2AX on time interval 42 – 240 ms, protocol I. **I**: a) OPM grid, encircled numbers show the order of selected channels, crosses denotes unselected channels, b) relative statistical power vs. number of selected channels.

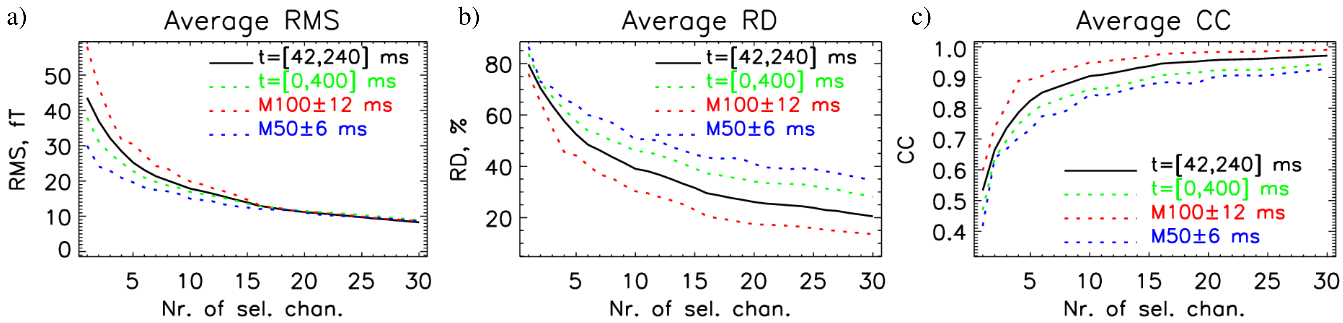

Fig. S.2: SSA protocol **I**, evaluation measures RMS, RD and CC.

N_s=12	[0,400] ms	[42,240] ms	M100±12 ms	M50±6 ms
RMS	15.1±4.4	15.8±4.4	17.2±4.6	13.7±6.2
RD	38.6±18.3	31.9±14.8	24.5±11.1	43±16
CC	0.901±0.114	0.936±0.075	0.966±0.033	0.888±0.087

N_s=18	[0,400] ms	[42,240] ms	M100±12 ms	M50±6 ms
RMS	11.5±3	11.3±2.6	11.5±2.5	11.2±5.2
RD	30.4±16.6	23.5±12.1	16.3±6.4	36.3±15.8
CC	0.937±0.083	0.964±0.047	0.985±0.012	0.918±0.076

N_s=24	[0,400] ms	[42,240] ms	M100±12 ms	M50±6 ms
RMS	9.8±2.4	9.3±1.9	9.5±2.1	9.2±2.9
RD	26.6±15.6	19.7±11.1	13.4±5.1	30.9±13.1
CC	0.951±0.07	0.974±0.039	0.99±0.008	0.943±0.049

N_s=30	[0,400] ms	[42,240] ms	M100±12 ms	M50±6 ms
RMS	8.1±2.2	7.5±1.5	7.8±2.2	7.7±1.8
RD	21.9±13.2	16±9.7	10.8±3.9	26.3±10.9
CC	0.967±0.046	0.982±0.029	0.994±0.005	0.959±0.035

S.1.2 Optimal locations for the OPM-2AX system using protocol II.

Protocol II. During the SSA selection, we chose channels (same as with approach **I**.). Finally, we add the channel pairs (exact location, different measuring component) that have not been yet selected. This gives N_m of selected measurement sites and $N_s = 2N_m$ channels.

Figure S.3a shows the first 30 selected sites using protocol **II**. Figure S.4 displays results of evaluation on different time interval.

For comparison, the CC at learning time interval of [42,240] ms from figure S.3b is also shown S.3c. Relative statistical power exceeds values of 0.90 and 0.95 after 6th and 9th optimally selected sites.

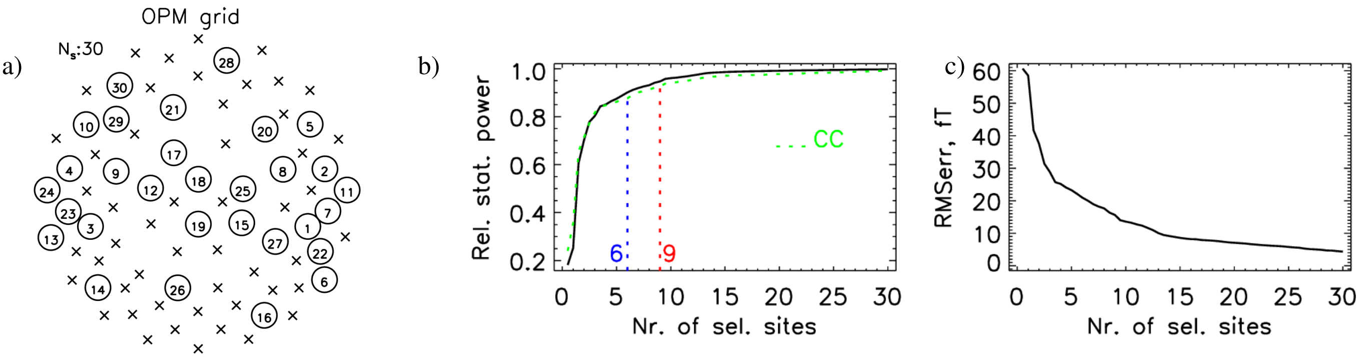

Fig. S.3: Selection of 30 measuring sites for OPM-2AX on time interval 42 – 240 ms, protocol **II**: a) OPM grid, encircled numbers show the order of selected sites, crosses denotes unselected sites, b) relative statistical power, and c) RMSerr vs. number of selected sites.

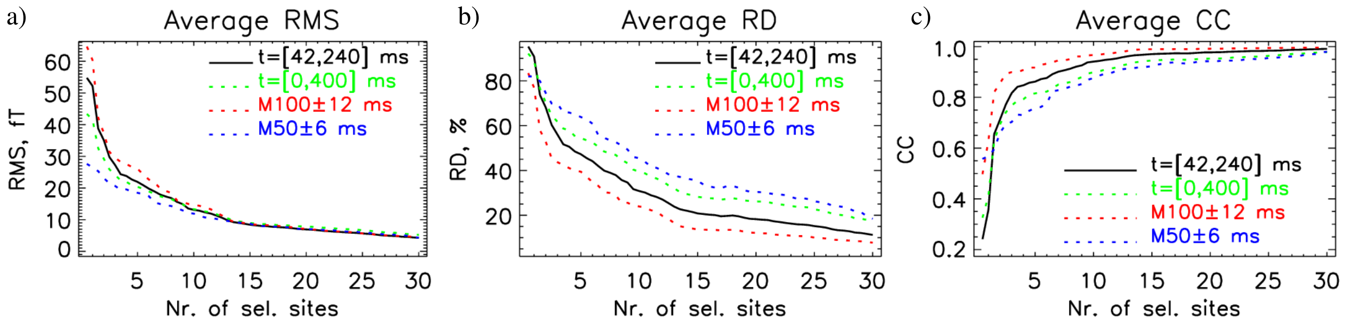

Fig. S.4: SSA protocol **II**, evaluation measures RMS, RD and CC.

N_m=12	[0,400] ms	[42,240] ms	M100±12 ms	M50±6 ms
RMS	18.2±6.6	19±6.6	21.8±7.2	16.7±8
RD	45.8±21.7	38.8±19.7	30.8±14.1	52.4±19.6
CC	0.865±0.142	0.904±0.112	0.946±0.051	0.835±0.13

N_m=18	[0,400] ms	[42,240] ms	M100±12 ms	M50±6 ms
RMS	14±4.6	14±4.4	14.7±3.9	12.2±5.2
RD	35.8±17.6	28.6±14.1	21.1±9.8	40.3±17.3
CC	0.916±0.096	0.949±0.06	0.973±0.028	0.902±0.079

N_m=24	[0,400] ms	[42,240] ms	M100±12 ms	M50±6 ms
RMS	10.8±3.3	10.4±2.6	11.2±3.5	9.4±2.5
RD	28.4±15.3	21.7±11.3	15.9±7.6	31.6±13
CC	0.946±0.065	0.97±0.038	0.984±0.019	0.941±0.052

N_m=30	[0,400] ms	[42,240] ms	M100±12 ms	M50±6 ms
RMS	8.1±2.1	7.6±1.5	7.8±2.2	7.9±1.9
RD	21.9±13.2	16.2±9.9	10.8±4.1	27.2±12.5
CC	0.967±0.046	0.982±0.03	0.993±0.005	0.955±0.044

S.1.3 Optimal locations for the OPM-2AX system using protocol III.

Protocol III. This approach is a combination of approaches **I** and **II**. When we select one channel (radial or tangential) during the SSA, we also choose the channel pair. Similarly, as **II** this gives N_m of selected measurement sites and $N_s = 2N_m$ channels.

Figure S.5a shows the first 30 selected sites using protocol **III**. Figure S.6 displays results of evaluation on different time interval.

For comparison, the CC at learning time interval of [42,240] ms from figure S.5b is also shown S.5c. Relative statistical power exceeds values of 0.90 and 0.95 after 4th and 7th optimally selected sites.

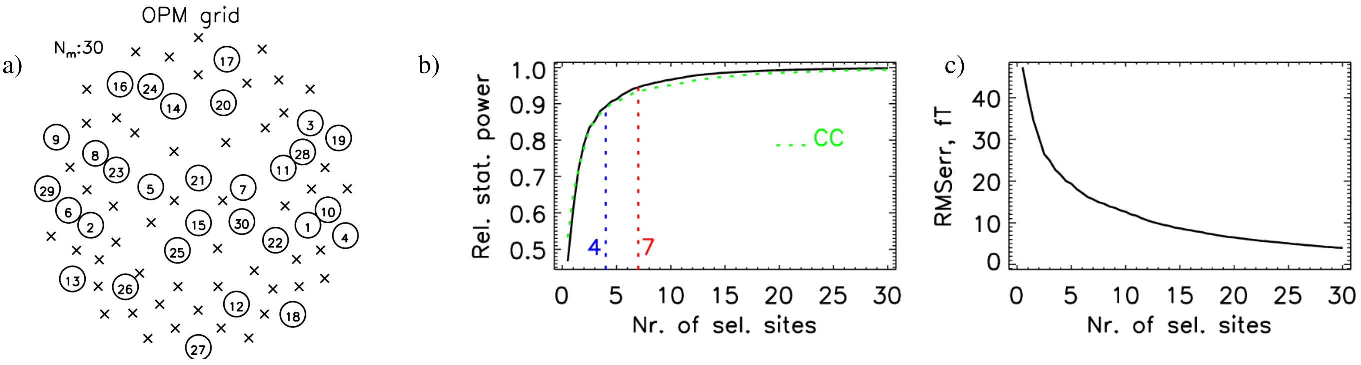

Fig. S.5: Selection of 30 measuring sites for OPM-2AX on time interval 42 – 240 ms, protocol **III**: a) OPM grid, encircled numbers show the order of selected sites, crosses denotes unselected sites, b) relative statistical power, and c) RMSerr vs. number of selected sites.

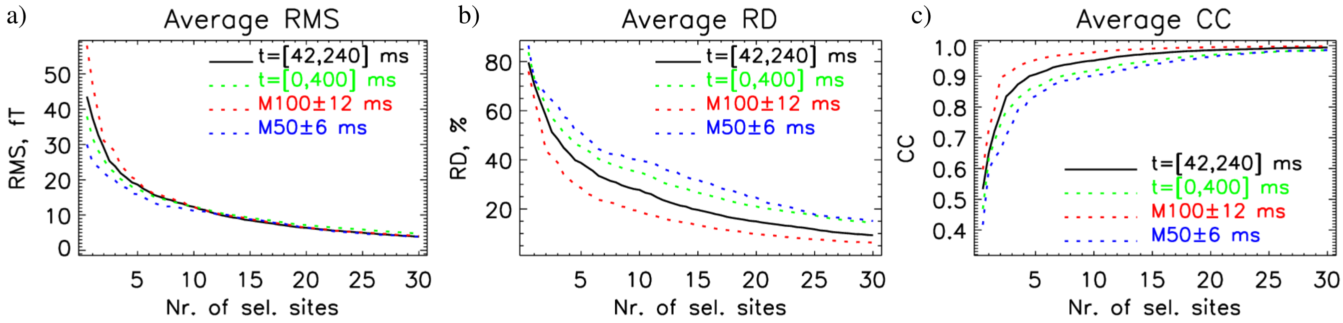

Fig. S.6: SSA protocol **III**, evaluation measures RMS, RD and CC.

N_m=12	[0,400] ms	[42,240] ms	M100±12 ms	M50±6 ms
RMS	15.4±4.2	16±4.2	16.2±3.9	13.6±6.9
RD	39.4±18.6	32.7±15.3	23.2±10.3	44±18.4
CC	0.897±0.115	0.932±0.076	0.968±0.031	0.875±0.108

N_m=18	[0,400] ms	[42,240] ms	M100±12 ms	M50±6 ms
RMS	12.4±3.2	12.4±3.1	13±3	11.2±3.9
RD	32.5±16.8	25.8±13.1	18.4±7.9	37.3±16.2
CC	0.929±0.087	0.957±0.055	0.98±0.02	0.914±0.077

N_m=24	[0,400] ms	[42,240] ms	M100±12 ms	M50±6 ms
RMS	10±2.7	9.5±2.2	9.7±2.4	9.5±3.7
RD	26.5±14.9	20.1±11.1	13.7±5.3	31.7±13.6
CC	0.952±0.065	0.973±0.039	0.989±0.009	0.939±0.053

N_m=30	[0,400] ms	[42,240] ms	M100±12 ms	M50±6 ms
RMS	8.3±2.1	7.7±1.5	7.9±1.8	7.9±2.4
RD	22.6±13.6	16.4±9.8	11.1±3.8	27.2±12.8
CC	0.964±0.051	0.981±0.03	0.993±0.005	0.954±0.046

S.1.4 Optimal locations for the OPM-2AX system using protocol IV.

Protocol IV. With this approach, we combine the radial and tangential MFMs into a common basis with twice the number of all MFMs. We consider the measurement system to be 80-channel. The transfer matrix (T) in equation (5) is the same for radial and tangential channels.

Figure S.7a shows the first 30 selected sites using protocol **IV**. Figure S.8 displays results of evaluation on different time interval.

For comparison, the CC at learning time interval of [42,240] ms from figure S.7b is also shown S.7c. Relative statistical power exceeds values of 0.90 and 0.95 after 12th and 18th optimally selected sites.

Results for time intervals around M50 and M100 are much worse compared to other three approaches, what is expected since we have the same transfer matrix for radial an tangential channels.

How the selected sites from S.7a performs if we put pairs of channels in each selected site is demonstrated in Figs. S.9 and S.10. In this case, the relative statistical power exceeds values of 0.90 and 0.95 after 7th and 12th optimally selected sites.

The main difference between protocols **II**, **III**, and **IV** (recalculated) is in the selection of the first 10–15 measuring sites, afterwards average RMS_{err}, RD and CC are similar. These results corroborate the finding in Lux et al. that the "optimal" lead selection is non-unique, i.e., that slightly shifted position of the first couple of leads could generate quite different lead sets, which perform equally well.

The best results for the first 10–15 selected sites we obtained by protocol **III**, where the relative statistical power exceeds 0.90 and 0.95 after only 4th and 7th "optimal" selection, respectively. Therefore, we used protocol **III** as a default approach, and all the results in the main paper are obtained with this protocol.

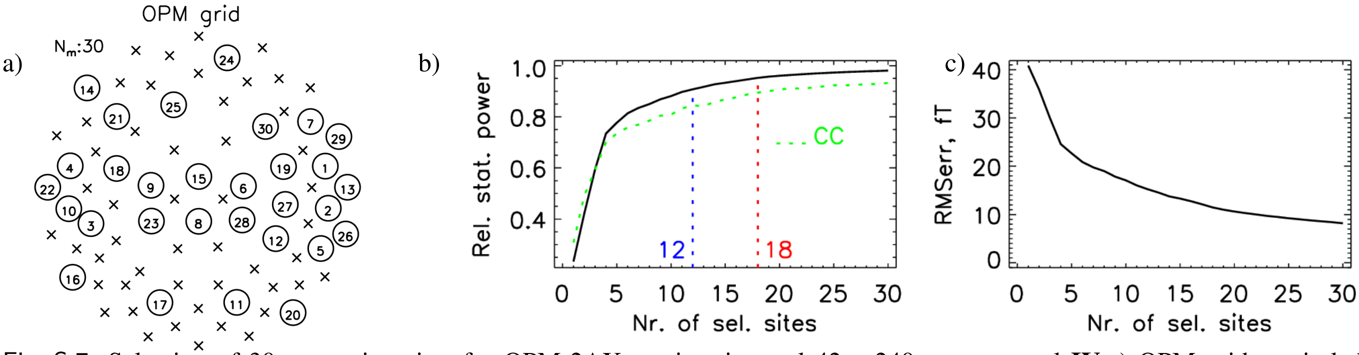

Fig. S.7: Selection of 30 measuring sites for OPM-2AX on time interval 42 – 240 ms, protocol **IV**:a) OPM grid, encircled numbers show the order of selected channels, crosses denotes unselected channels, b) relative statistical power, and c) RMSerr vs. number of selected channels.

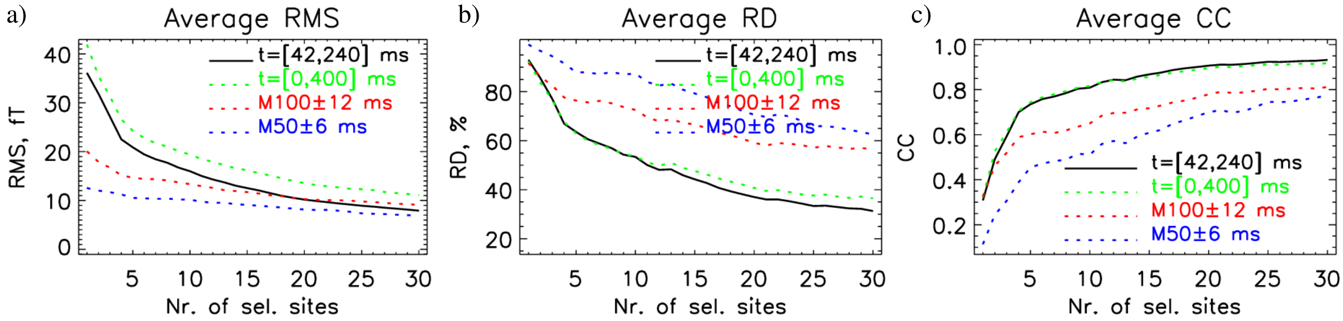

Fig. S.8: SSA protocol **IV**, evaluation measures RMS, RD and CC.

N_s=12	[0,400] ms	[42,240] ms	M100±12 ms	M50±6 ms
RMS	16.5±7.7	13.2±4.9	11.6±6.1	8.8±2.5
RD	41.3±18.2	40.2±20.2	58.3±20.7	72.7±15.7
CC	0.89±0.113	0.89±0.128	0.777±0.172	0.679±0.154

N_s=18	[0,400] ms	[42,240] ms	M100±12 ms	M50±6 ms
RMS	12.8±5.6	9.6±3	9.6±5.3	7.5±2
RD	32.8±15.5	30.5±16.8	49.1±19.2	62±12.4
CC	0.931±0.076	0.935±0.082	0.847±0.121	0.775±0.103

N_s=24	[0,400] ms	[42,240] ms	M100±12 ms	M50±6 ms
RMS	10.4±4.4	7.7±2	8.3±4.7	6.4±1.6
RD	27±13.7	25.1±15	43±16.5	53.4±11.8
CC	0.953±0.056	0.955±0.063	0.889±0.08	0.837±0.081

N_s=30	[0,400] ms	[42,240] ms	M100±12 ms	M50±6 ms
RMS	8.8±4	6.3±1.6	7.2±4.3	5.4±1.3
RD	23±12.2	20.8±13.2	37.4±15.2	45.2±9.7
CC	0.966±0.042	0.969±0.045	0.916±0.061	0.888±0.051

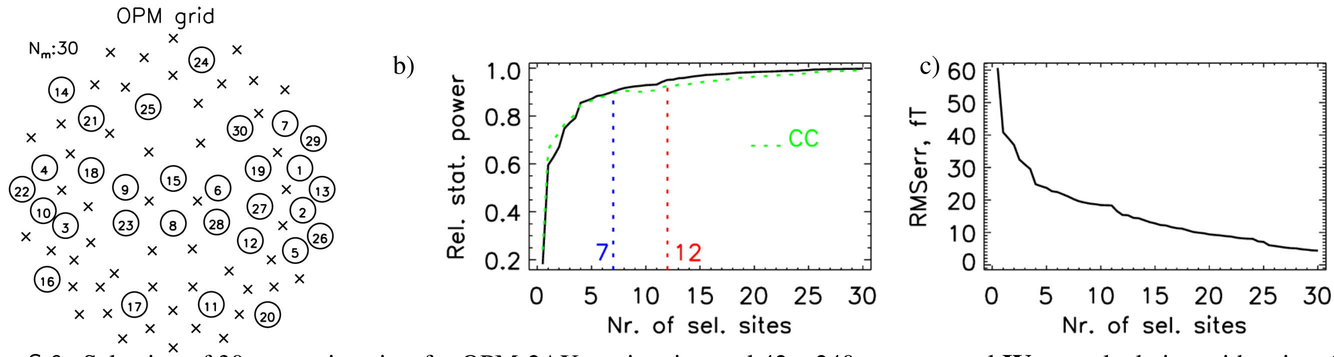

Fig. S.9: Selection of 30 measuring sites for OPM-2AX on time interval 42 – 240 ms, protocol **IV** – recalculation with pairs of channels on each selected site: a) OPM grid, encircled numbers show the order of selected channels, crosses denotes unselected channels, b) relative statistical power, and c) RMSerr vs. number of selected channels.

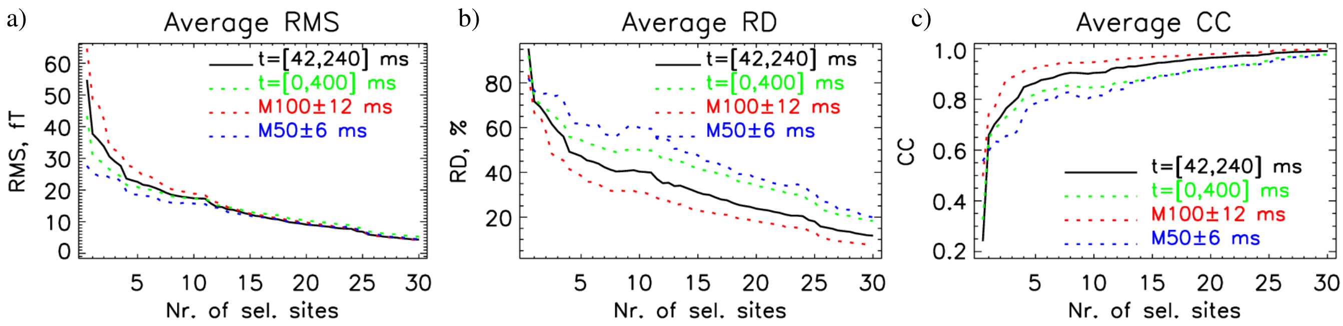

Fig. S.10: SSA protocol **IV**, evaluation measures RMS, RD and CC – recalculation with pairs of channels on each selected site.

N_m=12	[0,400] ms	[42,240] ms	M100±12 ms	M50±6 ms
RMS	19.4±6.6	20.5±6.6	23.5±6.9	17.3±7.3
RD	48.2±20.7	41.4±19	32.7±13.2	55.2±20.6
CC	0.859±0.134	0.896±0.107	0.942±0.053	0.829±0.119

N_m=18	[0,400] ms	[42,240] ms	M100±12 ms	M50±6 ms
RMS	16.5±5.9	16.8±5.8	18.4±5.7	15±6.7
RD	41.6±19.6	34±16.7	26±12.2	48.5±21.8
CC	0.891±0.115	0.93±0.076	0.961±0.04	0.869±0.105

N_m=24	[0,400] ms	[42,240] ms	M100±12 ms	M50±6 ms
RMS	14±4.5	13.6±4.1	14.8±4.3	13.3±6.5
RD	36.4±19.3	28.1±15.1	21.5±10.8	43.3±21.8
CC	0.914±0.1	0.95±0.062	0.972±0.029	0.894±0.096

N_m=30	[0,400] ms	[42,240] ms	M100±12 ms	M50±6 ms
RMS	11.8±3.9	11.1±3.1	11.3±3.1	10.7±4.2
RD	31.4±17.6	23.3±12.8	16.7±9	34.9±14.1
CC	0.934±0.082	0.965±0.045	0.982±0.021	0.933±0.051

S.1.5 Optimal locations for the SQUID system.

For the SQUID-MEG system we have on each of 125 measuring sites only one channel (first order axial gradiometer in radial direction). Therefore, we applied protocol **I**, which selects N_s channels using the SSA algorithm.

Figure S.11a shows the first 30 selected channels using protocol **III**. Figure S.12 displays results of evaluation on different time interval.

For comparison, the CC at learning time interval of [42,240] ms from figure S.11b is also shown S.11c. Relative statistical power exceeds values of 0.90 and 0.95 after 10th and 16th optimally selected channels.

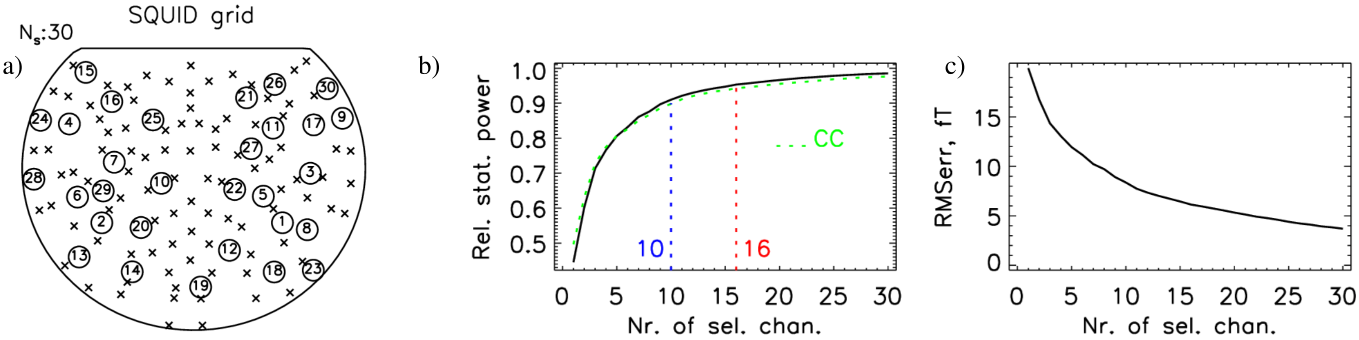

Fig. S.11: Selection of 30 measuring sites for SQUID-MEG on time interval 42 – 240 ms:a) SQUID grid, encircled numbers show the order of selected channels, crosses denotes unselected channels, b) relative statistical power, and c) RMSerr vs. number of selected channels.

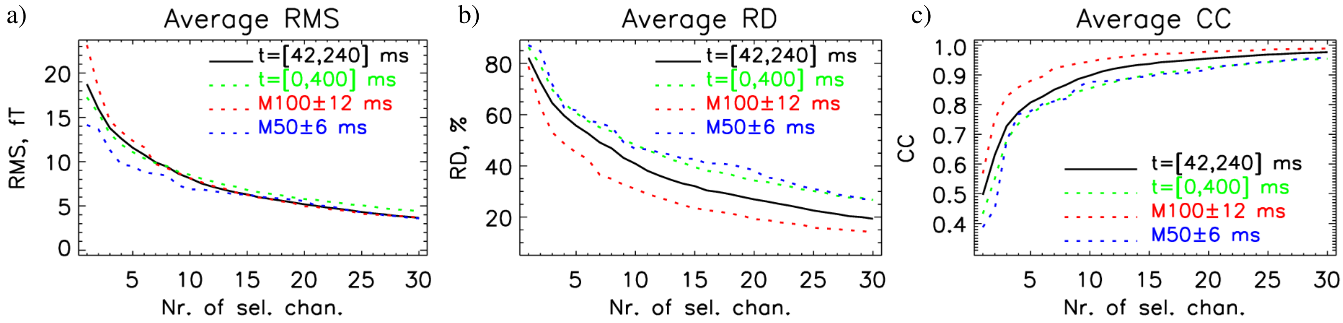

Fig. S.12: SSA on SQUID data, evaluation measures RMS, RD and CC.

N_s=12	[0,400] ms	[42,240] ms	M100±12 ms	M50±6 ms
RMS	7.4±2.5	6.8±1.6	6.9±1.2	6.3±2.7
RD	40.6±17.3	32.7±14	24.7±9.4	41.3±13.1
CC	0.895±0.1	0.934±0.066	0.965±0.028	0.901±0.062

N_s=18	[0,400] ms	[42,240] ms	M100±12 ms	M50±6 ms
RMS	5.7±1.9	5.2±1.2	5.1±1	5.4±2.2
RD	32.2±15.2	25.2±11.8	18.3±7.7	35.5±11.6
CC	0.933±0.069	0.96±0.045	0.98±0.019	0.928±0.045

N_s=24	[0,400] ms	[42,240] ms	M100±12 ms	M50±6 ms
RMS	4.6±1.5	4±0.8	3.9±0.9	4±1.1
RD	26.4±13	20±9.8	14.4±6.7	27.2±9.4
CC	0.956±0.048	0.975±0.03	0.987±0.014	0.958±0.031

N_s=30	[0,400] ms	[42,240] ms	M100±12 ms	M50±6 ms
RMS	3.8±1.1	3.2±0.5	3.2±0.5	3.1±0.6
RD	22.1±11.4	15.9±8.3	11.6±4.6	22±9.2
CC	0.969±0.036	0.984±0.022	0.992±0.006	0.971±0.028

S.1.6 Optimal locations for the OPM-2AX system using protocol III. for measuring data from the right hemisphere only

Here we search for optimal measuring sites from the set of 43 sites covering right hemisphere using protocol **III**.

Figure S.13a shows the first 21 selected sites using protocol **III**. Figure S.14 displays results of evaluation on different time interval.

For comparison, the CC at learning time interval of [42,240] ms from figure S.13b is also shown S.13c. Relative statistical power exceeds values of 0.90 and 0.95 already after 3rd and 5th optimally selected sites.

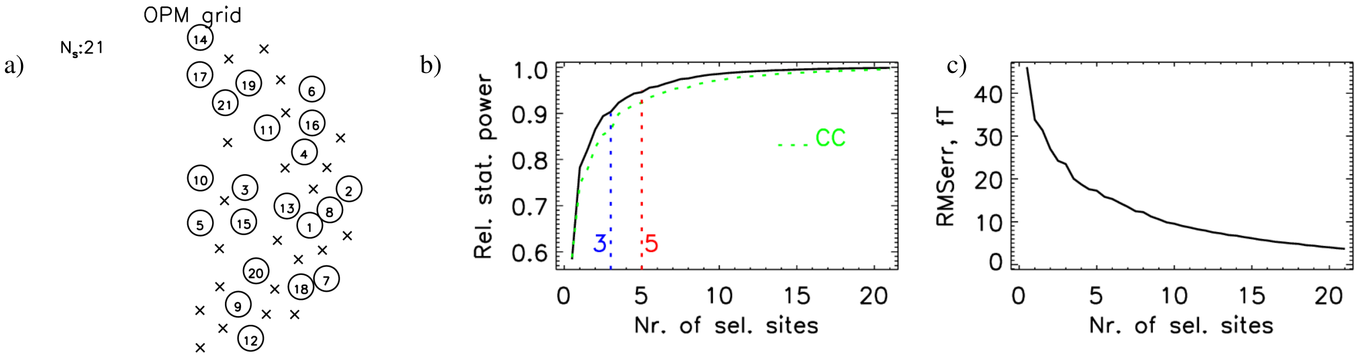

Fig. S.13: Selection of 21 measuring sites on the right hemisphere for OPM-2AX on time interval 42 – 240 ms, protocol **III**: a) OPM grid, encircled numbers show the order of selected sites, crosses denotes unselected sites, b) relative statistical power, and c) RMSerr vs. number of selected channels.

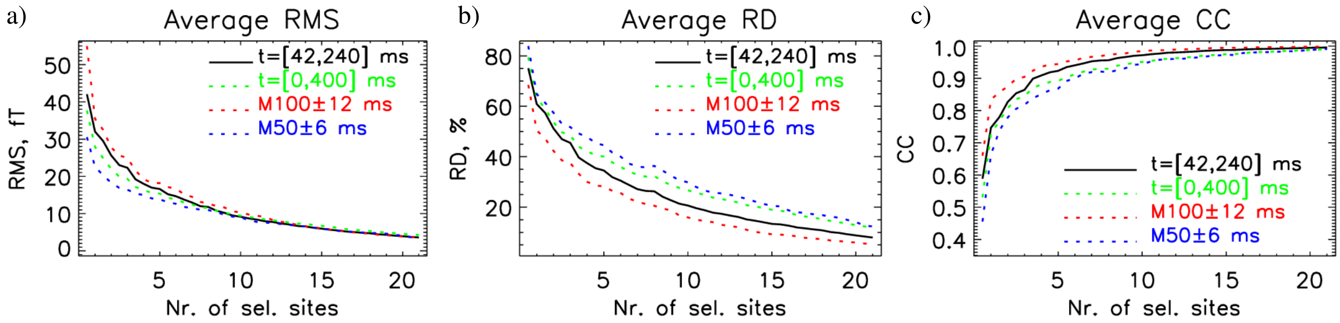

Fig. S.14: SSA protocol **III**, evaluation measures RMS, RD and CC.

N_m=6	[0,400] ms	[42,240] ms	M100±12 ms	M50±6 ms
RMS	18.6±6.6	21.5±7	24±5.9	15.8±7.3
RD	44.2±20.6	41.7±19.6	34.2±17.8	47.3±21
CC	0.869±0.135	0.886±0.115	0.92±0.089	0.851±0.133

N_m=12	[0,400] ms	[42,240] ms	M100±12 ms	M50±6 ms
RMS	12.7±3.9	13.6±4.1	15.5±4.1	11.7±4.6
RD	31.5±17.4	26.7±13.6	22.5±12.9	34.9±15.2
CC	0.932±0.088	0.954±0.055	0.966±0.041	0.925±0.068

N_m=15	[0,400] ms	[42,240] ms	M100±12 ms	M50±6 ms
RMS	10.6±3	10.9±3	11.9±3.3	10±4.5
RD	26.7±15.3	21.6±11.3	17.4±10.3	29.6±12.9
CC	0.951±0.067	0.97±0.038	0.979±0.025	0.947±0.049

N_m=21	[0,400] ms	[42,240] ms	M100±12 ms	M50±6 ms
RMS	7.9±2.4	7.6±2.1	8.5±3	7.1±2.7
RD	20.2±12.5	15.3±9	11.7±5.7	21.6±9.6
CC	0.971±0.044	0.984±0.028	0.992±0.008	0.972±0.027

S.2 Localizations of M100 and M50 for all measurements

Comparisons of M100 and M50 source localization for all cases. First we applied SSA (protocol **III**) to find the first 18 optimal measuring sites. Then we localized M100 and M50 with a single or two dipole sources from the measured MFM, the estimated MFM and from the selected channels only. All results are displayed in the following figures. In figures's captions, all coordinates and localization errors are expressed in units of mm, dipole moments in μAm, and dipole orientation errors with angular degrees. We applied one dipole source for cases where AEF signal is observed only on one hemisphere.

M100

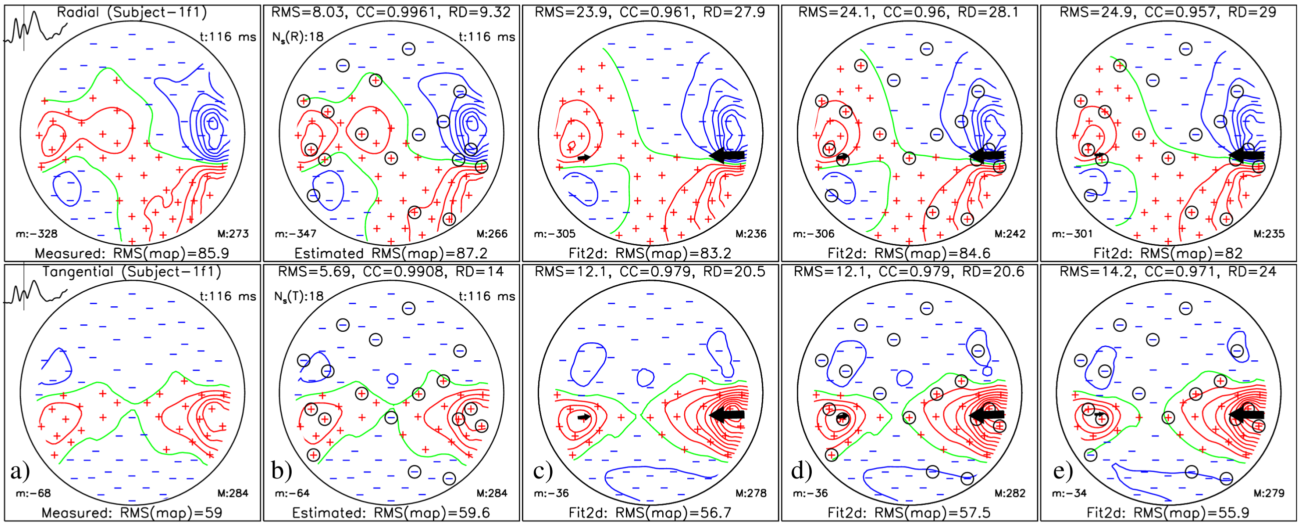

Fig. S.15: Subject-1f1: 18 selected sites using protocol **III**, fitting M100 with 2 dipoles:
a) Measured data, b) Estimated data map,
c) measured map fit: $\vec{r}_1$=(58.8,-15.3,18.7), $\vec{r}_2$=(-54.6,-19,32.1), $\vec{p}_1$=(3.1,0.7,-9.1), $\vec{p}_2$=(-1.7,-0.1,-2.9),
d) estimated map fit: $\vec{r}_1$=(58.5,-15.2,17.6), $\vec{r}_2$=(-55.3,-18.5,32), $\vec{p}_1$=(3.1,0.8,-9.6), $\vec{p}_2$=(-1.6,-0.2,-2.8),
reconstructed source errors: Δr_1=1.1, Δr_2=0.9, Δr_c=1.5, $\Delta\varphi_1$=0.888, $\Delta\varphi_2$=2.17,
e) selected chan. fit: $\vec{r}_1$=(58.4,-14.8,16.6), $\vec{r}_2$=(-60.1,-17.4,31.7), $\vec{p}_1$=(2.8,0.5,-9.5), $\vec{p}_2$=(-1.1,0.1,-2),
reconstructed source errors: Δr_1=2.1, Δr_2=5.7, Δr_c=6.1, $\Delta\varphi_1$=2.32, $\Delta\varphi_2$=3.95.

M50

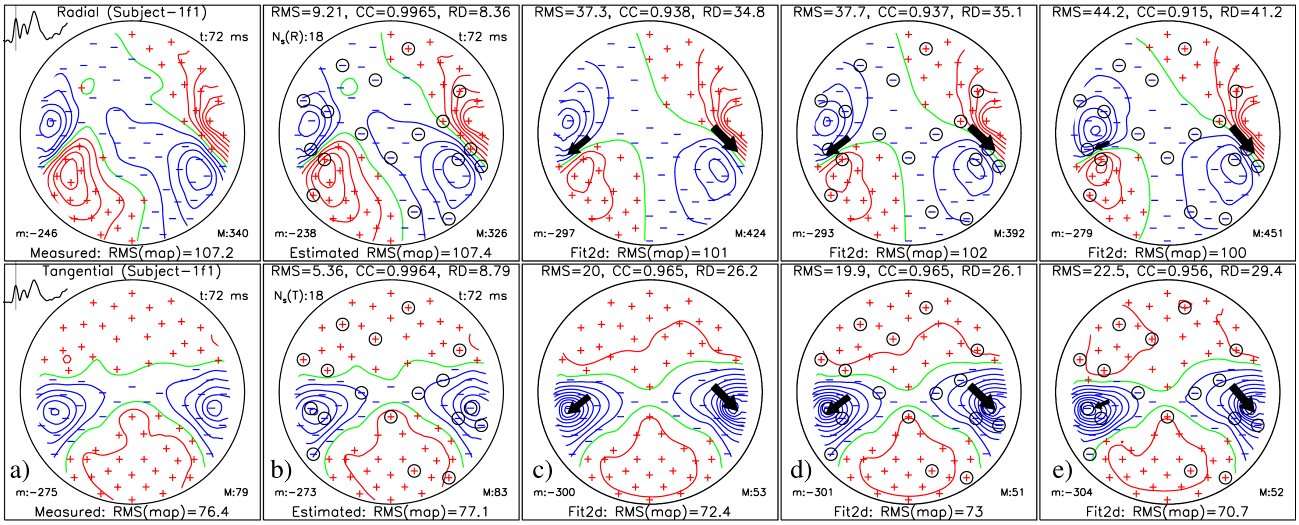

Fig. S.16: Subject-1f1: 18 selected sites using protocol **III**, fitting M50 with 2 dipoles:
a) Measured data, b) Estimated data map,
c) measured map fit: $\vec{r}_1$=(55.1,-3.4,20.4), $\vec{r}_2$=(-51.8,-8.3,26.5), $\vec{p}_1$=(-3.6,5.6,10.8), $\vec{p}_2$=(3.9,3.1,8.6),
d) estimated map fit: $\vec{r}_1$=(55.1,-2.8,22.5), $\vec{r}_2$=(-51.6,-8.5,26.7), $\vec{p}_1$=(-4,5.2,10.4), $\vec{p}_2$=(4,2.9,8.7),
reconstructed source errors: Δr_1=2.3, Δr_2=0.3, Δr_c=2.3, $\Delta\varphi_1$=2.41, $\Delta\varphi_2$=1.57,
e) selected chan. fit: $\vec{r}_1$=(55,-3.7,20.4), $\vec{r}_2$=(-56.6,-9.4,36.2), $\vec{p}_1$=(-3.5,6.7,10.8), $\vec{p}_2$=(3,1,5),
reconstructed source errors: Δr_1=0.4, Δr_2=10.9, Δr_c=10.9, $\Delta\varphi_1$=4.04, $\Delta\varphi_2$=11.

M100

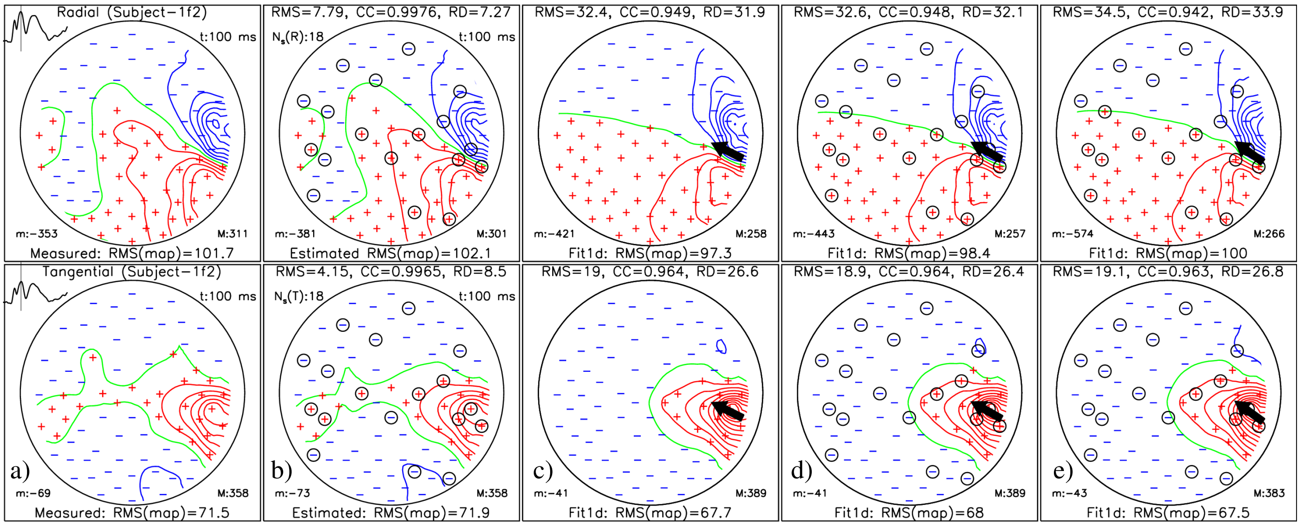

Fig. S.17: Subject-1f2: 18 selected sites using protocol **III**, fitting M100 with 1 dipole:
a) Measured data, b) Estimated data map,
c) measured map fit: $\vec{r}$=(58.4,-11.8,19.2), $\vec{p}$=(3.2,-2.4,-11.4),
d) estimated map fit – source parameters: $\vec{r}$=(58.2,-11.7,18.9), $\vec{p}$=(3.2,-2.8,-11.6),
reconstructed source errors: $\Delta\vec{r}$=(-0.2,0,-0.3), Δr=0.3, $\Delta\vec{p}$=(0,-0.4,-0.2), Δp=0.5, $\Delta\varphi$=0.029,
e) selected chan. fit: $\vec{r}$=(58.3,-12,14.5), $\vec{p}$=(2.4,-3.5,-12.6),
reconstructed source errors: $\Delta\vec{r}$=(-0.2,-0.2,-4.7), Δr=4.7, $\Delta\vec{p}$=(-0.8,-1,-1.2), Δp=1.8, $\Delta\varphi$=0.107.

M50

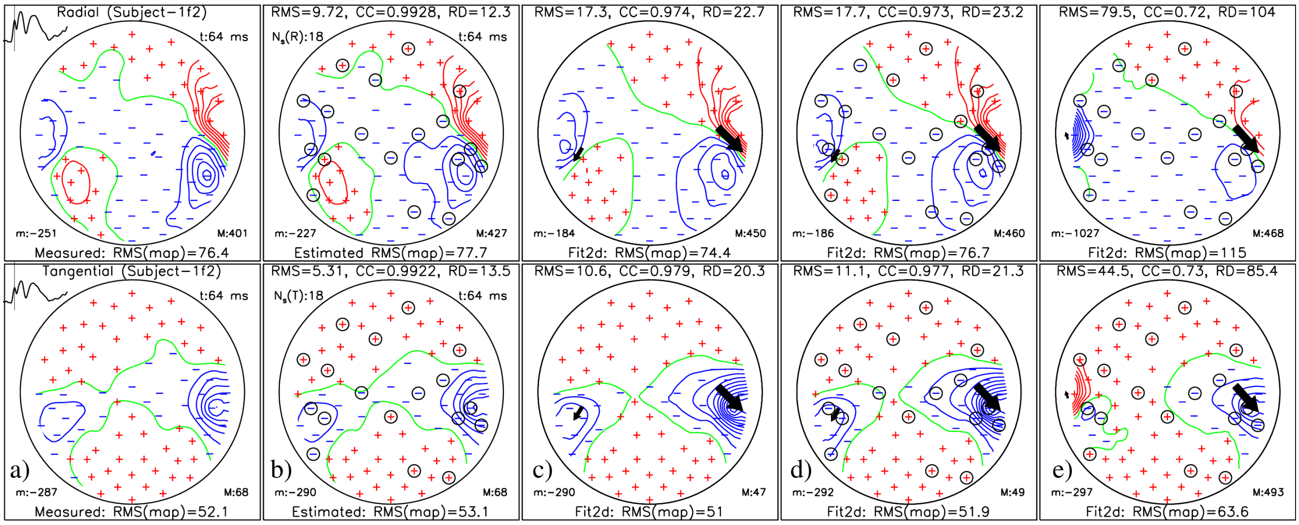

Fig. S.18: Subject-1f2: 18 selected sites using protocol **III**, fitting M50 with 2 dipoles:
a) Measured data, b) Estimated data map,
c) measured map fit: $\vec{r}_1$=(59.5,-3.7,16.3), $\vec{r}_2$=(-52.1,-14.6,24.8), $\vec{p}_1$=(-2,3.9,8.2), $\vec{p}_2$=(1,2.1,3.3),
d) estimated map fit: $\vec{r}_1$=(58.9,-3.1,17), $\vec{r}_2$=(-52.8,-15.4,22.7), $\vec{p}_1$=(-2.2,4.5,8.5), $\vec{p}_2$=(0.8,2.1,3.3),
reconstructed source errors: Δr_1=1.1, Δr_2=2.4, Δr_c=2.6, $\Delta\varphi_1$=2.67, $\Delta\varphi_2$=2.91,
e) selected chan. fit: $\vec{r}_1$=(61,-3.8,18.3), $\vec{r}_2$=(-83.4,-0.9,-6.4), $\vec{p}_1$=(-1.8,4,7), $\vec{p}_2$=(0,-1.4,0.6),
reconstructed source errors: Δr_1=2.6, Δr_2=46.2, Δr_c=46.3, $\Delta\varphi_1$=4.25, $\Delta\varphi_2$=97.6.

M100

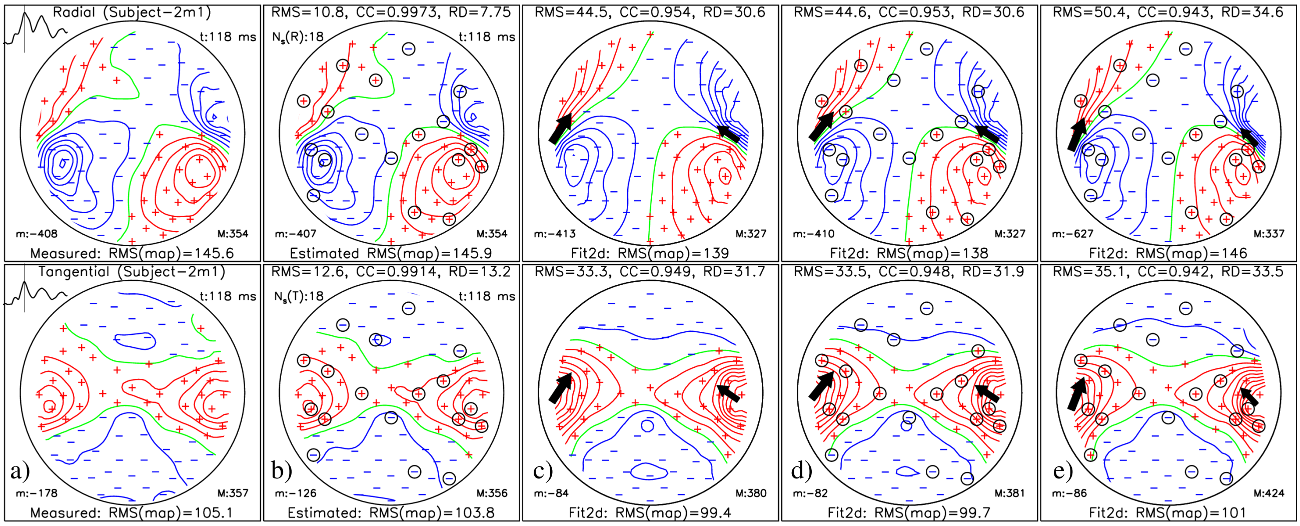

Fig. S.19: Subject-2m1: 18 selected sites using protocol **III**, fitting M100 with 2 dipoles:
a) Measured data, b) Estimated data map,
c) measured map fit: $\vec{r}_1$=(44.5,0.9,15.1), $\vec{r}_2$=(-37.8,3,6.2), $\vec{p}_1$=(10.5,-12.1,-30.1), $\vec{p}_2$=(-8.1,-26.3,-36.7),
d) estimated map fit: $\vec{r}_1$=(44.8,0.9,15.3), $\vec{r}_2$=(-38.2,4.1,7.6), $\vec{p}_1$=(10.4,-11.4,-29.6), $\vec{p}_2$=(-9.4,-23.2,-34.8),
reconstructed source errors: Δr_1=0.3, Δr_2=1.9, Δr_c=1.9, $\Delta\varphi_1$=0.822, $\Delta\varphi_2$=3.19,
e) selected chan. fit: $\vec{r}_1$=(51.8,-1.1,14.6), $\vec{r}_2$=(-41.6,0.4,5.3), $\vec{p}_1$=(5.9,-11.3,-22), $\vec{p}_2$=(-3.8,-25.9,-28.1),
reconstructed source errors: Δr_1=7.5, Δr_2=4.7, Δr_c=8.9, $\Delta\varphi_1$=6.76, $\Delta\varphi_2$=8.27.

M50

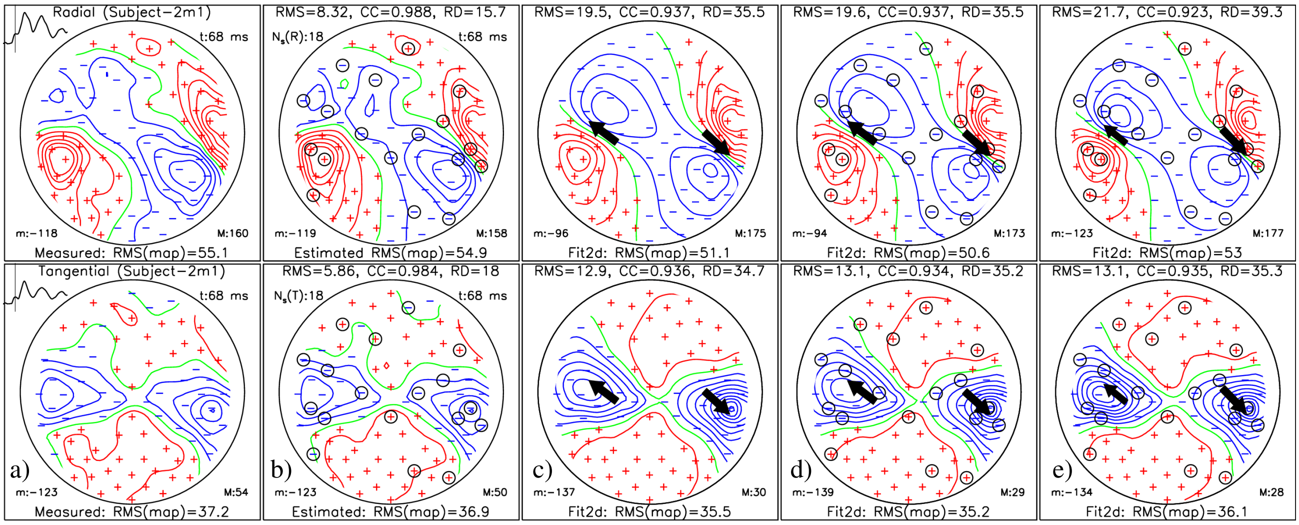

Fig. S.20: Subject-2m1: 18 selected sites using protocol **III**, fitting M50 with 2 dipoles:
a) Measured data, b) Estimated data map,
c) measured map fit: $\vec{r}_1$=(49.7,-6.4,27.3), $\vec{r}_2$=(-34.1,2.1,41.7), $\vec{p}_1$=(-3.2,2.9,6.5), $\vec{p}_2$=(5.4,-3.3,4.6),
d) estimated map fit: $\vec{r}_1$=(50.9,-6.6,26.1), $\vec{r}_2$=(-35.3,2,42.8), $\vec{p}_1$=(-2.8,2.6,6.2), $\vec{p}_2$=(5.1,-3.1,4.3),
reconstructed source errors: Δr_1=1.7, Δr_2=1.7, Δr_c=2.4, $\Delta\varphi_1$=1.55, $\Delta\varphi_2$=0.155,
e) selected chan. fit: $\vec{r}_1$=(51.2,-5.1,28.7), $\vec{r}_2$=(-43.6,0.3,44.8), $\vec{p}_1$=(-2.7,3,5.4), $\vec{p}_2$=(3.6,-2.4,3.5),
reconstructed source errors: Δr_1=2.4, Δr_2=10.2, Δr_c=10.5, $\Delta\varphi_1$=5.01, $\Delta\varphi_2$=3.75.

M100

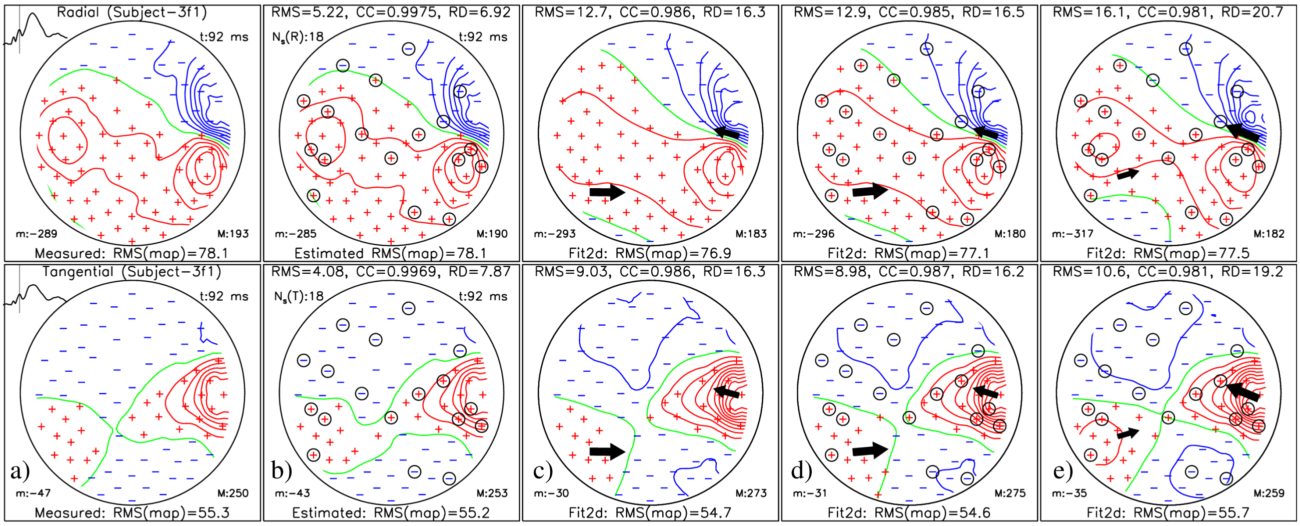

Fig. S.21: Subject-3f1: 18 selected sites using protocol **III**, fitting M100 with 2 dipoles:
a) Measured data, b) Estimated data map,
c) measured map fit: $\vec{r}_1$=(51.6,1.6,19), $\vec{r}_2$=(-13.5,-18.6,11.8), $\vec{p}_1$=(5,-2.3,-13.5), $\vec{p}_2$=(-14.3,1.9,-13.4),
d) estimated map fit: $\vec{r}_1$=(51.4,1.7,18.9), $\vec{r}_2$=(-12.1,-18.1,12.7), $\vec{p}_1$=(5.1,-2.6,-13.7), $\vec{p}_2$=(-15,0.3,-13.9),
reconstructed source errors: Δr_1=0.2, Δr_2=1.7, Δr_c=1.7, $\Delta\varphi_1$=1.15, $\Delta\varphi_2$=4.63,
e) selected chan. fit: $\vec{r}_1$=(52.4,1.9,21.6), $\vec{r}_2$=(-24.4,-24.6,31.3), $\vec{p}_1$=(5.1,-3.3,-12.1), $\vec{p}_2$=(-6,-1.4,-5.8),
reconstructed source errors: Δr_1=2.8, Δr_2=23.1, Δr_c=23.3, $\Delta\varphi_1$=5.58, $\Delta\varphi_2$=15.

M50

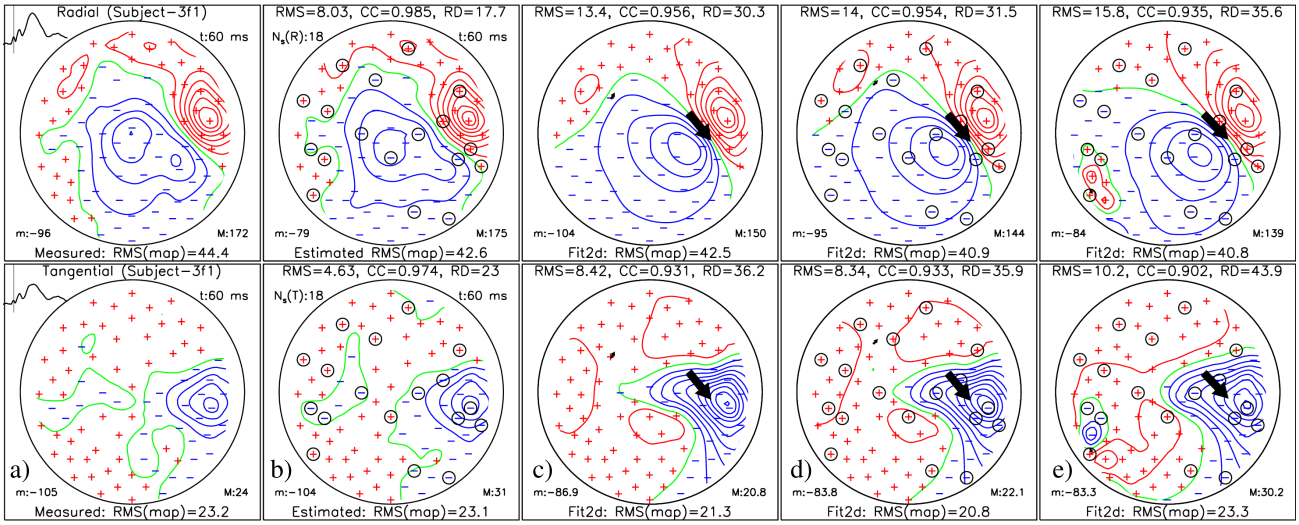

Fig. S.22: Subject-3f1: 18 selected sites using protocol **III**, fitting M50 with 2 dipoles:
a) Measured data, b) Estimated data map,
c) measured map fit: $\vec{r}_1$=(45,7.2,45.2), $\vec{r}_2$=(-32,34.8,46.1), $\vec{p}_1$=(-3.1,4.2,2.4), $\vec{p}_2$=(-0.5,-0.8,0.2),
d) estimated map fit: $\vec{r}_1$=(44,5.9,43.7), $\vec{r}_2$=(-29.3,47.8,41.5), $\vec{p}_1$=(-3.2,4.2,2.6), $\vec{p}_2$=(-0.7,-0.6,0.2),
reconstructed source errors: Δr_1=2.1, Δr_2=14.1, Δr_c=14.2, $\Delta\varphi_1$=1.51, $\Delta\varphi_2$=14.2,
e) selected chan. fit: $\vec{r}_1$=(38.6,6.3,40.7), $\vec{r}_2$=(-65.1,-49.2,2.9), $\vec{p}_1$=(-4.7,5.2,3.6), $\vec{p}_2$=(-0.6,0.7,0),
reconstructed source errors: Δr_1=7.8, Δr_2=100.1, Δr_c=100.4, $\Delta\varphi_1$=5.57, $\Delta\varphi_2$=109.

M10

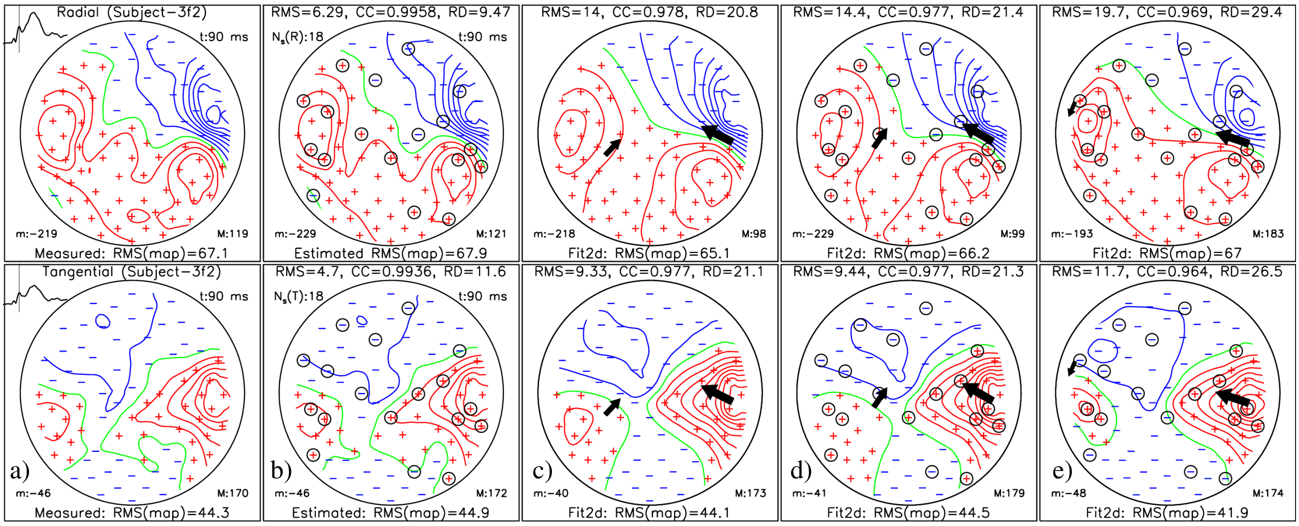

Fig. S.23: Subject-3f2: 18 selected sites using protocol **III**, fitting M100 with 2 dipoles:
a) Measured data, b) Estimated data map,
c) measured map fit: $\vec{r}_1$=(38.5,0.4,21.5), $\vec{r}_2$=(-18.1,-6.2,30.1), $\vec{p}_1$=(9.8,-6.5,-17.4), $\vec{p}_2$=(-7.6,-8.4,-6.3),
d) estimated map fit: $\vec{r}_1$=(40.9,1.3,21.1), $\vec{r}_2$=(-14.1,-1.6,33.4), $\vec{p}_1$=(8.3,-6.5,-15.7), $\vec{p}_2$=(-6.8,-10,-3.3),
reconstructed source errors: Δr_1=2.6, Δr_2=6.9, Δr_c=7.3, $\Delta\varphi_1$=2.42, $\Delta\varphi_2$=15.5,
e) selected chan. fit: $\vec{r}_1$=(45.3,-2.1,27.6), $\vec{r}_2$=(-58.5,16.1,-2.5), $\vec{p}_1$=(5.8,-2.4,-9.8), $\vec{p}_2$=(1.4,4.9,-0.3),
reconstructed source errors: Δr_1=9.5, Δr_2=56.5, Δr_c=57.3, $\Delta\varphi_1$=6.4, $\Delta\varphi_2$=139.

M50

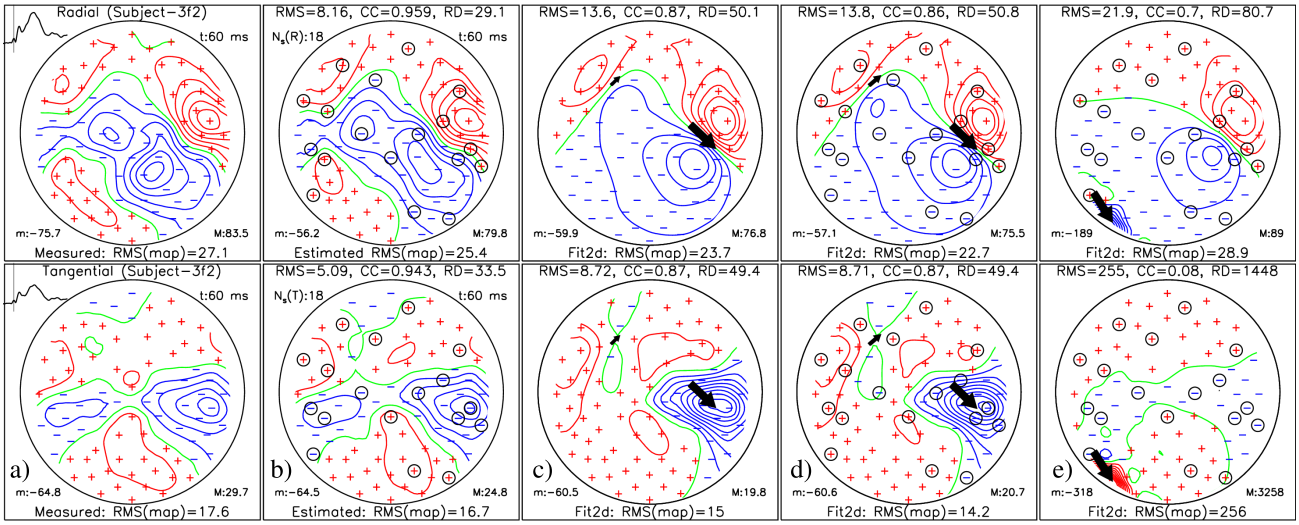

Fig. S.24: Subject-3f2: 18 selected sites using protocol **III**, fitting M50 with 2 dipoles:
a) Measured data, b) Estimated data map,
c) measured map fit: $\vec{r}_1$=(49.1,-1.7,44.8), $\vec{r}_2$=(-29.8,48.7,39), $\vec{p}_1$=(-1.7,1.4,1.9), $\vec{p}_2$=(-0.7,-0.6,0.2),
d) estimated map fit: $\vec{r}_1$=(51.4,-2.3,43.1), $\vec{r}_2$=(-28.9,48.5,38.6), $\vec{p}_1$=(-1.4,1.2,1.8), $\vec{p}_2$=(-0.9,-0.6,0.1),
reconstructed source errors: Δr_1=2.9, Δr_2=1, Δr_c=3.1, $\Delta\varphi_1$=2.38, $\Delta\varphi_2$=4.41,
e) selected chan. fit: $\vec{r}_1$=(48.9,2.9,46.7), $\vec{r}_2$=(-33381,-38486,-596.1), $\vec{p}_1$=(-1.8,1.7,1.8), $\vec{p}_2$=(-332.2,285.6,164.3),
reconstructed source errors: Δr_1=4.9, Δr_2=50967, Δr_c=50967, $\Delta\varphi_1$=5.05, $\Delta\varphi_2$=76.7.

M100

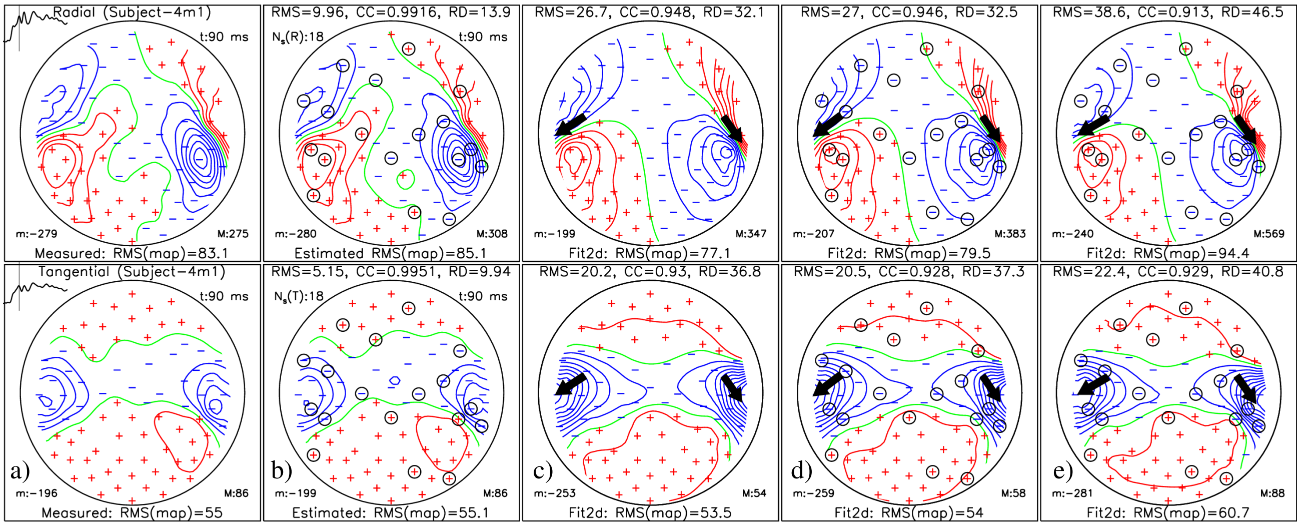

Fig. S.25: Subject-4m1: 18 selected sites using protocol **III**, fitting M100 with 2 dipoles:
a) Measured data, b) Estimated data map,
c) measured map fit: $\vec{r}_1$=(54.7,3.5,13), $\vec{r}_2$=(-46.2,5.4,15.3), $\vec{p}_1$=(-2.5,8,8.3), $\vec{p}_2$=(4.3,4.6,11.3),
d) estimated map fit: $\vec{r}_1$=(54.4,3.7,13.5), $\vec{r}_2$=(-45.3,5.1,13.1), $\vec{p}_1$=(-2.7,9,8.6), $\vec{p}_2$=(4.2,5.9,12.2),
reconstructed source errors: Δr_1=0.6, Δr_2=2.4, Δr_c=2.5, $\Delta\varphi_1$=2.36, $\Delta\varphi_2$=4.05,
e) selected chan. fit: $\vec{r}_1$=(60.7,3.7,19.9), $\vec{r}_2$=(-49.4,5.9,21.2), $\vec{p}_1$=(-2.6,6.9,6.5), $\vec{p}_2$=(4.1,3.1,8.7),
reconstructed source errors: Δr_1=9.1, Δr_2=6.8, Δr_c=11.3, $\Delta\varphi_1$=3.83, $\Delta\varphi_2$=5.22.

M50

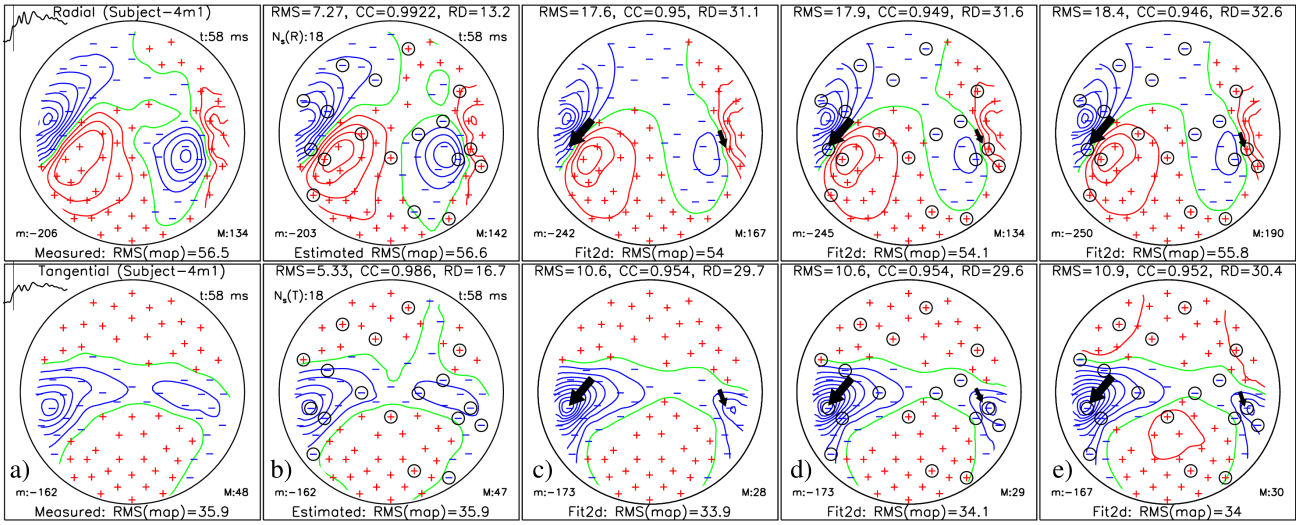

Fig. S.26: Subject-4m1: 18 selected sites using protocol **III**, fitting M50 with 2 dipoles:
a) Measured data, b) Estimated data map,
c) measured map fit: $\vec{r}_1$=(55.9,-3.2,24.4), $\vec{r}_2$=(-50.4,1.3,29), $\vec{p}_1$=(-0.5,3.2,1.7), $\vec{p}_2$=(3,4.5,5),
d) estimated map fit: $\vec{r}_1$=(56.4,-1.2,27.4), $\vec{r}_2$=(-50.2,1.6,29.4), $\vec{p}_1$=(-0.7,2.6,1.6), $\vec{p}_2$=(3.1,4.6,5.1),
reconstructed source errors: Δr_1=3.6, Δr_2=0.6, Δr_c=3.7, $\Delta\varphi_1$=5.94, $\Delta\varphi_2$=0.694,
e) selected chan. fit: $\vec{r}_1$=(58.9,-4,23), $\vec{r}_2$=(-49.3,2.9,31.2), $\vec{p}_1$=(-0.4,2.9,1.5), $\vec{p}_2$=(3.4,4.9,4.9),
reconstructed source errors: Δr_1=3.4, Δr_2=3, Δr_c=4.5, $\Delta\varphi_1$=2.23, $\Delta\varphi_2$=3.87.

M100

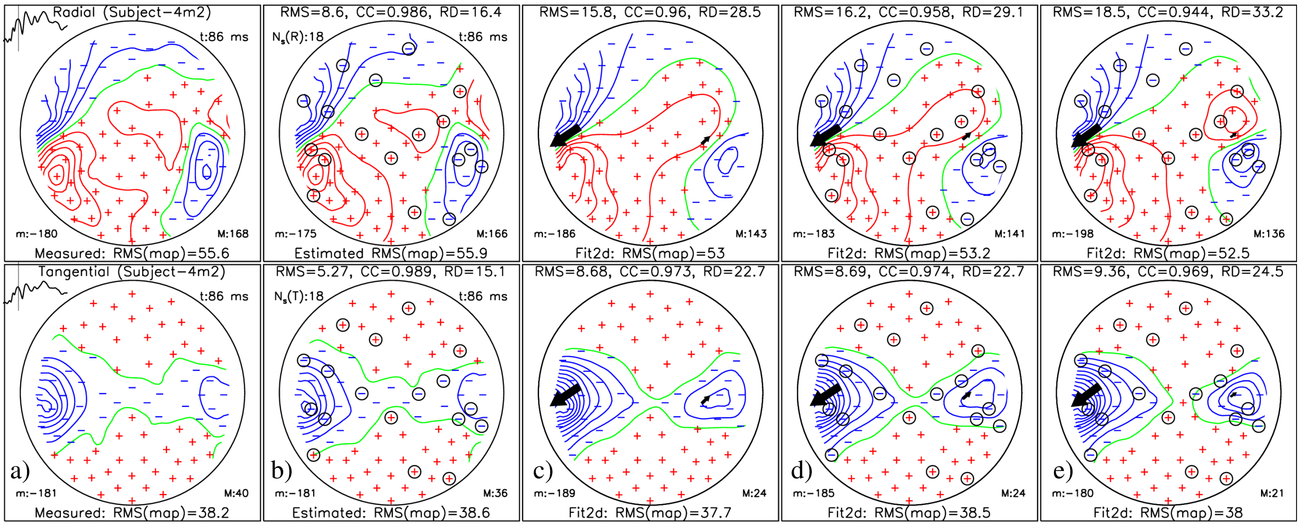

Fig. S.27: Subject-4m2: 18 selected sites using protocol **III**, fitting M100 with 2 dipoles:
a) Measured data, b) Estimated data map,
c) measured map fit: $\vec{r}_1$=(47.1,-4.3,39.9), $\vec{r}_2$=(-46.6,-0.9,11), $\vec{p}_1$=(-2,-1.9,2.1), $\vec{p}_2$=(2.5,3.4,10.9),
d) estimated map fit: $\vec{r}_1$=(47.3,-0.8,37.6), $\vec{r}_2$=(-45.3,-0.8,11.7), $\vec{p}_1$=(-2.1,-1.5,2.6), $\vec{p}_2$=(3,3.7,11.7),
reconstructed source errors: Δr_1=4.2, Δr_2=1.5, Δr_c=4.4, $\Delta\varphi_1$=9.74, $\Delta\varphi_2$=1.21,
e) selected chan. fit: $\vec{r}_1$=(62,-0.2,37.6), $\vec{r}_2$=(-48.5,-0.9,14), $\vec{p}_1$=(-0.9,-0.6,1.5), $\vec{p}_2$=(2.6,3.3,9.1),
reconstructed source errors: Δr_1=15.7, Δr_2=3.6, Δr_c=16.1, $\Delta\varphi_1$=18.9, $\Delta\varphi_2$=3.6.

M50

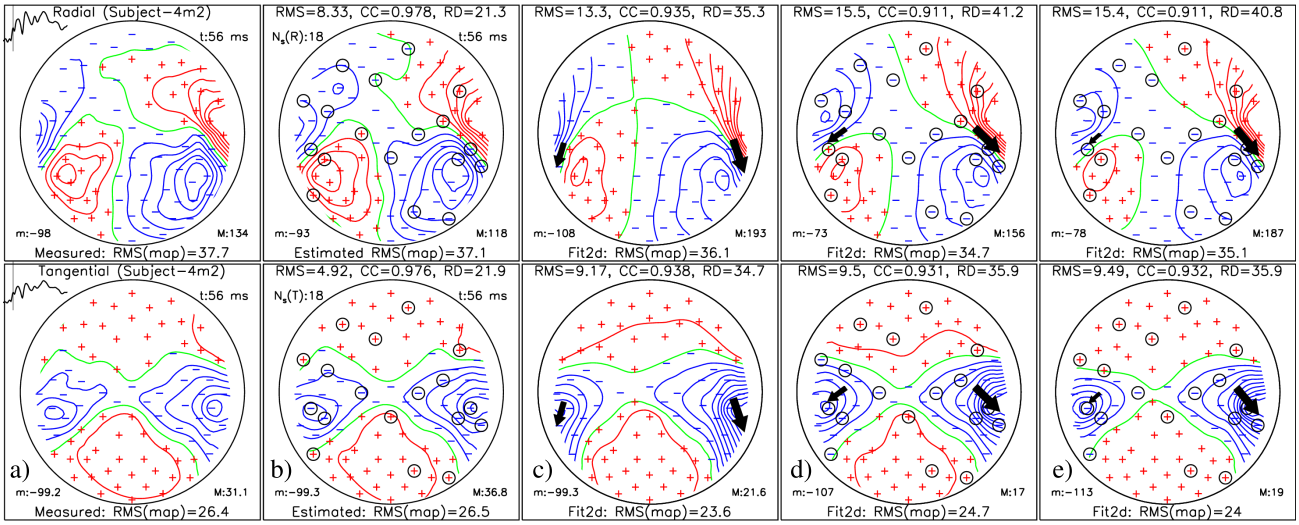

Fig. S.28: Subject-4m2: 18 selected sites using protocol **III**, fitting M50 with 2 dipoles:
a) Measured data, b) Estimated data map,
c) measured map fit: $\vec{r}_1$=(45.5,-10.8,2.7), $\vec{r}_2$=(-41.2,-9.5,4.6), $\vec{p}_1$=(1.2,7.5,9.1), $\vec{p}_2$=(-0.7,5.9,5.9),
d) estimated map fit: $\vec{r}_1$=(48.2,-3.2,14.9), $\vec{r}_2$=(-41.5,-0.5,21.4), $\vec{p}_1$=(-1.9,3.3,6.9), $\vec{p}_2$=(2.1,1.7,4.2),
reconstructed source errors: Δr_1=14.6, Δr_2=19.1, Δr_c=24, $\Delta\varphi_1$=24.1, $\Delta\varphi_2$=37.8,
e) selected chan. fit: $\vec{r}_1$=(52.2,-4.8,14.7), $\vec{r}_2$=(-50.8,-2.9,24.9), $\vec{p}_1$=(-1.3,3.1,5.5), $\vec{p}_2$=(1,1,2.2),
reconstructed source errors: Δr_1=14.9, Δr_2=23.4, Δr_c=27.8, $\Delta\varphi_1$=19.7, $\Delta\varphi_2$=33.5.

M100

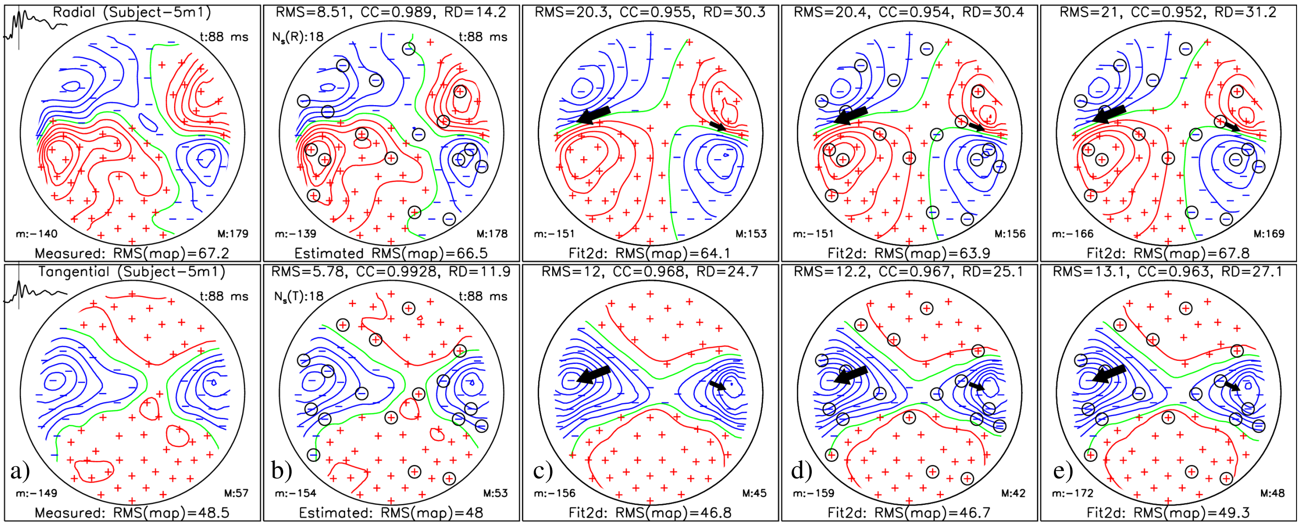

Fig. S.29: Subject-5m1: 18 selected sites using protocol **III**, fitting M100 with 2 dipoles:
a) Measured data, b) Estimated data map,
c) measured map fit: $\vec{r}_1$=(50.6,6.3,27.3), $\vec{r}_2$=(-35,12.2,29.1), $\vec{p}_1$=(-3.6,1.4,6.4), $\vec{p}_2$=(10.5,3.2,11.3),
d) estimated map fit: $\vec{r}_1$=(52.3,5.9,28.3), $\vec{r}_2$=(-36.4,12,28.6), $\vec{p}_1$=(-3.2,1,5.7), $\vec{p}_2$=(9.6,2.9,11),
reconstructed source errors: Δr_1=2, Δr_2=1.5, Δr_c=2.5, $\Delta\varphi_1$=2.3, $\Delta\varphi_2$=1.86,
e) selected chan. fit: $\vec{r}_1$=(49.6,5.9,30.9), $\vec{r}_2$=(-36.3,12.6,27.6), $\vec{p}_1$=(-4.1,1.5,6.2), $\vec{p}_2$=(10.5,3.6,12.2),
reconstructed source errors: Δr_1=3.7, Δr_2=2.1, Δr_c=4.3, $\Delta\varphi_1$=3.55, $\Delta\varphi_2$=2.35.

M50

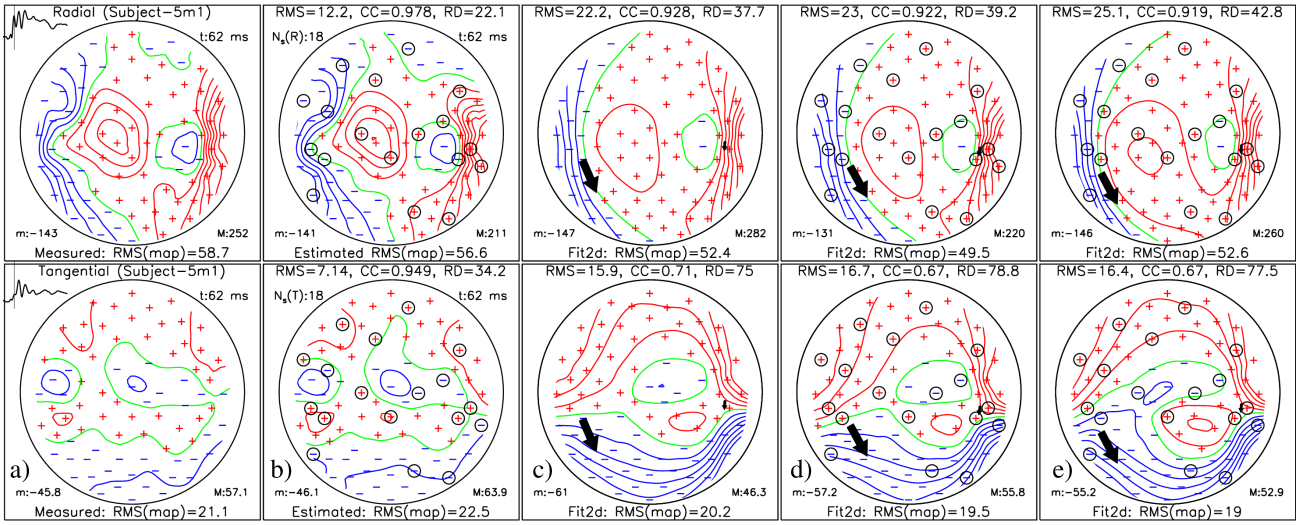

Fig. S.30: Subject-5m1: 18 selected sites using protocol **III**, fitting M50 with 2 dipoles:
a) Measured data, b) Estimated data map,
c) measured map fit: $\vec{r}_1$=(42.1,-6.2,15.9), $\vec{r}_2$=(-13.7,-9.7,8), $\vec{p}_1$=(2.3,14.7,-0.3), $\vec{p}_2$=(-30.1,51.9,11.2),
d) estimated map fit: $\vec{r}_1$=(48.2,-11.1,21.6), $\vec{r}_2$=(-15.5,-15,12.5), $\vec{p}_1$=(1.9,7.8,-0.1), $\vec{p}_2$=(-18.8,26.4,8.4),
reconstructed source errors: Δr_1=9.7, Δr_2=7.3, Δr_c=12.1, $\Delta\varphi_1$=4.58, $\Delta\varphi_2$=6.55,
e) selected chan. fit: $\vec{r}_1$=(45.7,-8.6,18), $\vec{r}_2$=(-16.6,-15.7,8), $\vec{p}_1$=(1.9,10.6,0.4), $\vec{p}_2$=(-26.5,34.7,13.2),
reconstructed source errors: Δr_1=4.8, Δr_2=6.7, Δr_c=8.3, $\Delta\varphi_1$=3.14, $\Delta\varphi_2$=9.45.

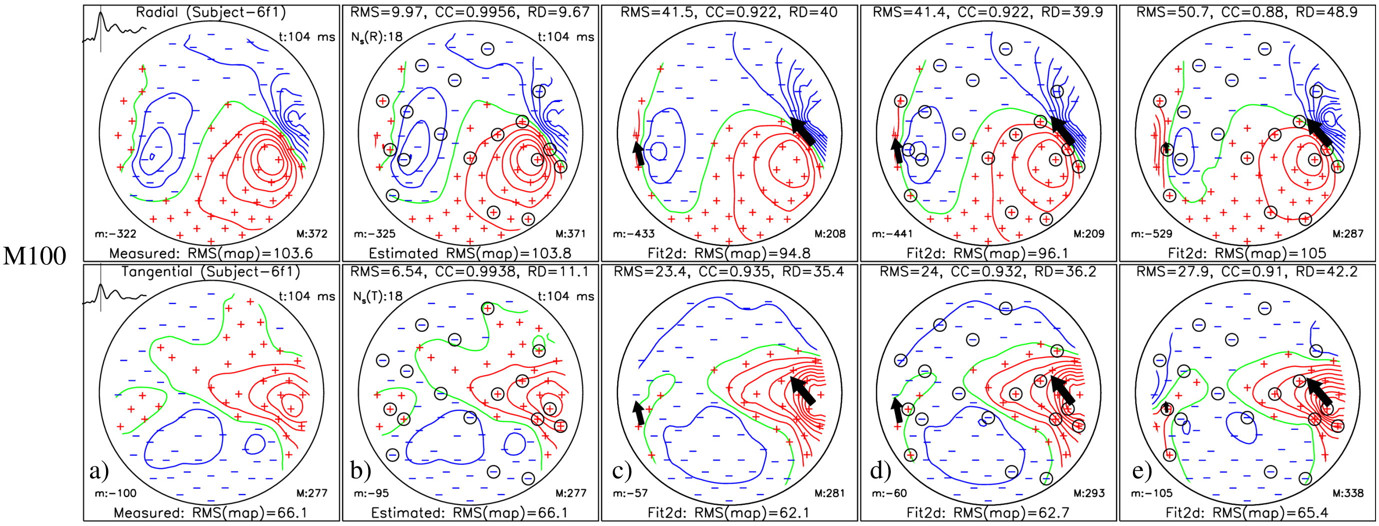

Fig. S.31: Subject-6f1: 18 selected sites using protocol **III**, fitting M100 with 2 dipoles:
a) Measured data, b) Estimated data map,
c) measured map fit: $\vec{r}_1$=(49.1,2.9,21.5), $\vec{r}_2$=(-43.4,-9,4.4), $\vec{p}_1$=(7,-11.4,-14.3), $\vec{p}_2$=(2.4,-12.9,-2.7),
d) estimated map fit: $\vec{r}_1$=(47.5,3.1,19.9), $\vec{r}_2$=(-41.5,-7.2,4.2), $\vec{p}_1$=(7.6,-13.5,-16), $\vec{p}_2$=(2.3,-15.4,-3.8),
reconstructed source errors: Δr_1=2.3, Δr_2=2.6, Δr_c=3.4, $\Delta\varphi_1$=1.78, $\Delta\varphi_2$=2.71,
e) selected chan. fit: $\vec{r}_1$=(56.9,2.5,27), $\vec{r}_2$=(-63,-9.9,19.4), $\vec{p}_1$=(4.8,-7.4,-9.4), $\vec{p}_2$=(0.3,-3.7,-1),
reconstructed source errors: Δr_1=9.6, Δr_2=24.8, Δr_c=26.6, $\Delta\varphi_1$=1.02, $\Delta\varphi_2$=6.53.

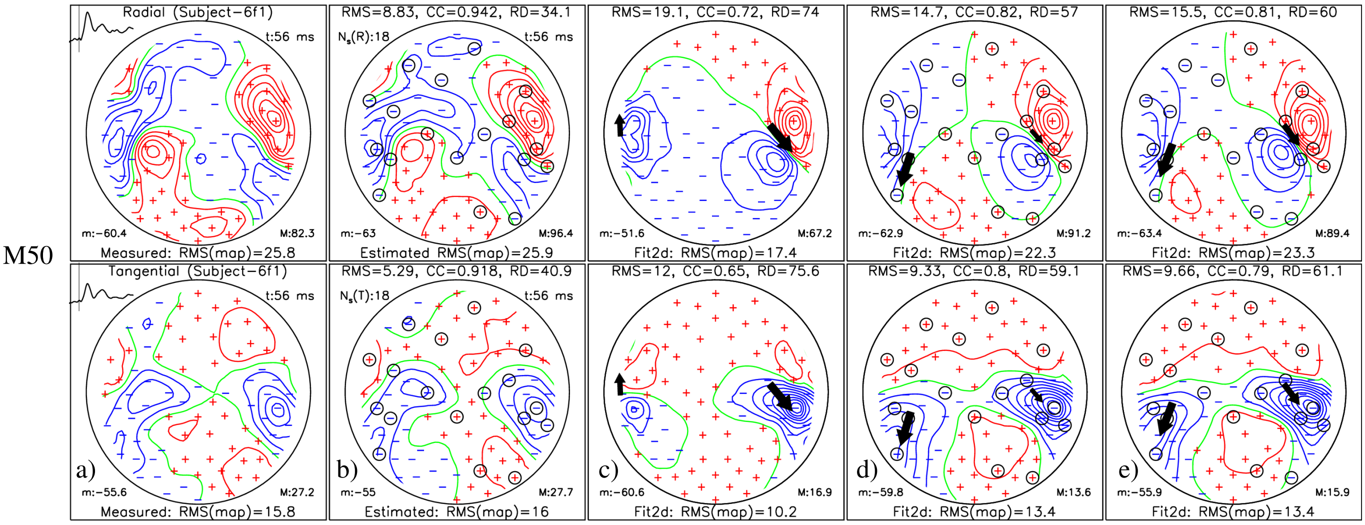

Fig. S.32: Subject-6f1: 18 selected sites using protocol **III**, fitting M50 with 2 dipoles:
a) Measured data, b) Estimated data map,
c) measured map fit: $\vec{r}_1$=(63.2,-3.5,38), $\vec{r}_2$=(-71.3,7.1,1.2), $\vec{p}_1$=(-0.5,0.6,0.8), $\vec{p}_2$=(-0.1,-0.7,0),
d) estimated map fit: $\vec{r}_1$=(56.8,-1.7,38.2), $\vec{r}_2$=(-36.3,-18.8,16.2), $\vec{p}_1$=(-0.9,1.1,1.4), $\vec{p}_2$=(0.2,2.5,3.4),
reconstructed source errors: Δr_1=6.7, Δr_2=46.1, Δr_c=46.5, $\Delta\varphi_1$=3.11, $\Delta\varphi_2$=127,
e) selected chan. fit: $\vec{r}_1$=(57.3,-0.5,44.5), $\vec{r}_2$=(-44,-17.1,24.2), $\vec{p}_1$=(-0.8,1.3,1.1), $\vec{p}_2$=(0.4,1.7,1.9),
reconstructed source errors: Δr_1=9.2, Δr_2=43.2, Δr_c=44.2, $\Delta\varphi_1$=10.5, $\Delta\varphi_2$=132.

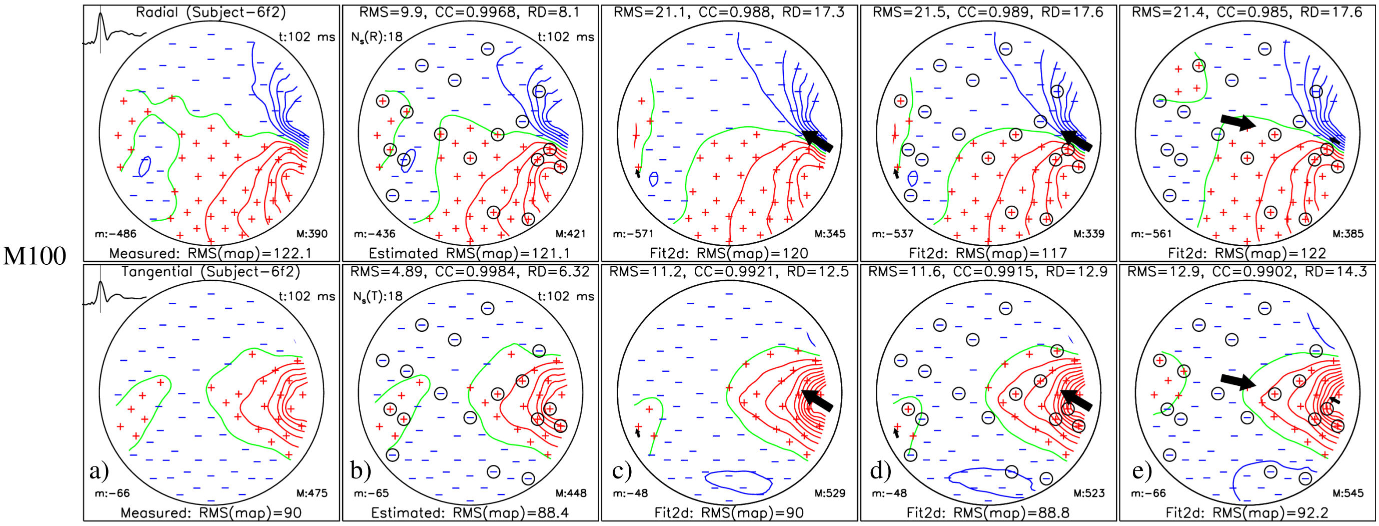

Fig. S.33: Subject-6f2: 18 selected sites using protocol **III**, fitting M100 with 2 dipoles:
a) Measured data, b) Estimated data map,
c) measured map fit: $\vec{r}_1$=(52.8,-3.1,7.4), $\vec{r}_2$=(-52.1,-22.3,-2.7), $\vec{p}_1$=(3,-5.8,-23.6), $\vec{p}_2$=(1.9,-4.1,-3.1),
d) estimated map fit: $\vec{r}_1$=(52,-2.8,7.2), $\vec{r}_2$=(-49.6,-21.1,-3.3), $\vec{p}_1$=(3.1,-5.7,-24.3), $\vec{p}_2$=(2.3,-4.8,-3.6),
reconstructed source errors: Δr_1=0.9, Δr_2=2.8, Δr_c=3, $\Delta\varphi_1$=0.551, $\Delta\varphi_2$=0.755,
e) selected chan. fit: $\vec{r}_1$=(50,-3.2,7), $\vec{r}_2$=(-0.8,1.3,7), $\vec{p}_1$=(4.1,-6.8,-32), $\vec{p}_2$=(-102.3,23.1,-15.9),
reconstructed source errors: Δr_1=2.8, Δr_2=57.3, Δr_c=57.4, $\Delta\varphi_1$=1.88, $\Delta\varphi_2$=115.

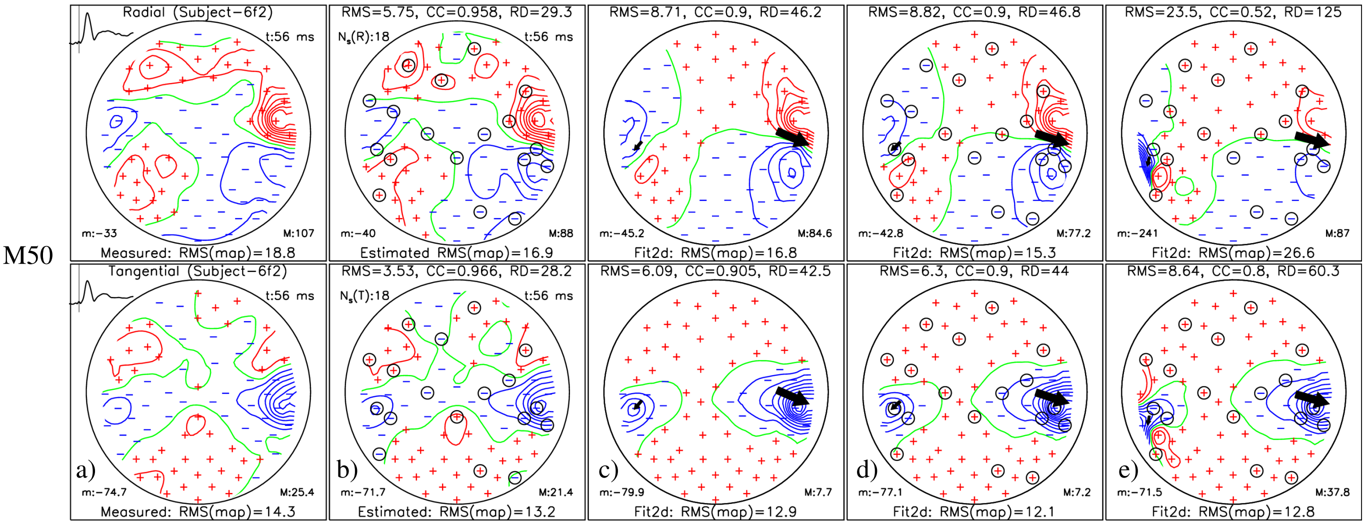

Fig. S.34: Subject-6f2: 18 selected sites using protocol **III**, fitting M50 with 2 dipoles:
a) Measured data, b) Estimated data map,
c) measured map fit: $\vec{r}_1$=(62.9,-2.6,21.6), $\vec{r}_2$=(-60.3,-8.8,22.6), $\vec{p}_1$=(-0.5,0.3,1.6), $\vec{p}_2$=(0.1,0.2,0.4),
d) estimated map fit: $\vec{r}_1$=(64,-3.3,21.2), $\vec{r}_2$=(-60.9,-9.2,21.4), $\vec{p}_1$=(-0.4,0.2,1.4), $\vec{p}_2$=(0.1,0.2,0.4),
reconstructed source errors: Δr_1=1.4, Δr_2=1.5, Δr_c=2, $\Delta\varphi_1$=2.89, $\Delta\varphi_2$=7.03,
e) selected chan. fit: $\vec{r}_1$=(63.2,-4.2,19.4), $\vec{r}_2$=(-80.6,-25.7,13.6), $\vec{p}_1$=(-0.4,0.2,1.4), $\vec{p}_2$=(0,0.3,0.2),
reconstructed source errors: Δr_1=2.8, Δr_2=28, Δr_c=28.1, $\Delta\varphi_1$=4.49, $\Delta\varphi_2$=28.5.

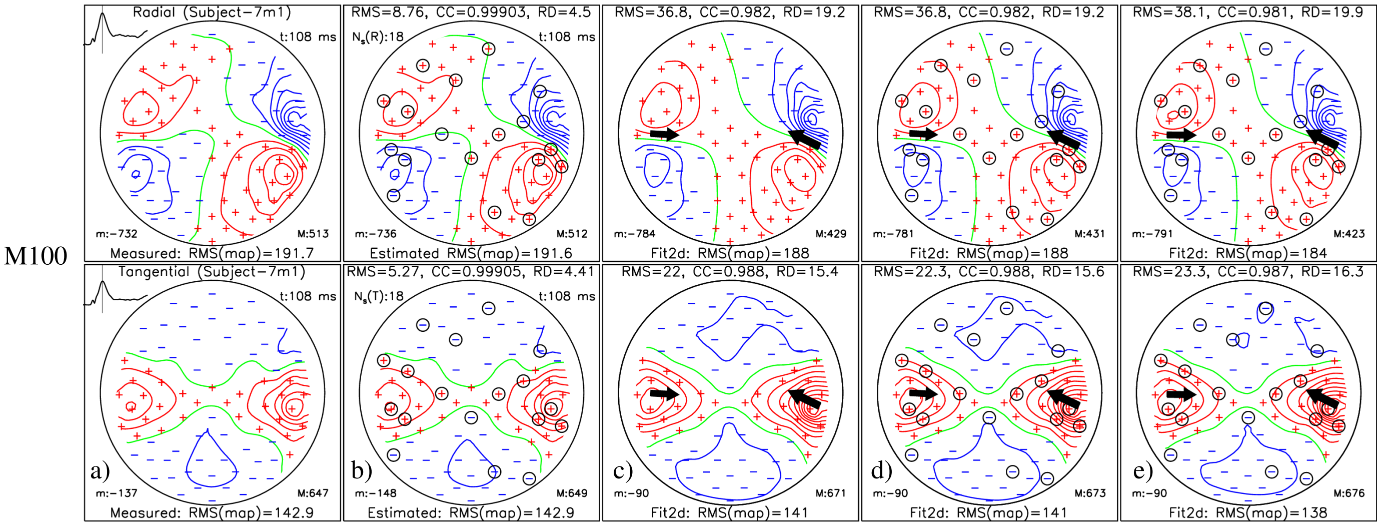

Fig. S.35: Subject-7m1: 18 selected sites using protocol **III**, fitting M100 with 2 dipoles:
a) Measured data, b) Estimated data map,
c) measured map fit: $\vec{r}_1$=(54.7,-2.8,22.8), $\vec{r}_2$=(-43.6,0.4,28.7), $\vec{p}_1$=(9.6,-6.9,-23.9), $\vec{p}_2$=(-11.9,0.8,-18),
d) estimated map fit: $\vec{r}_1$=(54.6,-2.9,22.6), $\vec{r}_2$=(-43.8,0.6,28.7), $\vec{p}_1$=(9.7,-6.7,-24.2), $\vec{p}_2$=(-11.7,0.7,-17.9),
reconstructed source errors: Δr_1=0.3, Δr_2=0.3, Δr_c=0.4, $\Delta\varphi_1$=0.592, $\Delta\varphi_2$=0.27,
e) selected chan. fit: $\vec{r}_1$=(56.6,-2.5,23.7), $\vec{r}_2$=(-45.5,0,28.5), $\vec{p}_1$=(8.4,-6.2,-20.8), $\vec{p}_2$=(-10.3,0.1,-16.4),
reconstructed source errors: Δr_1=2.1, Δr_2=1.9, Δr_c=2.8, $\Delta\varphi_1$=0.295, $\Delta\varphi_2$=2.09.

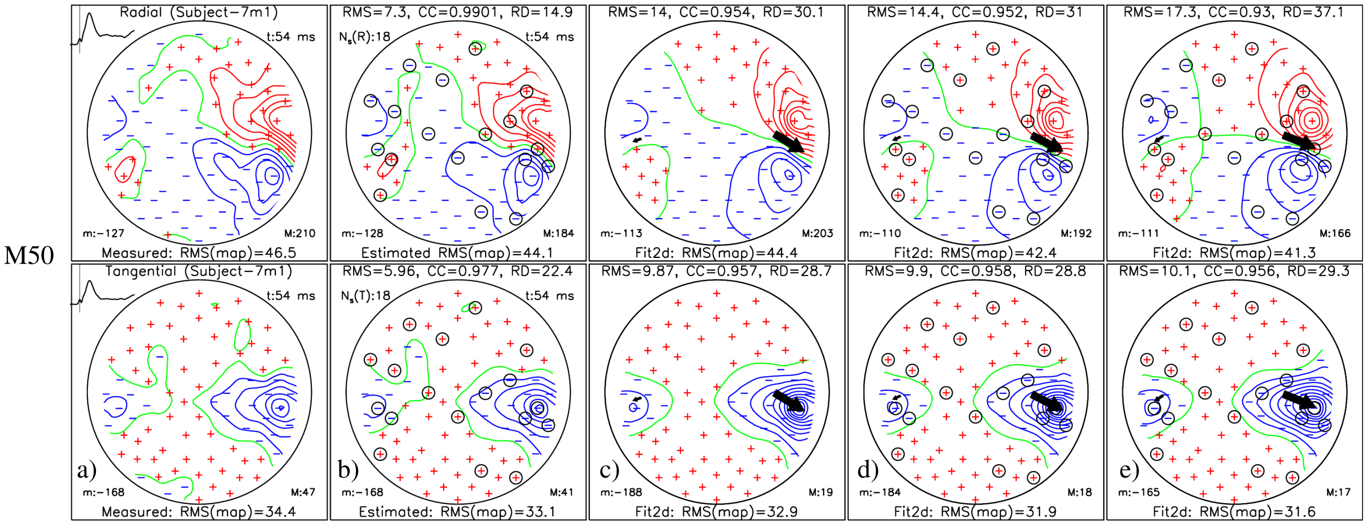

Fig. S.36: Subject-7m1: 18 selected sites using protocol **III**, fitting M50 with 2 dipoles:
a) Measured data, b) Estimated data map,
c) measured map fit: $\vec{r}_1$=(56.8,-6.2,24.6), $\vec{r}_2$=(-55.1,-3.8,19.1), $\vec{p}_1$=(-2.1,1.4,5.3), $\vec{p}_2$=(0.4,0.2,1.3),
d) estimated map fit: $\vec{r}_1$=(57.3,-6.6,25.9), $\vec{r}_2$=(-56.6,-2.8,20.6), $\vec{p}_1$=(-2.1,1.1,4.9), $\vec{p}_2$=(0.4,0.2,1.1),
reconstructed source errors: Δr_1=1.4, Δr_2=2.4, Δr_c=2.8, $\Delta\varphi_1$=2.71, $\Delta\varphi_2$=3.51,
e) selected chan. fit: $\vec{r}_1$=(55.7,-5.7,33.5), $\vec{r}_2$=(-54.7,-3.4,23.5), $\vec{p}_1$=(-2.6,0.8,4.4), $\vec{p}_2$=(0.5,0.4,1.3),
reconstructed source errors: Δr_1=8.9, Δr_2=4.4, Δr_c=9.9, $\Delta\varphi_1$=9.91, $\Delta\varphi_2$=9.28.

M100

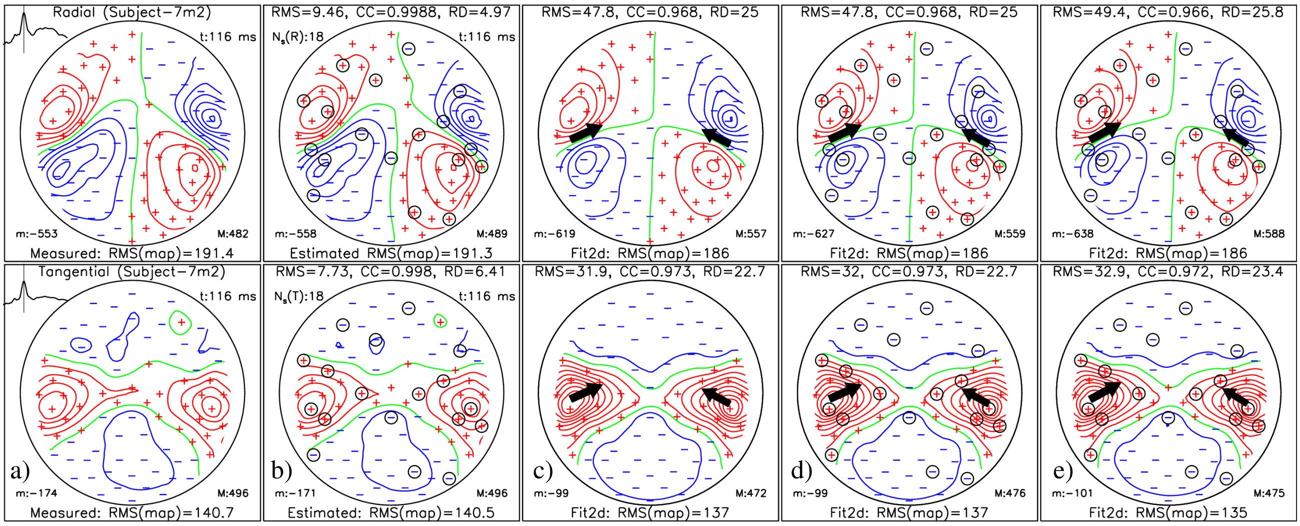

Fig. S.37: Subject-7m2: 18 selected sites using protocol **III**, fitting M100 with 2 dipoles:
a) Measured data, b) Estimated data map,
c) measured map fit: $\vec{r}_1$=(52.3,-1.6,31), $\vec{r}_2$=(-47,1.9,33), $\vec{p}_1$=(10.1,-7,-17.4), $\vec{p}_2$=(-13.1,-7.3,-18.2),
d) estimated map fit: $\vec{r}_1$=(52.7,-1.6,30.7), $\vec{r}_2$=(-47,2,32.8), $\vec{p}_1$=(9.8,-7,-17.2), $\vec{p}_2$=(-13.1,-7.3,-18.3),
reconstructed source errors: Δr_1=0.5, Δr_2=0.2, Δr_c=0.5, $\Delta\varphi_1$=0.697, $\Delta\varphi_2$=0.127,
e) selected chan. fit: $\vec{r}_1$=(54.2,-1.4,30.8), $\vec{r}_2$=(-47.5,2.5,34.2), $\vec{p}_1$=(8.6,-6.9,-15.4), $\vec{p}_2$=(-12.7,-8.4,-17),
reconstructed source errors: Δr_1=1.9, Δr_2=1.5, Δr_c=2.4, $\Delta\varphi_1$=2.34, $\Delta\varphi_2$=3.64.

M50

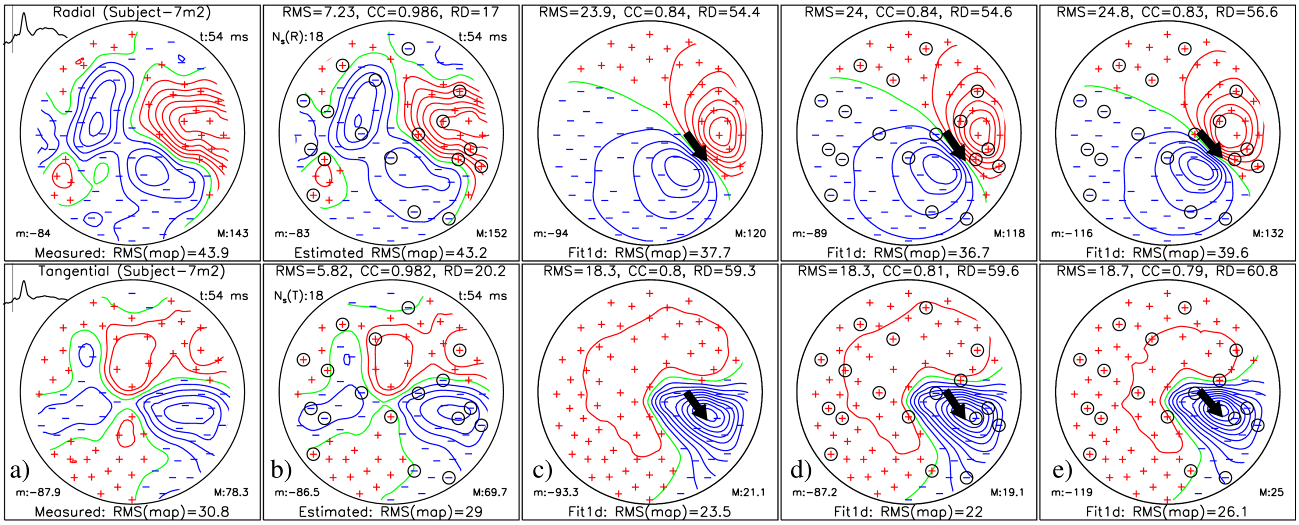

Fig. S.38: Subject-7m2: 18 selected sites using protocol **III**, fitting M50 with 1 dipole:
a) Measured data, b) Estimated data map,
c) measured map fit: $\vec{r}$=(43.9,-11.3,48.1), $\vec{p}$=(-2.6,3.1,3.1),
d) estimated map fit – source parameters: $\vec{r}$=(43.9,-10.4,48.3), $\vec{p}$=(-2.4,3.2,2.9),
reconstructed source errors: $\Delta\vec{r}$=(0,0.9,0.2), Δr=0.9, $\Delta\vec{p}$=(0.2,0.1,-0.2), Δp=0.4, $\Delta\varphi$=0.062,
e) selected chan. fit: $\vec{r}$=(44.4,-9.9,54.8), $\vec{p}$=(-2.7,2.6,2.7),
reconstructed source errors: $\Delta\vec{r}$=(0.5,1.3,6.7), Δr=6.8, $\Delta\vec{p}$=(-0.1,-0.4,-0.4), Δp=0.6, $\Delta\varphi$=0.088.

M100

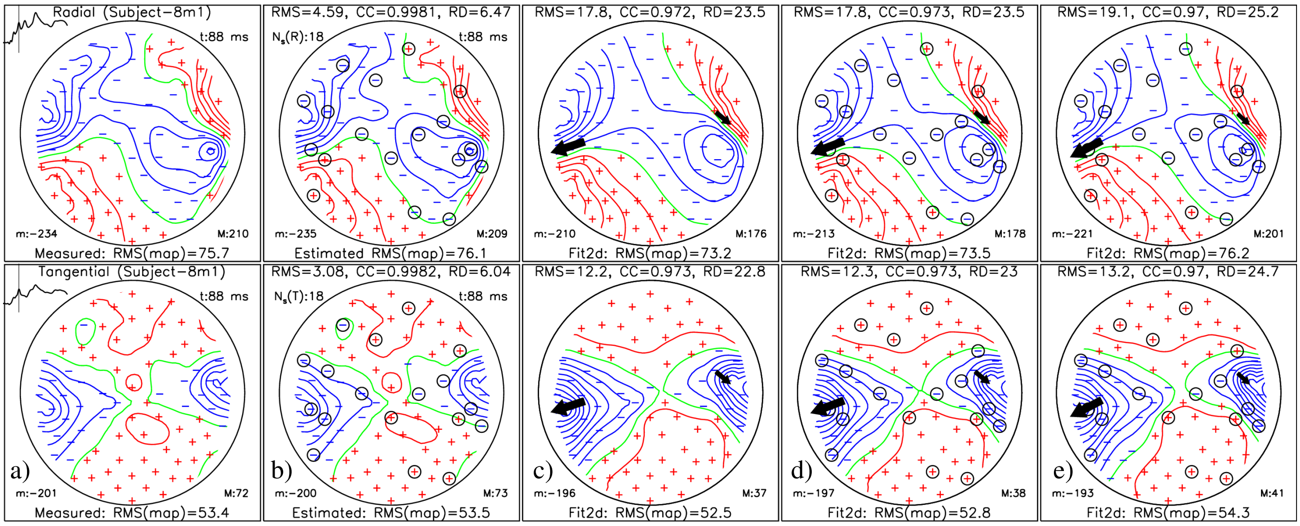

Fig. S.39: Subject-8m1: 18 selected sites using protocol **III**, fitting M100 with 2 dipoles:
a) Measured data, b) Estimated data map,
c) measured map fit: $\vec{r}_1$=(47.6,10.7,19.3), $\vec{r}_2$=(-38.1,-5.7,10.7), $\vec{p}_1$=(-4.8,6.2,8.4), $\vec{p}_2$=(5.5,2.5,21),
d) estimated map fit: $\vec{r}_1$=(47.8,10.9,19.3), $\vec{r}_2$=(-38.2,-5.8,11.1), $\vec{p}_1$=(-4.8,6.1,8.4), $\vec{p}_2$=(5.6,2.9,20.9),
reconstructed source errors: Δr_1=0.2, Δr_2=0.4, Δr_c=0.5, $\Delta\varphi_1$=0.32, $\Delta\varphi_2$=1.3,
e) selected chan. fit: $\vec{r}_1$=(49.9,10.3,18), $\vec{r}_2$=(-35.7,-6.1,10), $\vec{p}_1$=(-4.3,6.6,8.2), $\vec{p}_2$=(6,4.8,24.3),
reconstructed source errors: Δr_1=2.6, Δr_2=2.5, Δr_c=3.6, $\Delta\varphi_1$=3.05, $\Delta\varphi_2$=4.37.

M50

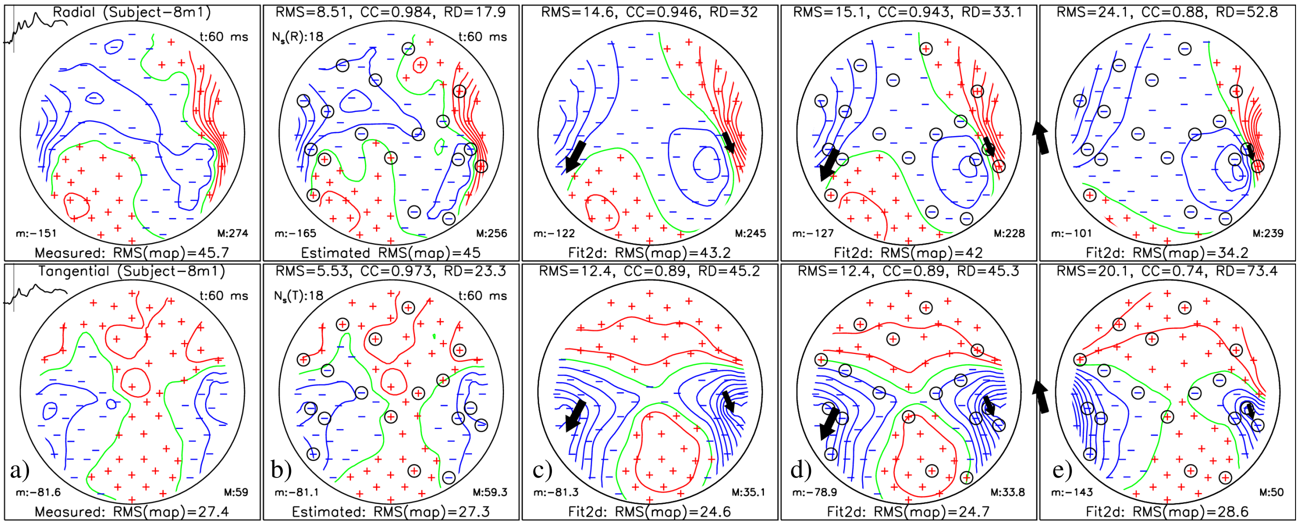

Fig. S.40: Subject-8m1: 18 selected sites using protocol **III**, fitting M50 with 2 dipoles:
a) Measured data, b) Estimated data map,
c) measured map fit: $\vec{r}_1$=(43.8,-4.5,13.2), $\vec{r}_2$=(-25.5,-7.9,10), $\vec{p}_1$=(-1.4,9.9,8.2), $\vec{p}_2$=(3.1,11,16.6),
d) estimated map fit: $\vec{r}_1$=(42.7,-6.4,11), $\vec{r}_2$=(-25,-9.8,6.2), $\vec{p}_1$=(-0.9,9.5,9.2), $\vec{p}_2$=(0.7,10.7,19.7),
reconstructed source errors: Δr_1=3.1, Δr_2=4.3, Δr_c=5.3, $\Delta\varphi_1$=5.1, $\Delta\varphi_2$=8.62,
e) selected chan. fit: $\vec{r}_1$=(59.4,-12.5,11.2), $\vec{r}_2$=(-76.7,-1.2,-80), $\vec{p}_1$=(0.2,3.2,2.7), $\vec{p}_2$=(-5.7,-5.3,5.5),
reconstructed source errors: Δr_1=17.6, Δr_2=103.7, Δr_c=105.2, $\Delta\varphi_1$=8.58, $\Delta\varphi_2$=85.1.

M100

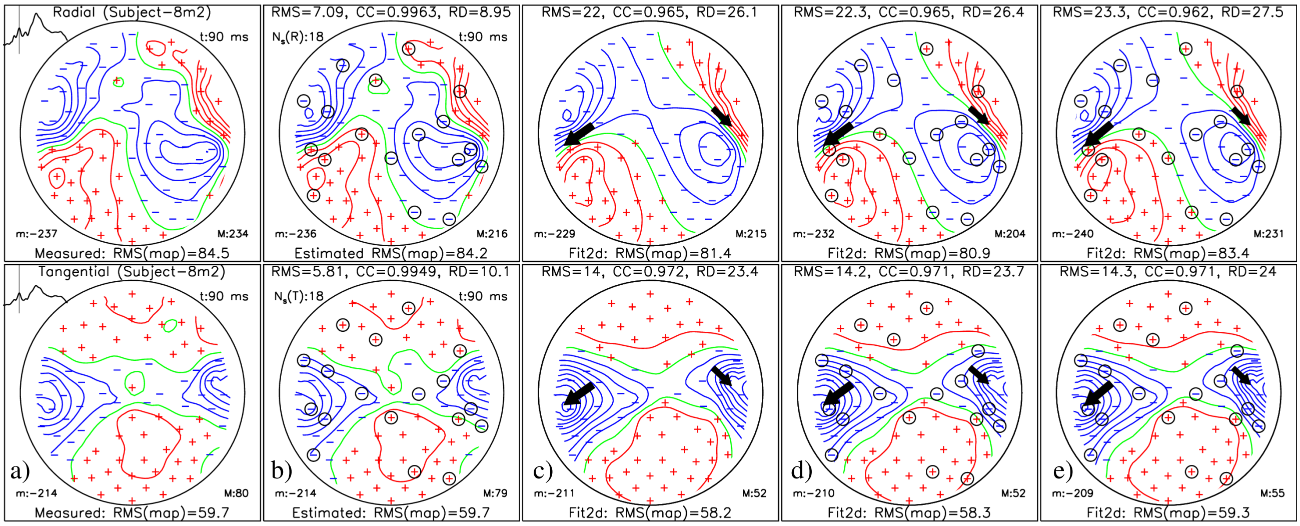

Fig. S.41: Subject-8m2: 18 selected sites using protocol **III**, fitting M100 with 2 dipoles:
a) Measured data, b) Estimated data map,
c) measured map fit: $\vec{r}_1$=(45.1,10.9,19), $\vec{r}_2$=(-38.5,-0.2,19.8), $\vec{p}_1$=(-6.7,9.8,10.2), $\vec{p}_2$=(9.3,6.7,18.2),
d) estimated map fit: $\vec{r}_1$=(44.3,11.7,20.4), $\vec{r}_2$=(-38.4,0,19.9), $\vec{p}_1$=(-7.1,9.1,10.3), $\vec{p}_2$=(9.5,7.3,18.2),
reconstructed source errors: Δr_1=1.8, Δr_2=0.2, Δr_c=1.8, $\Delta\varphi_1$=3.07, $\Delta\varphi_2$=1.38,
e) selected chan. fit: $\vec{r}_1$=(46.5,11.3,18.2), $\vec{r}_2$=(-37.4,0,20.5), $\vec{p}_1$=(-6.4,9.7,10.2), $\vec{p}_2$=(10.1,9,18.5),
reconstructed source errors: Δr_1=1.7, Δr_2=1.2, Δr_c=2.1, $\Delta\varphi_1$=0.957, $\Delta\varphi_2$=5.11.

M50

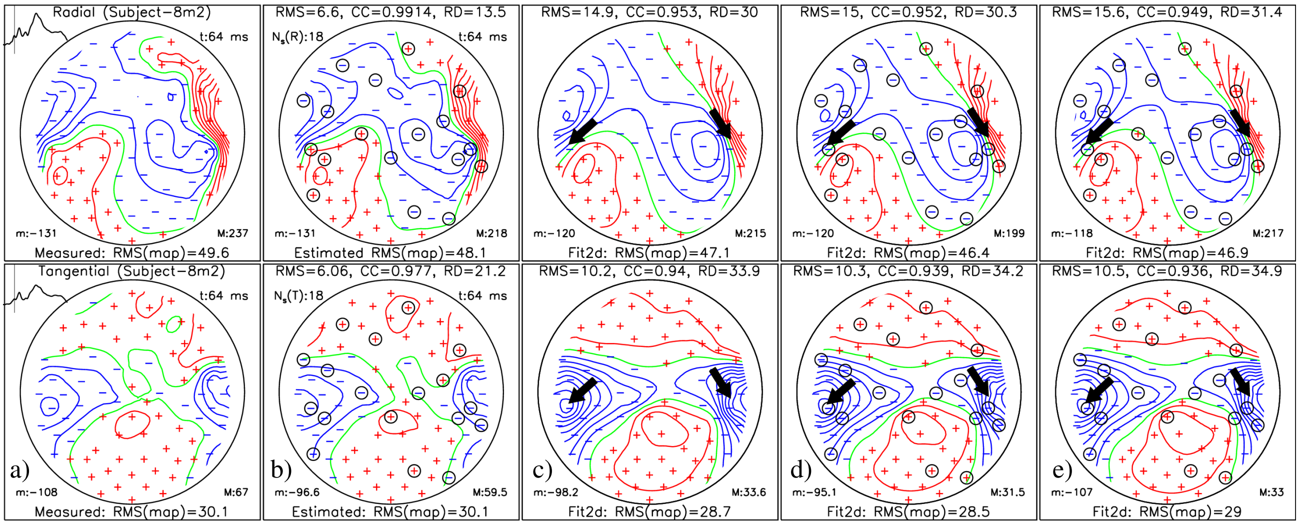

Fig. S.42: Subject-8m2: 18 selected sites using protocol **III**, fitting M50 with 2 dipoles:
a) Measured data, b) Estimated data map,
c) measured map fit: $\vec{r}_1$=(47,6.5,21.2), $\vec{r}_2$=(-37.8,1.2,22.8), $\vec{p}_1$=(-3.2,7.9,4.7), $\vec{p}_2$=(4.7,4.6,7.5),
d) estimated map fit: $\vec{r}_1$=(45.9,7.4,21.5), $\vec{r}_2$=(-37.5,0.7,22.2), $\vec{p}_1$=(-3.5,7.9,4.7), $\vec{p}_2$=(4.7,4.4,7.9),
reconstructed source errors: Δr_1=1.4, Δr_2=0.8, Δr_c=1.6, $\Delta\varphi_1$=1.37, $\Delta\varphi_2$=1.66,
e) selected chan. fit: $\vec{r}_1$=(46.8,6.9,18.9), $\vec{r}_2$=(-36.1,1.4,20.9), $\vec{p}_1$=(-3.3,8.4,5.1), $\vec{p}_2$=(5.2,5.5,8.6),
reconstructed source errors: Δr_1=2.3, Δr_2=2.5, Δr_c=3.4, $\Delta\varphi_1$=0.984, $\Delta\varphi_2$=1.69.

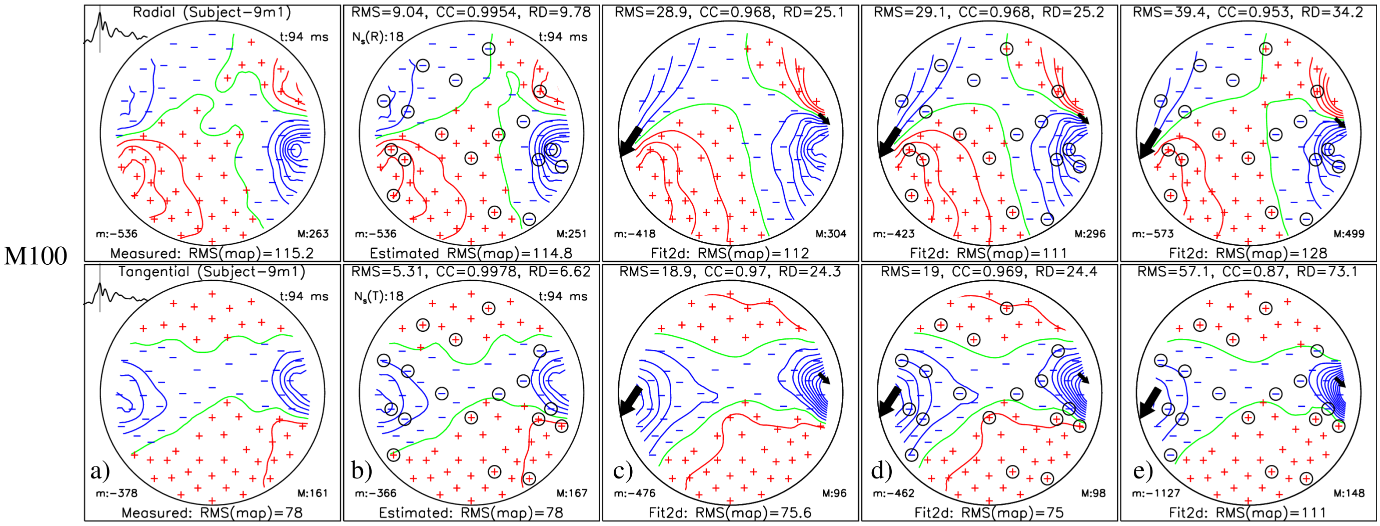

Fig. S.43: Subject-9m1: 18 selected sites using protocol **III**, fitting M100 with 2 dipoles:
a) Measured data, b) Estimated data map,
c) measured map fit: $\vec{r}_1$=(57.9,9.6,-1.2), $\vec{r}_2$=(-35,-2.6,-2.7), $\vec{p}_1$=(-1.1,8.6,13.6), $\vec{p}_2$=(-4,18.7,33.6),
d) estimated map fit: $\vec{r}_1$=(57.8,9.7,-1.6), $\vec{r}_2$=(-34.1,-2.8,-2.7), $\vec{p}_1$=(-1,8.4,13.7), $\vec{p}_2$=(-4.4,19.5,35.3),
reconstructed source errors: Δr_1=0.5, Δr_2=0.9, Δr_c=1, $\Delta\varphi_1$=0.836, $\Delta\varphi_2$=0.291,
e) selected chan. fit: $\vec{r}_1$=(68.5,8.8,2.3), $\vec{r}_2$=(-40.2,-3.7,-2.1), $\vec{p}_1$=(-1,5.3,9.5), $\vec{p}_2$=(-2.6,14.8,24.7),
reconstructed source errors: Δr_1=11.2, Δr_2=5.4, Δr_c=12.4, $\Delta\varphi_1$=3.14, $\Delta\varphi_2$=1.99.

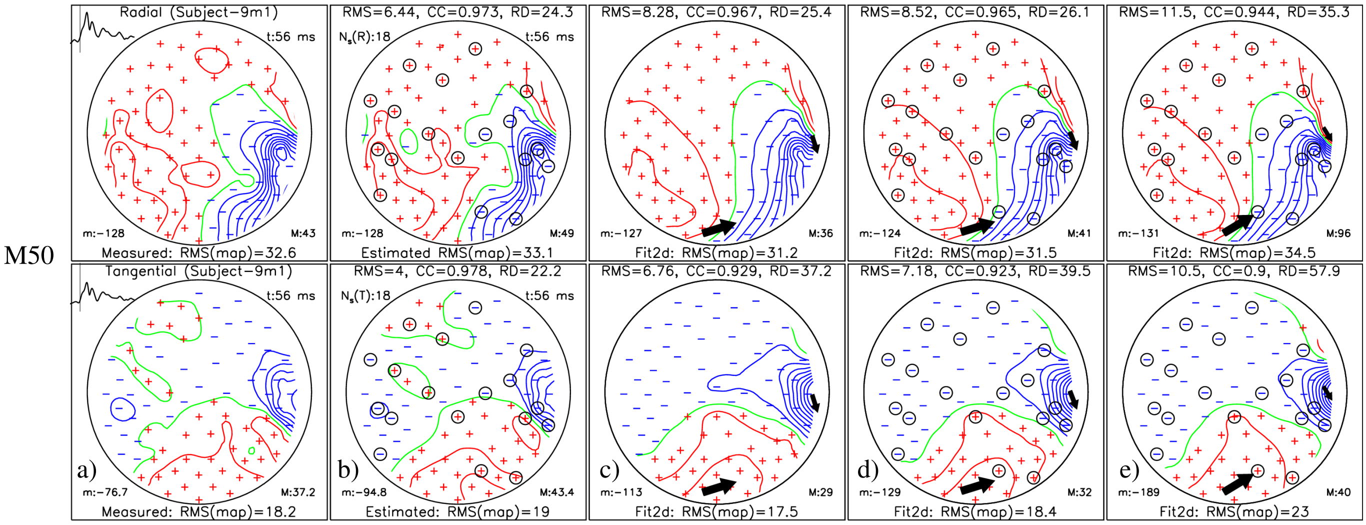

Fig. S.44: Subject-9m1: 18 selected sites using protocol **III**, fitting M50 with 2 dipoles:
a) Measured data, b) Estimated data map,
c) measured map fit: $\vec{r}_1$=(62.5,-6.1,-5.7), $\vec{r}_2$=(1.8,-43.5,2), $\vec{p}_1$=(0.5,2.7,2.1), $\vec{p}_2$=(-4.9,-0.4,-4.7),
d) estimated map fit: $\vec{r}_1$=(62.9,-3.9,-4.9), $\vec{r}_2$=(1.9,-48.7,3.5), $\vec{p}_1$=(0.3,2.5,2.2), $\vec{p}_2$=(-4.5,-0.5,-4.2),
reconstructed source errors: Δr_1=2.3, Δr_2=5.4, Δr_c=5.9, $\Delta\varphi_1$=3.6, $\Delta\varphi_2$=1.58,
e) selected chan. fit: $\vec{r}_1$=(66.4,0.3,1.5), $\vec{r}_2$=(3.1,-51.5,6.4), $\vec{p}_1$=(-0.1,1.7,2), $\vec{p}_2$=(-3.4,-0.8,-4.8),
reconstructed source errors: Δr_1=10.4, Δr_2=9.2, Δr_c=13.9, $\Delta\varphi_1$=15.5, $\Delta\varphi_2$=11.1.

M100

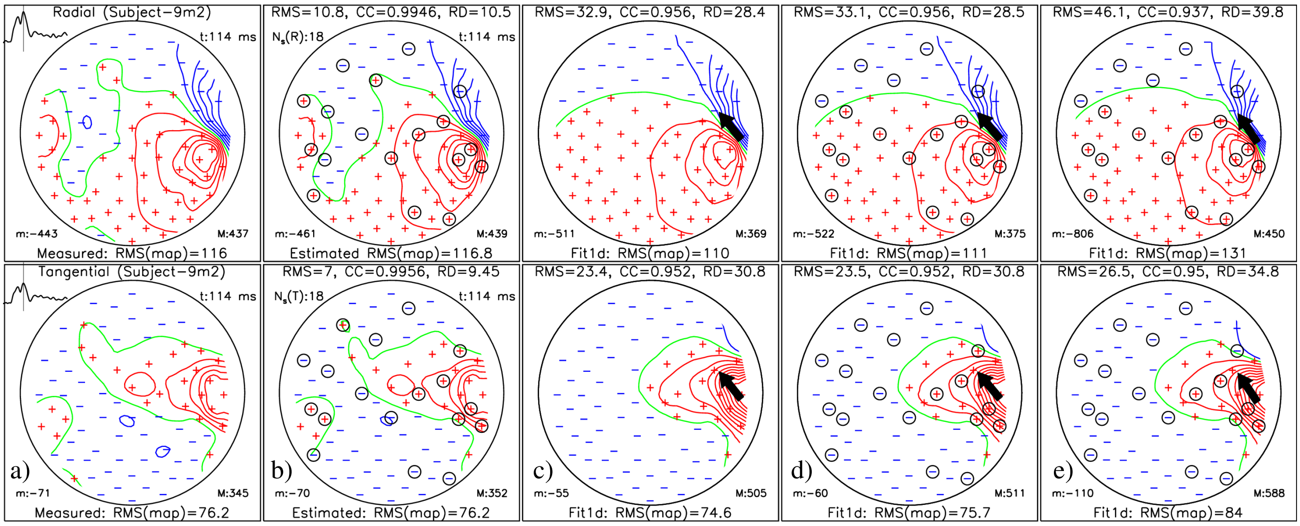

Fig. S.45: Subject-9m2: 36 selected channels using protocol **III**, fitting M100 with 1 dipole:
a) Measured data, b) Estimated data map,
c) measured map fit: $\vec{r}$=(55.4,6.2,15.7), $\vec{p}$=(5.2,-10.4,-14.1),
d) estimated map fit – source parameters: $\vec{r}$=(56,6.3,16.3), $\vec{p}$=(5.1,-9.9,-13.8),
reconstructed source errors: $\Delta\vec{r}$=(0.6,0.1,0.6), Δr=0.9, $\Delta\vec{p}$=(0,0.5,0.3), Δp=0.6, $\Delta\varphi$=0.014,
e) selected chan. fit: $\vec{r}$=(59.6,5,17.7), $\vec{p}$=(4.6,-10.7,-12.5),
reconstructed source errors: $\Delta\vec{r}$=(4.2,-1.2,2.1), Δr=4.8, $\Delta\vec{p}$=(-0.5,-0.3,1.6), Δp=1.8, $\Delta\varphi$=0.072.

M50

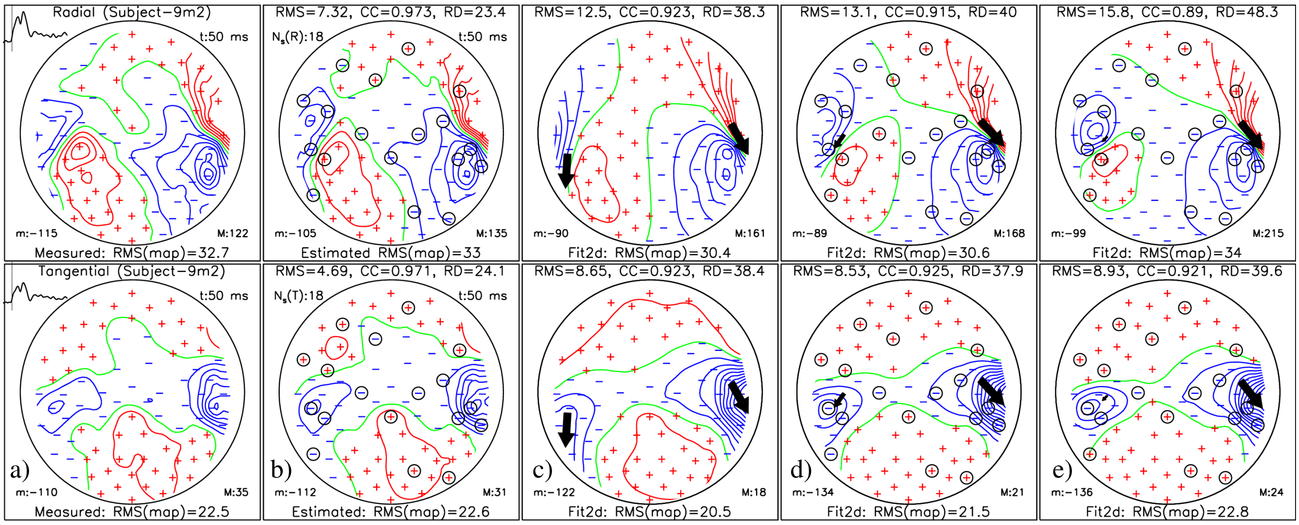

Fig. S.46: Subject-9m2: 18 selected sites using protocol **III**, fitting M50 with 2 dipoles:
a) Measured data, b) Estimated data map,
c) measured map fit: $\vec{r}_1$=(52.7,-2.5,4.6), $\vec{r}_2$=(-36.4,-16.1,5), $\vec{p}_1$=(-0.3,4.8,5.6), $\vec{p}_2$=(-1.7,5.3,4.8),
d) estimated map fit: $\vec{r}_1$=(57.2,0.9,12.4), $\vec{r}_2$=(-49.5,-4.9,27.9), $\vec{p}_1$=(-0.9,2.3,3.9), $\vec{p}_2$=(0.8,1.4,1.6),
reconstructed source errors: Δr_1=9.6, Δr_2=28.7, Δr_c=30.3, $\Delta\varphi_1$=13.6, $\Delta\varphi_2$=34,
e) selected chan. fit: $\vec{r}_1$=(58.6,-0.7,12.1), $\vec{r}_2$=(-58.8,-4.2,43), $\vec{p}_1$=(-0.8,2.6,3.9), $\vec{p}_2$=(0.5,0.4,0.7),
reconstructed source errors: Δr_1=9.7, Δr_2=45.7, Δr_c=46.7, $\Delta\varphi_1$=10.4, $\Delta\varphi_2$=45.3.

S.3 Localizations of M100 and M50 for all measurements using data from the right hemisphere only

Comparisons of M100 and M50 source localization for all cases using data from the right hemisphere only. First we applied SSA (protocol **III**) to find the first 9 optimal measuring sites out of 43 that covers the right hemisphere. Then we localized M100 and M50 with a single dipole source from the measured MFM, the estimated MFM and from the selected channels only. All results are displayed in the following figures. In figures's captions, all coordinates and localization errors are expressed in units of mm, dipole moments in μAm, and dipole orientation errors with angular degrees.

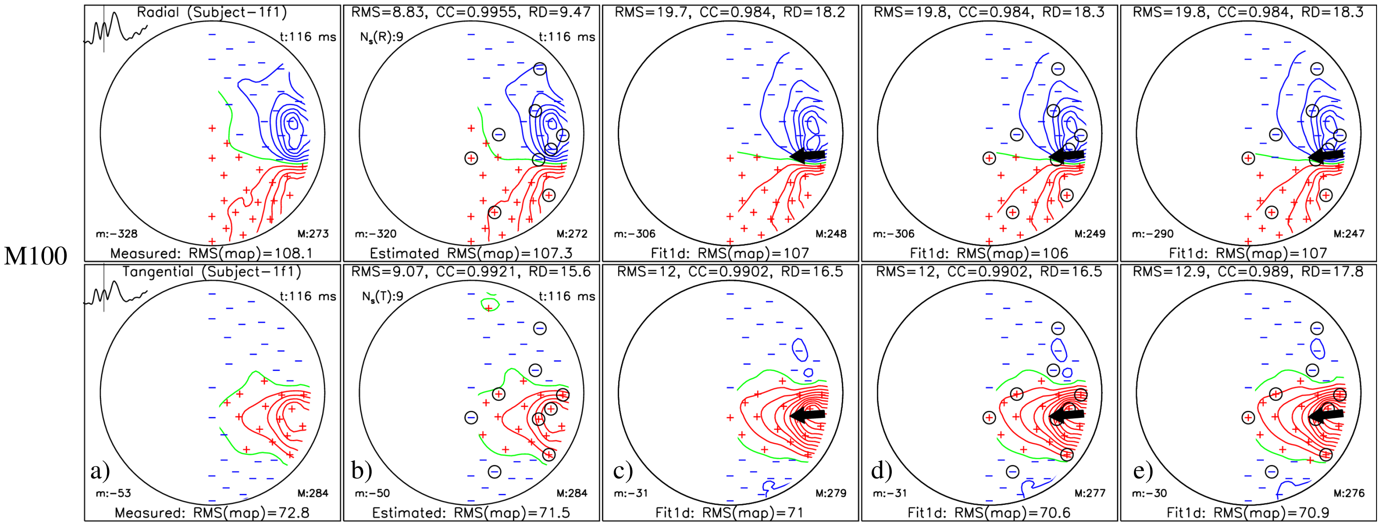

Fig. S.47: Subject-1f1: 18 selected channels using protocol **III** on right hemisphere only, fitting M100 with 1 dipole:
a) Measured data, b) Estimated data map,
c) measured map fit: $\vec{r}$=(59.6,-15.6,18.3), $\vec{p}$=(2.9,0.9,-8.6),
d) estimated map fit – source parameters: $\vec{r}$=(59.7,-15.6,18.2), $\vec{p}$=(2.8,0.9,-8.5),
reconstructed source errors: $\Delta\vec{r}$=(0.1,-0.1,-0.1), Δr=0.2, $\Delta\vec{p}$=(0,0.1,0.1), Δp=0.1, $\Delta\varphi$=0.0084,
e) selected chan. fit: $\vec{r}$=(58.5,-15.3,17.5), $\vec{p}$=(3,1.2,-9.2),
reconstructed source errors: $\Delta\vec{r}$=(-1.1,0.3,-0.8), Δr=1.4, $\Delta\vec{p}$=(0.2,0.3,-0.5), Δp=0.6, $\Delta\varphi$=0.026.

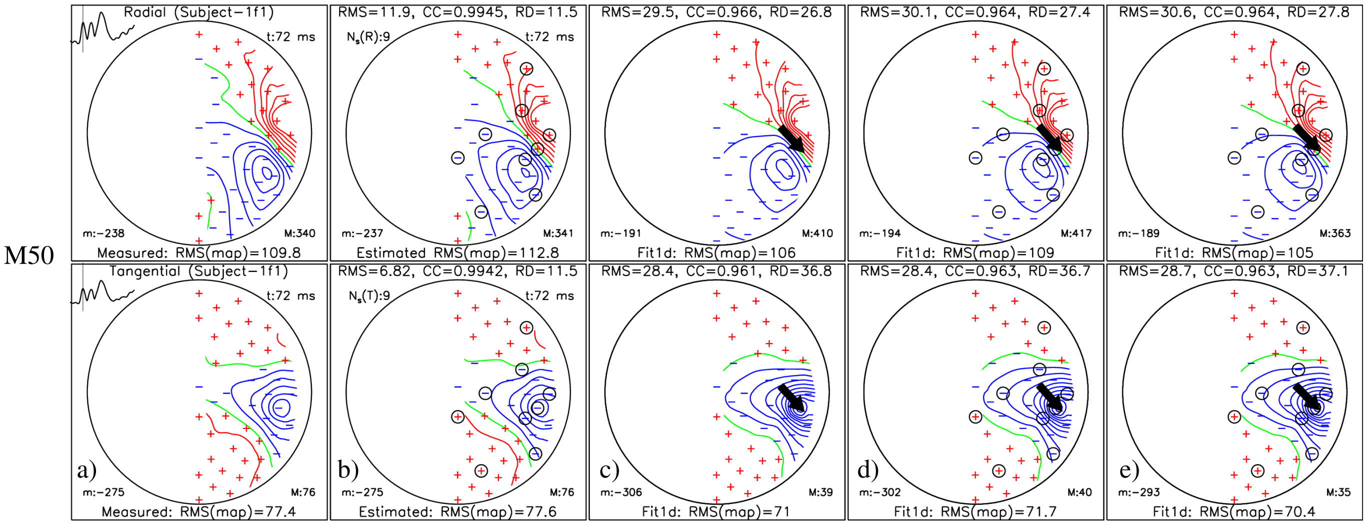

Fig. S.48: Subject-1f1: 18 selected channels using protocol **III** on right hemisphere only, fitting M50 with 1 dipole:
a) Measured data, b) Estimated data map,
c) measured map fit: $\vec{r}$=(56.6,-3.7,21.3), $\vec{p}$=(-3.1,5.2,9.2),
d) estimated map fit – source parameters: $\vec{r}$=(55.6,-3.1,21.6), $\vec{p}$=(-3.4,6,9.7),
reconstructed source errors: $\Delta\vec{r}$=(-1,0.6,0.4), Δr=1.2, $\Delta\vec{p}$=(-0.3,0.8,0.5), Δp=1, $\Delta\varphi$=0.038,
e) selected chan. fit: $\vec{r}$=(55.2,-2.9,23.6), $\vec{p}$=(-3.7,5.2,9.3),
reconstructed source errors: $\Delta\vec{r}$=(-1.4,0.8,2.4), Δr=2.8, $\Delta\vec{p}$=(-0.6,0,0.1), Δp=0.6, $\Delta\varphi$=0.047.

M100

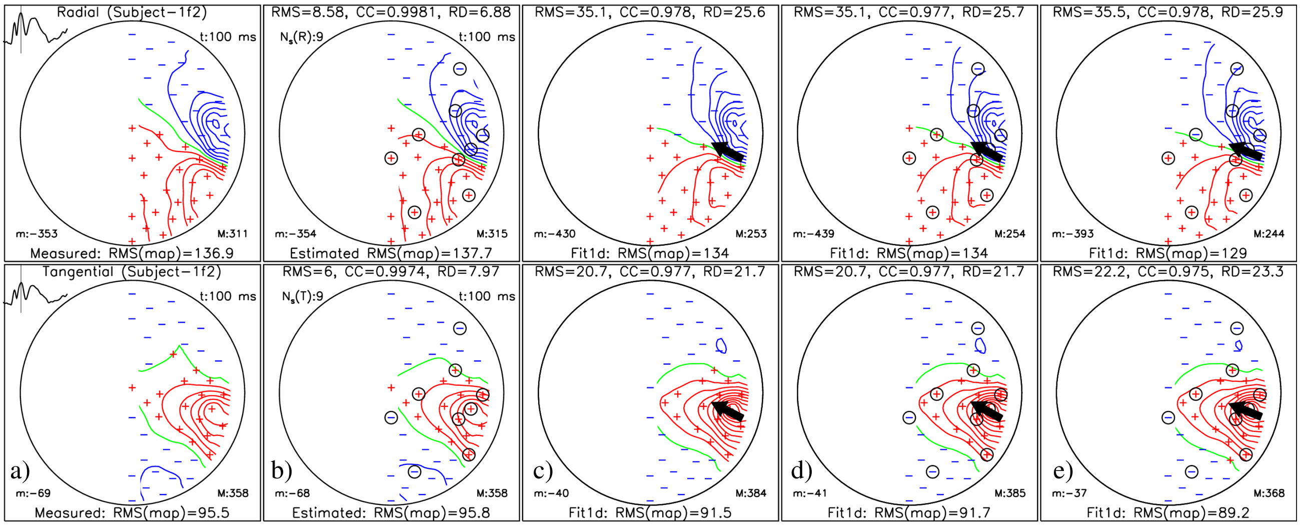

Fig. S.49: Subject-1f2: 18 selected channels using protocol **III** on right hemisphere only, fitting M100 with 1 dipole:
a) Measured data, b) Estimated data map,
c) measured map fit: $\vec{r}$=(57.3,-11.7,18.9), $\vec{p}$=(3.5,-2.7,-12.2),
d) estimated map fit – source parameters: $\vec{r}$=(57.3,-11.6,18.9), $\vec{p}$=(3.5,-2.9,-12.3),
reconstructed source errors: $\Delta\vec{r}$=(0,0.1,0), Δr=0.1, $\Delta\vec{p}$=(0,-0.2,-0.1), Δp=0.2, $\Delta\varphi$=0.016,
e) selected chan. fit: $\vec{r}$=(56.1,-11.4,18.4), $\vec{p}$=(3.7,-2.1,-12.7),
reconstructed source errors: $\Delta\vec{r}$=(-1.2,0.4,-0.5), Δr=1.3, $\Delta\vec{p}$=(0.3,0.6,-0.5), Δp=0.8, $\Delta\varphi$=0.053.

M50

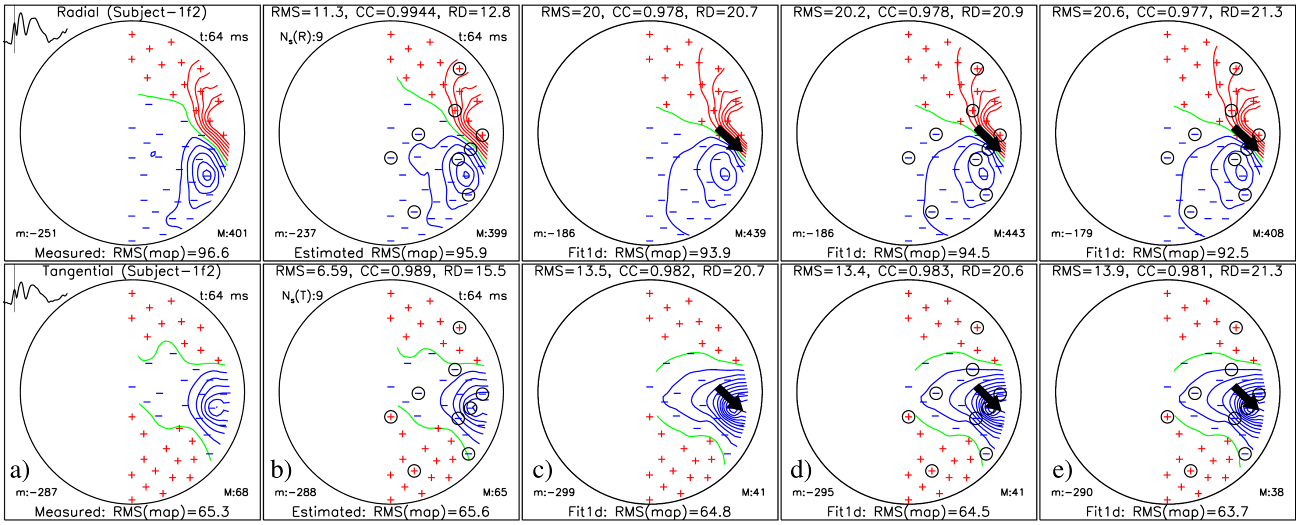

Fig. S.50: Subject-1f2: 18 selected channels using protocol **III** on right hemisphere only, fitting M50 with 1 dipole:
a) Measured data, b) Estimated data map,
c) measured map fit: $\vec{r}$=(61,-3.8,17), $\vec{p}$=(-1.8,3.3,7.3),
d) estimated map fit – source parameters: $\vec{r}$=(60.5,-3.6,17.1), $\vec{p}$=(-1.9,3.6,7.4),
reconstructed source errors: $\Delta\vec{r}$=(-0.4,0.1,0.2), Δr=0.5, $\Delta\vec{p}$=(-0.1,0.3,0.1), Δp=0.3, $\Delta\varphi$=0.025,
e) selected chan. fit: $\vec{r}$=(60.1,-3.4,18.5), $\vec{p}$=(-2,3.5,7.3),
reconstructed source errors: $\Delta\vec{r}$=(-0.9,0.4,1.6), Δr=1.8, $\Delta\vec{p}$=(-0.2,0.2,0), Δp=0.3, $\Delta\varphi$=0.031.

M100

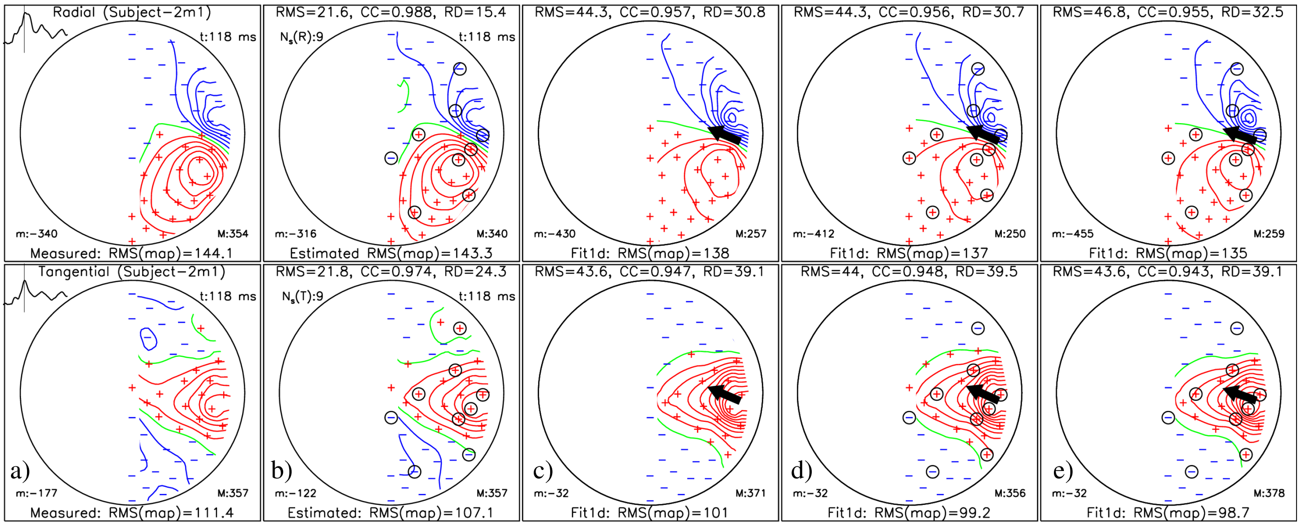

Fig. S.51: Subject-2m1: 18 selected channels using protocol **III** on right hemisphere only, fitting M100 with 1 dipole:
a) Measured data, b) Estimated data map,
c) measured map fit: $\vec{r}$=(53.3,0.3,22.3), $\vec{p}$=(6.2,-4,-14.8),
d) estimated map fit – source parameters: $\vec{r}$=(52.2,0.6,22.3), $\vec{p}$=(6.6,-4.4,-15.3),
reconstructed source errors: $\Delta\vec{r}$=(-1.1,0.4,0), Δr=1.1, $\Delta\vec{p}$=(0.4,-0.4,-0.5), Δp=0.7, $\Delta\varphi$=0.016,
e) selected chan. fit: $\vec{r}$=(55.8,0,26.2), $\vec{p}$=(5.6,-2.9,-12),
reconstructed source errors: $\Delta\vec{r}$=(2.5,-0.3,3.9), Δr=4.6, $\Delta\vec{p}$=(-0.6,1.1,2.8), Δp=3.1, $\Delta\varphi$=0.05.

M50

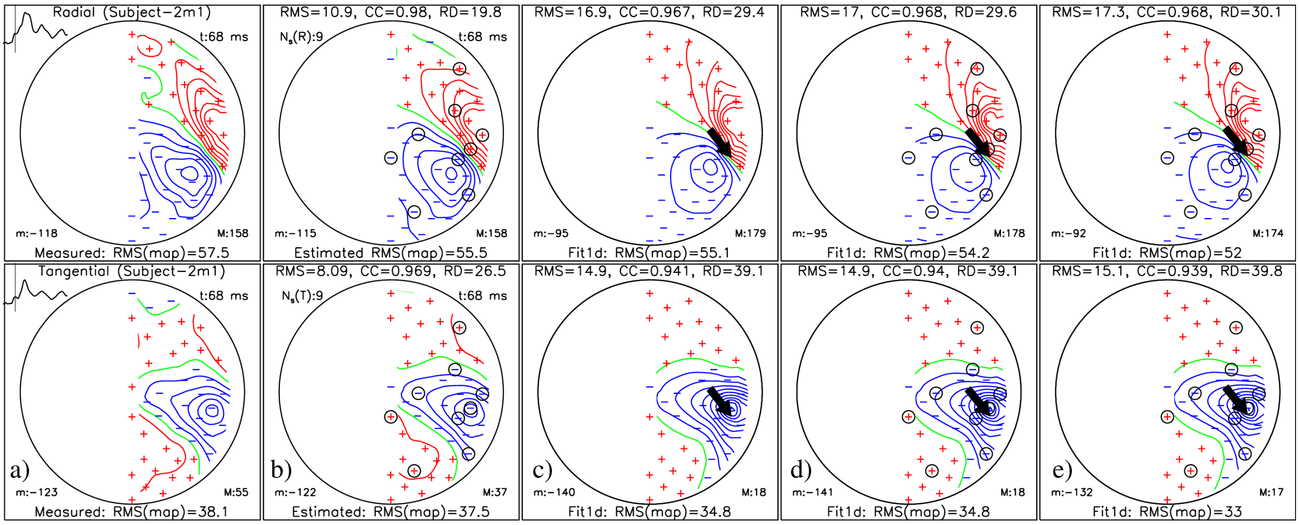

Fig. S.52: Subject-2m1: 18 selected channels using protocol **III** on right hemisphere only, fitting M50 with 1 dipole:
a) Measured data, b) Estimated data map,
c) measured map fit: $\vec{r}$=(53.6,-6.5,25.2), $\vec{p}$=(-1.9,3.3,4.8),
d) estimated map fit – source parameters: $\vec{r}$=(54.1,-6.5,26.1), $\vec{p}$=(-1.9,2.9,4.6),
reconstructed source errors: $\Delta\vec{r}$=(0.5,-0.1,0.9), Δr=1, $\Delta\vec{p}$=(0,-0.3,-0.2), Δp=0.4, $\Delta\varphi$=0.035,
e) selected chan. fit: $\vec{r}$=(53.6,-5.3,27.5), $\vec{p}$=(-1.9,3,4.3),
reconstructed source errors: $\Delta\vec{r}$=(0,1.2,2.3), Δr=2.6, $\Delta\vec{p}$=(0,-0.3,-0.5), Δp=0.6, $\Delta\varphi$=0.04.

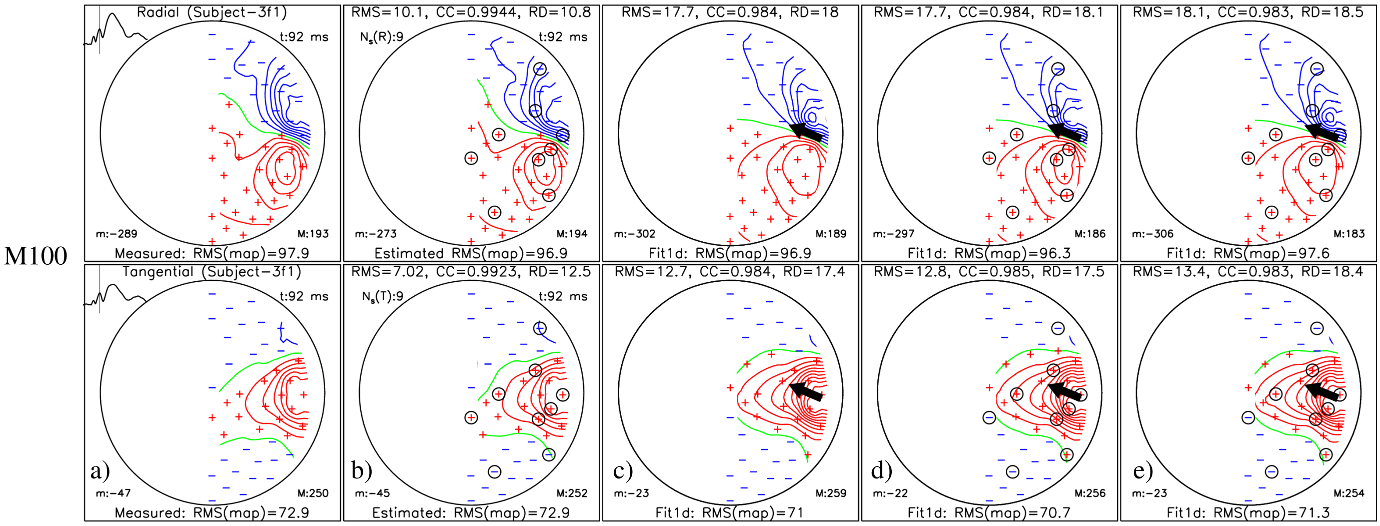

Fig. S.53: Subject-3f1: 18 selected channels using protocol **III** on right hemisphere only, fitting M100 with 1 dipole:
a) Measured data, b) Estimated data map,
c) measured map fit: $\vec{r}$=(55.1,2.2,21.4), $\vec{p}$=(3.9,-2.6,-9.7),
d) estimated map fit – source parameters: $\vec{r}$=(54.8,2.2,21.4), $\vec{p}$=(3.9,-2.6,-9.8),
reconstructed source errors: $\Delta\vec{r}$=(-0.3,0,0), Δr=0.3, $\Delta\vec{p}$=(0.1,0.1,-0.1), Δp=0.1, $\Delta\varphi$=0.0077,
e) selected chan. fit: $\vec{r}$=(54.3,2.1,23.4), $\vec{p}$=(4.3,-2.6,-9.7),
reconstructed source errors: $\Delta\vec{r}$=(-0.8,-0.1,1.9), Δr=2.1, $\Delta\vec{p}$=(0.4,0,-0.1), Δp=0.4, $\Delta\varphi$=0.034.

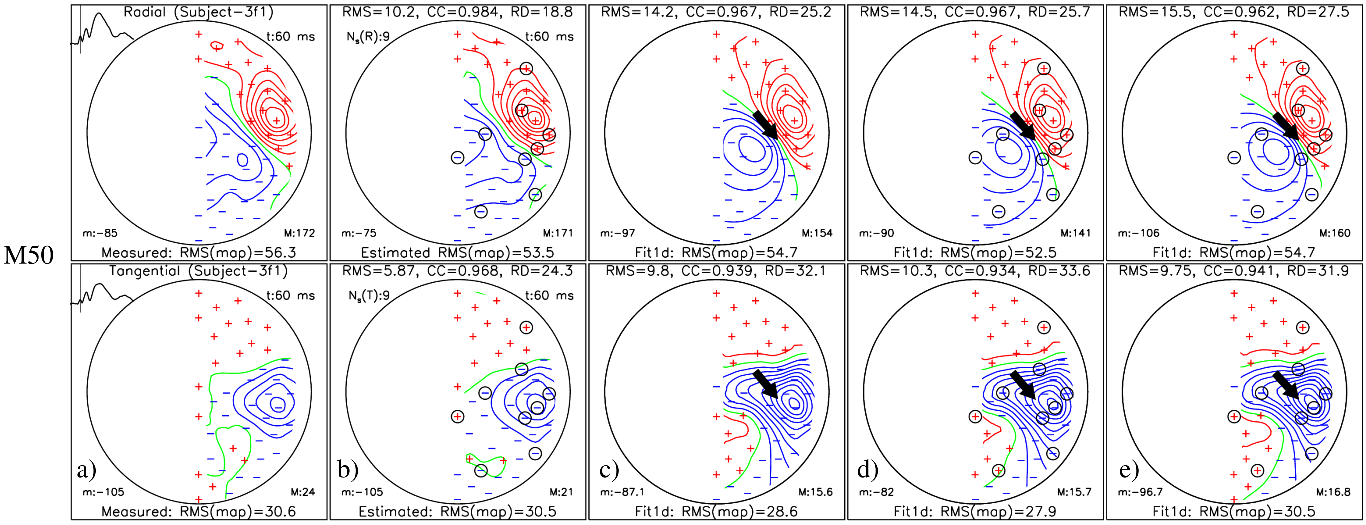

Fig. S.54: Subject-3f1: 18 selected channels using protocol **III** on right hemisphere only, fitting M50 with 1 dipole:
a) Measured data, b) Estimated data map,
c) measured map fit: $\vec{r}$=(44,7.2,44.2), $\vec{p}$=(-3.3,4.5,2.6),
d) estimated map fit – source parameters: $\vec{r}$=(42.4,6.4,44.7), $\vec{p}$=(-3.4,4.4,2.6),
reconstructed source errors: $\Delta\vec{r}$=(-1.5,-0.8,0.5), Δr=1.8, $\Delta\vec{p}$=(-0.1,-0.1,0), Δp=0.1, $\Delta\varphi$=0.023,
e) selected chan. fit: $\vec{r}$=(47,6.4,43.5), $\vec{p}$=(-3.1,3.8,2.8),
reconstructed source errors: $\Delta\vec{r}$=(3.1,-0.8,-0.8), Δr=3.3, $\Delta\vec{p}$=(0.2,-0.6,0.2), Δp=0.7, $\Delta\varphi$=0.096.

M100

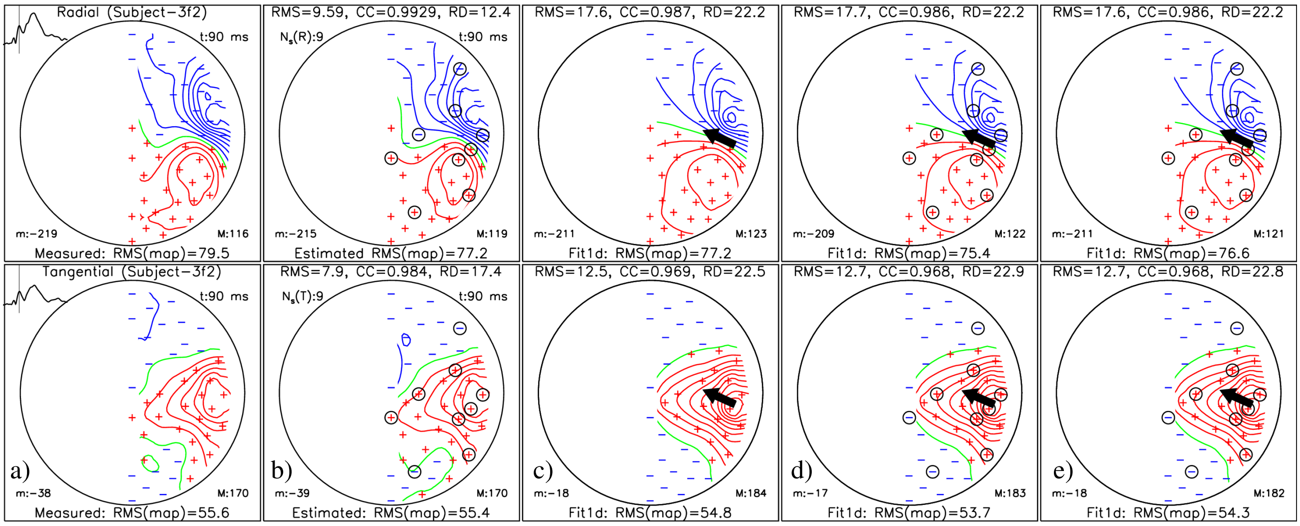

Fig. S.55: Subject-3f2: 18 selected channels using protocol **III** on right hemisphere only, fitting M100 with 1 dipole:
a) Measured data, b) Estimated data map,
c) measured map fit: $\vec{r}$=(46.3,-1.7,24.1), $\vec{p}$=(5.3,-3.2,-10.4),
d) estimated map fit – source parameters: $\vec{r}$=(47,-2.1,24.3), $\vec{p}$=(5,-2.9,-9.9),
reconstructed source errors: $\Delta\vec{r}$=(0.7,-0.3,0.2), Δr=0.8, $\Delta\vec{p}$=(-0.3,0.3,0.5), Δp=0.7, $\Delta\varphi$=0.014,
e) selected chan. fit: $\vec{r}$=(46.3,-1.9,25.2), $\vec{p}$=(5.3,-3.1,-10.1),
reconstructed source errors: $\Delta\vec{r}$=(0,-0.2,1), Δr=1.1, $\Delta\vec{p}$=(0,0.1,0.4), Δp=0.4, $\Delta\varphi$=0.016.

M50

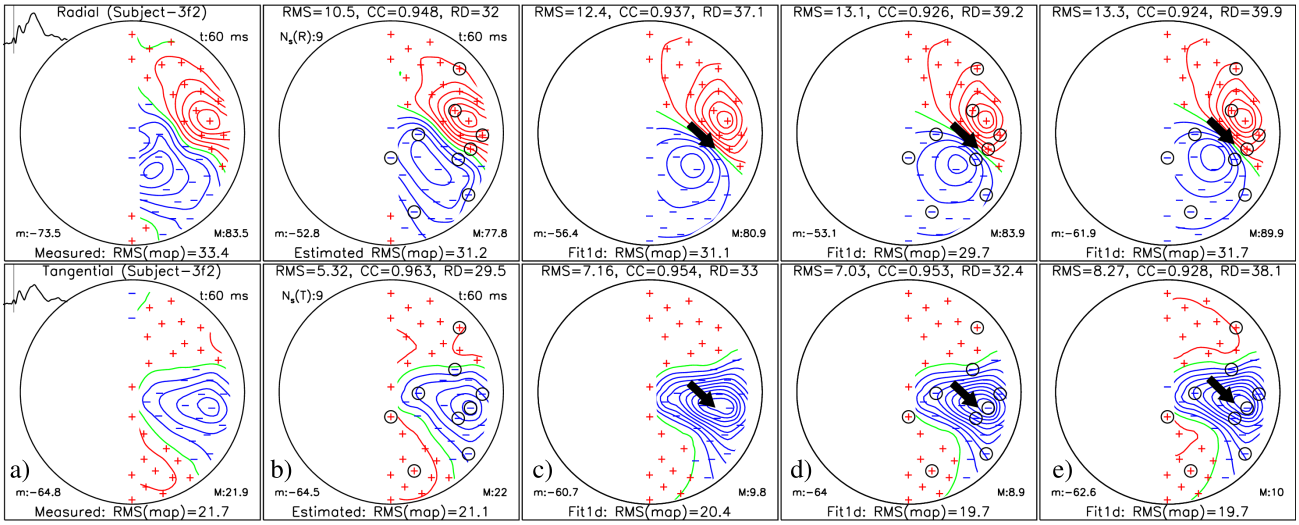

Fig. S.56: Subject-3f2: 18 selected channels using protocol **III** on right hemisphere only, fitting M50 with 1 dipole:
a) Measured data, b) Estimated data map,
c) measured map fit: $\vec{r}$=(46,-1.2,43.3), $\vec{p}$=(-2.1,1.7,2.2),
d) estimated map fit – source parameters: $\vec{r}$=(49.1,-1.3,40.1), $\vec{p}$=(-1.7,1.5,2.2),
reconstructed source errors: $\Delta\vec{r}$=(3.1,0,-3.2), Δr=4.4, $\Delta\vec{p}$=(0.3,-0.2,0), Δp=0.4, $\Delta\varphi$=0.068,
e) selected chan. fit: $\vec{r}$=(48.5,2.7,43), $\vec{p}$=(-1.8,1.8,2),
reconstructed source errors: $\Delta\vec{r}$=(2.5,3.9,-0.3), Δr=4.6, $\Delta\vec{p}$=(0.2,0.1,-0.3), Δp=0.4, $\Delta\varphi$=0.079.

M100

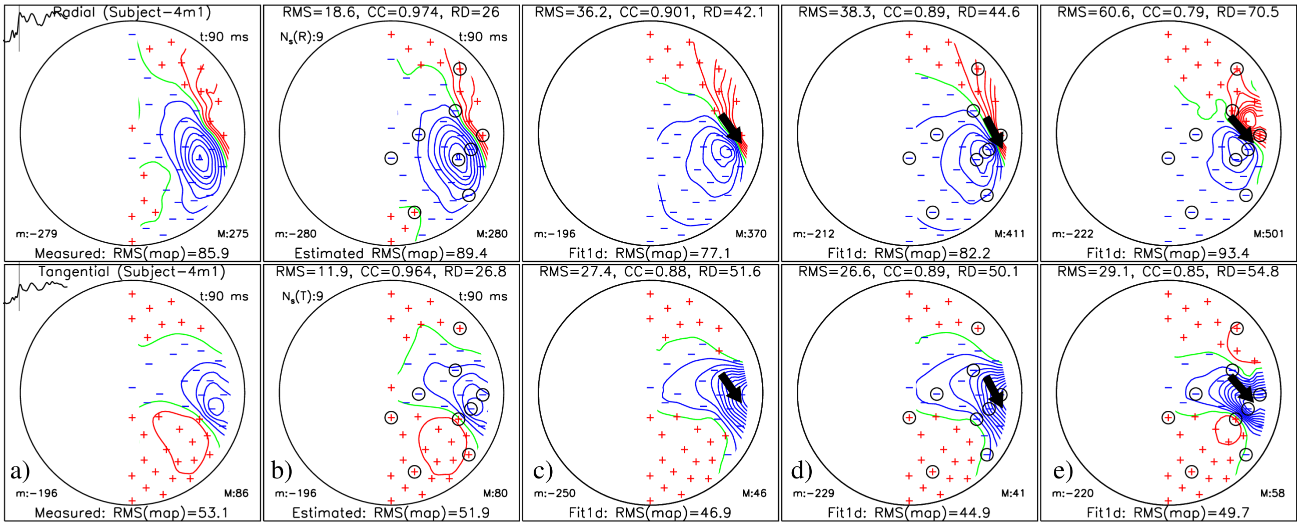

Fig. S.57: Subject-4m1: 18 selected channels using protocol **III** on right hemisphere only, fitting M100 with 1 dipole:
a) Measured data, b) Estimated data map,
c) measured map fit: $\vec{r}$=(58.4,4.3,15.5), $\vec{p}$=(-1.9,5.8,5.7),
d) estimated map fit – source parameters: $\vec{r}$=(56.2,1.4,10.7), $\vec{p}$=(-1.5,8.6,6.8),
reconstructed source errors: $\Delta\vec{r}$=(-2.2,-2.9,-4.8), Δr=6.1, $\Delta\vec{p}$=(0.4,2.8,1.1), Δp=3, $\Delta\varphi$=0.147,
e) selected chan. fit: $\vec{r}$=(69.6,4.9,29.1), $\vec{p}$=(-1.4,2.3,2.9),
reconstructed source errors: $\Delta\vec{r}$=(11.2,0.5,13.6), Δr=17.6, $\Delta\vec{p}$=(0.6,-3.5,-2.8), Δp=4.5, $\Delta\varphi$=0.162.

M50

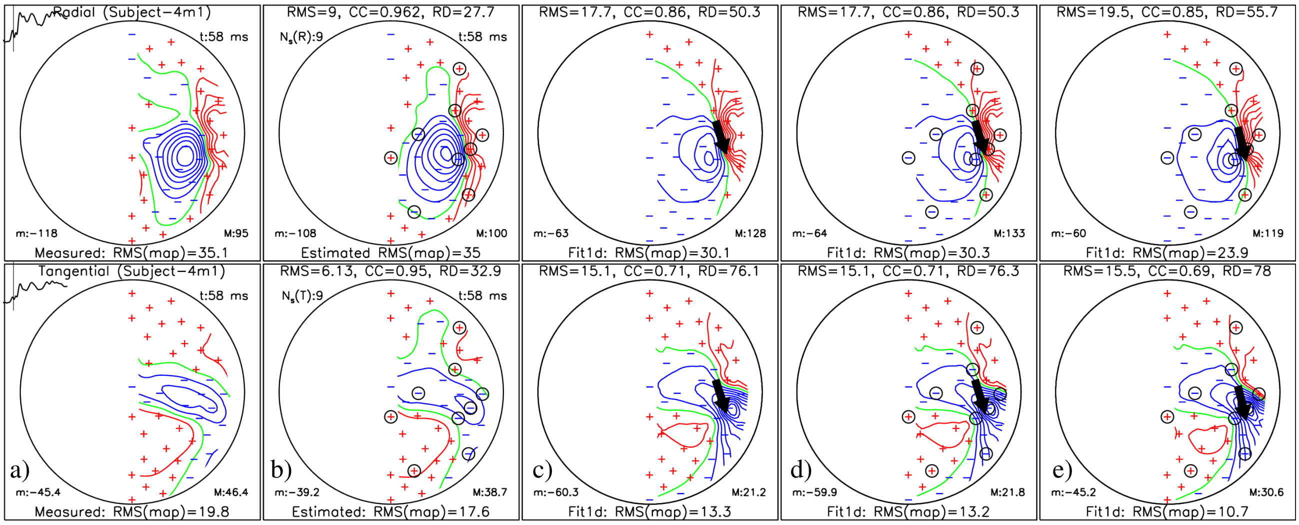

Fig. S.58: Subject-4m1: 18 selected channels using protocol **III** on right hemisphere only, fitting M50 with 1 dipole:
a) Measured data, b) Estimated data map,
c) measured map fit: $\vec{r}$=(60.5,-2.5,28.3), $\vec{p}$=(-0.3,2.2,0.9),
d) estimated map fit – source parameters: $\vec{r}$=(60.8,-2.5,28), $\vec{p}$=(-0.3,2.2,0.9),
reconstructed source errors: $\Delta\vec{r}$=(0.3,0,-0.3), Δr=0.4, $\Delta\vec{p}$=(0,0,0), Δp=0, $\Delta\varphi$=0.016,
e) selected chan. fit: $\vec{r}$=(63.8,-7.8,24.7), $\vec{p}$=(-0.1,1.6,0.7),
reconstructed source errors: $\Delta\vec{r}$=(3.3,-5.3,-3.5), Δr=7.2, $\Delta\vec{p}$=(0.3,-0.6,-0.2), Δp=0.7, $\Delta\varphi$=0.098.

M100

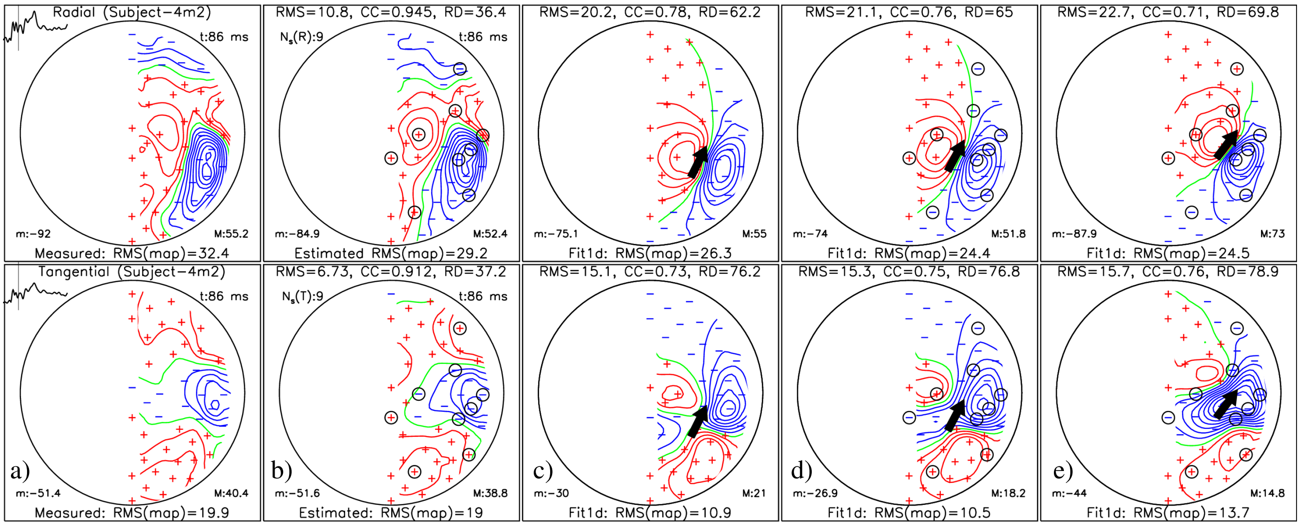

Fig. S.59: Subject-4m2: 18 selected channels using protocol **III** on right hemisphere only, fitting M100 with 1 dipole:
a) Measured data, b) Estimated data map,
c) measured map fit: $\vec{r}$=(49.3,-26.3,48.1), $\vec{p}$=(-0.8,-1.8,-0.1),
d) estimated map fit – source parameters: $\vec{r}$=(48.5,-20.8,52.2), $\vec{p}$=(-0.8,-1.7,0.1),
reconstructed source errors: $\Delta\vec{r}$=(-0.8,5.5,4.1), Δr=6.9, $\Delta\vec{p}$=(0,0.2,0.2), Δp=0.3, $\Delta\varphi$=0.112,
e) selected chan. fit: $\vec{r}$=(60.8,-9.3,45.9), $\vec{p}$=(-0.7,-1.1,0.7),
reconstructed source errors: $\Delta\vec{r}$=(11.5,17,-2.2), Δr=20.6, $\Delta\vec{p}$=(0.2,0.8,0.8), Δp=1.1, $\Delta\varphi$=0.567.

M50

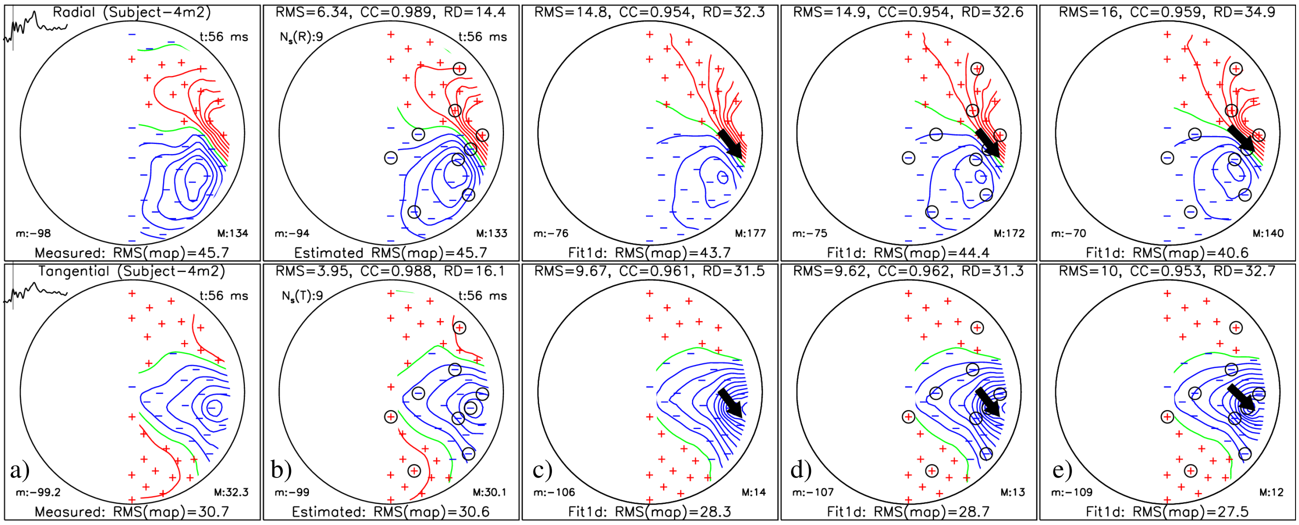

Fig. S.60: Subject-4m2: 18 selected channels using protocol **III** on right hemisphere only, fitting M50 with 1 dipole:
a) Measured data, b) Estimated data map,
c) measured map fit: $\vec{r}$=(50.4,-6.2,12.6), $\vec{p}$=(-1.1,3.4,5.9),
d) estimated map fit – source parameters: $\vec{r}$=(49.7,-5.6,13.8), $\vec{p}$=(-1.3,3.5,6),
reconstructed source errors: $\Delta\vec{r}$=(-0.7,0.6,1.2), Δr=1.5, $\Delta\vec{p}$=(-0.2,0.1,0.2), Δp=0.3, $\Delta\varphi$=0.027,
e) selected chan. fit: $\vec{r}$=(52.8,-3.2,20.7), $\vec{p}$=(-1.6,2.2,4.3),
reconstructed source errors: $\Delta\vec{r}$=(2.4,3,8.1), Δr=9, $\Delta\vec{p}$=(-0.5,-1.2,-1.6), Δp=2, $\Delta\varphi$=0.168.

M100

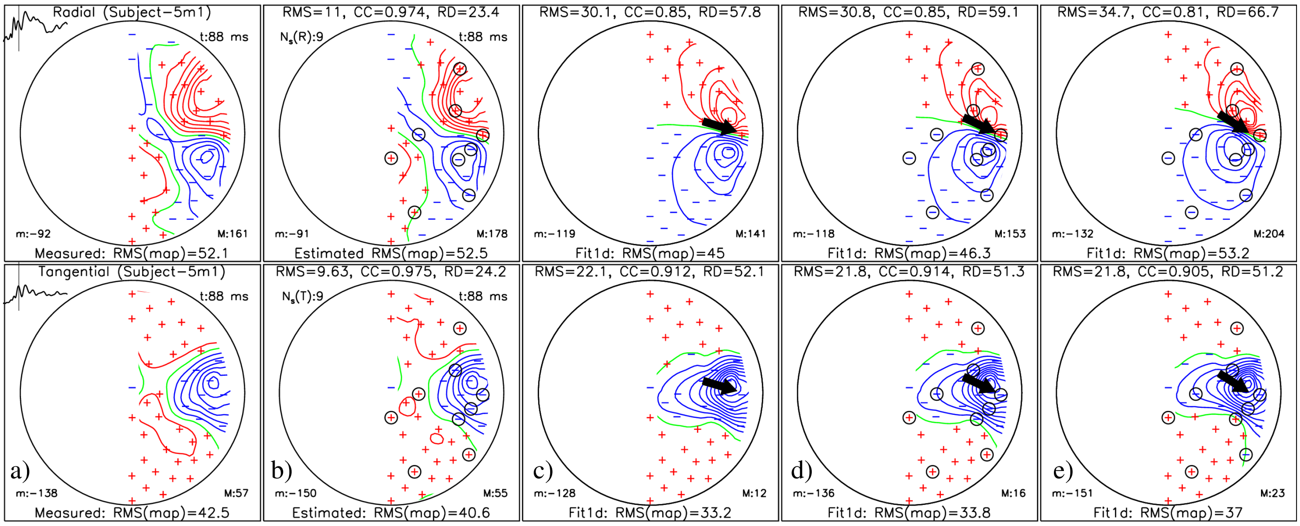

Fig. S.61: Subject-5m1: 18 selected channels using protocol **III** on right hemisphere only, fitting M100 with 1 dipole:
a) Measured data, b) Estimated data map,
c) measured map fit: $\vec{r}$=(59,7.2,29.2), $\vec{p}$=(-1.7,0.4,3.2),
d) estimated map fit – source parameters: $\vec{r}$=(59.1,8.4,28.6), $\vec{p}$=(-1.7,0.9,3.3),
reconstructed source errors: $\Delta\vec{r}$=(0.1,1.3,-0.7), Δr=1.4, $\Delta\vec{p}$=(0,0.4,0), Δp=0.4, $\Delta\varphi$=0.108,
e) selected chan. fit: $\vec{r}$=(60.7,10.7,35.5), $\vec{p}$=(-1.9,1.3,2.8),
reconstructed source errors: $\Delta\vec{r}$=(1.8,3.6,6.3), Δr=7.4, $\Delta\vec{p}$=(-0.2,0.8,-0.4), Δp=1, $\Delta\varphi$=0.266.

M50

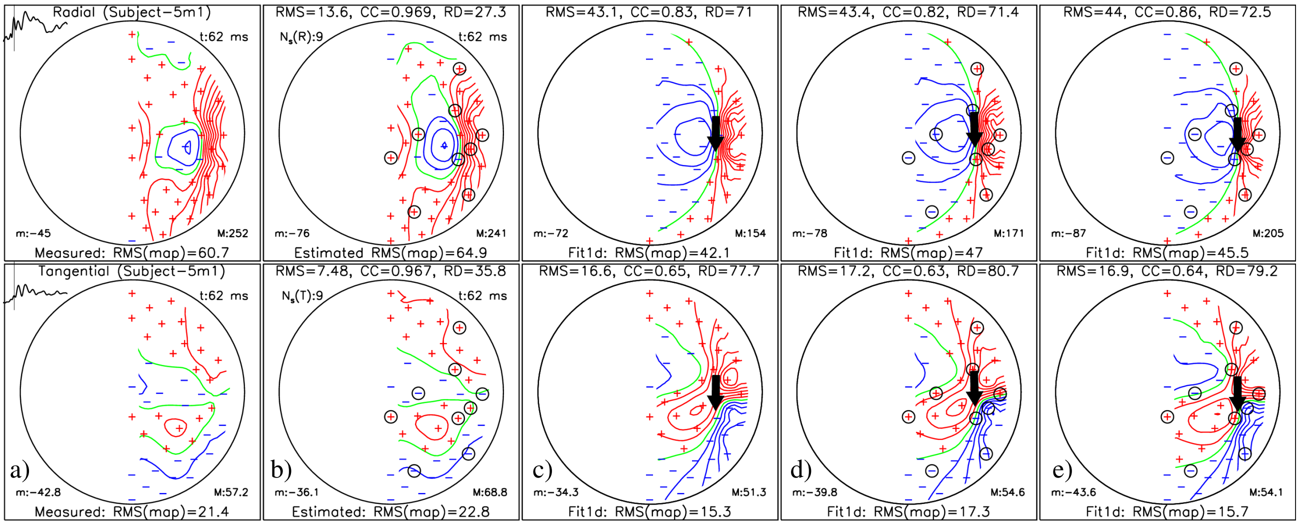

Fig. S.62: Subject-5m1: 18 selected channels using protocol **III** on right hemisphere only, fitting M50 with 1 dipole:
a) Measured data, b) Estimated data map,
c) measured map fit: $\vec{r}$=(53,0.8,31), $\vec{p}$=(0.3,4.5,-0.6),
d) estimated map fit – source parameters: $\vec{r}$=(53.1,4,31), $\vec{p}$=(0,4.9,-0.7),
reconstructed source errors: $\Delta\vec{r}$=(0.1,3.3,0), Δr=3.3, $\Delta\vec{p}$=(-0.2,0.4,-0.1), Δp=0.5, $\Delta\varphi$=0.054,
e) selected chan. fit: $\vec{r}$=(56.4,-0.2,27.6), $\vec{p}$=(0.2,4.6,-0.4),
reconstructed source errors: $\Delta\vec{r}$=(3.4,-1,-3.3), Δr=4.8, $\Delta\vec{p}$=(0,0.1,0.1), Δp=0.2, $\Delta\varphi$=0.033.

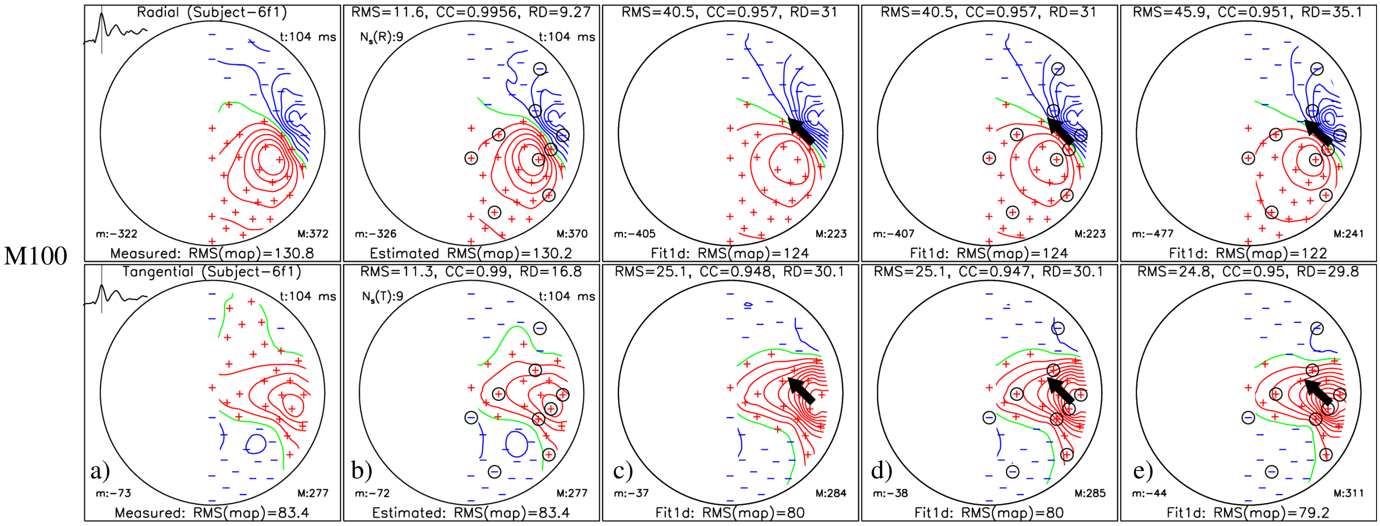

Fig. S.63: Subject-6f1: 18 selected channels using protocol **III** on right hemisphere only, fitting M100 with 1 dipole:
a) Measured data, b) Estimated data map,
c) measured map fit: $\vec{r}$=(51.7,3.2,25.3), $\vec{p}$=(6.1,-8.2,-11.5),
d) estimated map fit – source parameters: $\vec{r}$=(52,3.3,25.2), $\vec{p}$=(6,-8.2,-11.4),
reconstructed source errors: $\Delta\vec{r}$=(0.3,0,-0.1), Δr=0.3, $\Delta\vec{p}$=(-0.1,0.1,0.1), Δp=0.2, $\Delta\varphi$=0.0036,
e) selected chan. fit: $\vec{r}$=(57.2,2.5,29.5), $\vec{p}$=(4.5,-5.5,-8.3),
reconstructed source errors: $\Delta\vec{r}$=(5.4,-0.8,4.2), Δr=6.9, $\Delta\vec{p}$=(-1.6,2.7,3.2), Δp=4.5, $\Delta\varphi$=0.036.

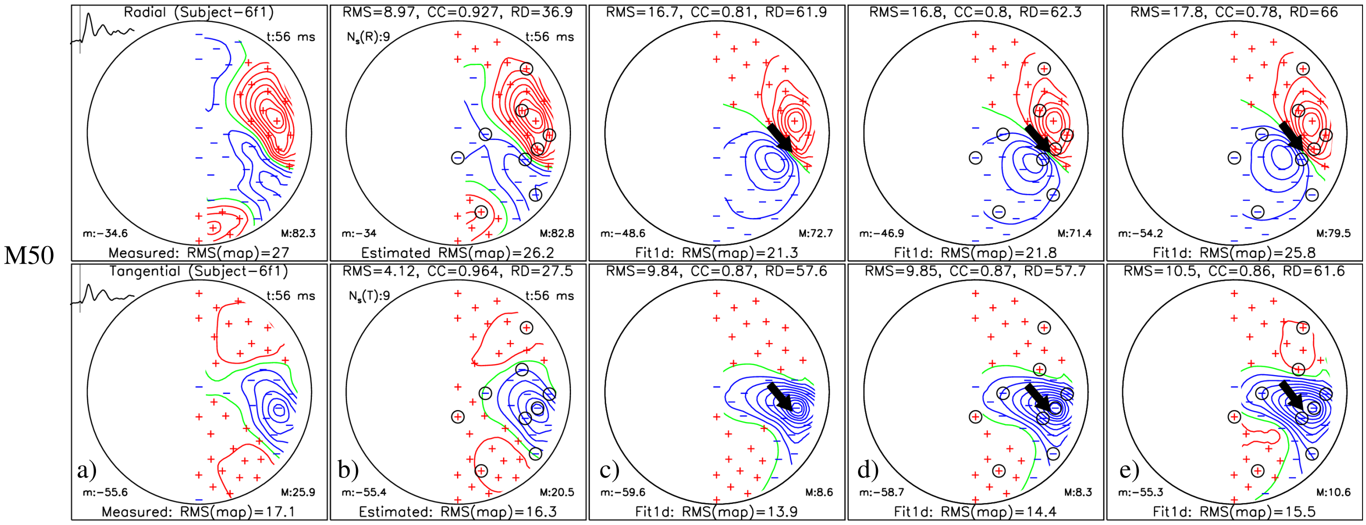

Fig. S.64: Subject-6f1: 18 selected channels using protocol **III** on right hemisphere only, fitting M50 with 1 dipole:
a) Measured data, b) Estimated data map,
c) measured map fit: $\vec{r}$=(59.9,-3.7,37.5), $\vec{p}$=(-0.6,0.8,1.1),
d) estimated map fit – source parameters: $\vec{r}$=(58.3,-4.3,37.7), $\vec{p}$=(-0.7,0.9,1.2),
reconstructed source errors: $\Delta\vec{r}$=(-1.5,-0.6,0.2), Δr=1.7, $\Delta\vec{p}$=(-0.1,0,0.1), Δp=0.2, $\Delta\varphi$=0.04,
e) selected chan. fit: $\vec{r}$=(55.1,-2.3,43.1), $\vec{p}$=(-0.9,1.3,1.2),
reconstructed source errors: $\Delta\vec{r}$=(-4.7,1.4,5.6), Δr=7.5, $\Delta\vec{p}$=(-0.3,0.4,0.2), Δp=0.6, $\Delta\varphi$=0.135.

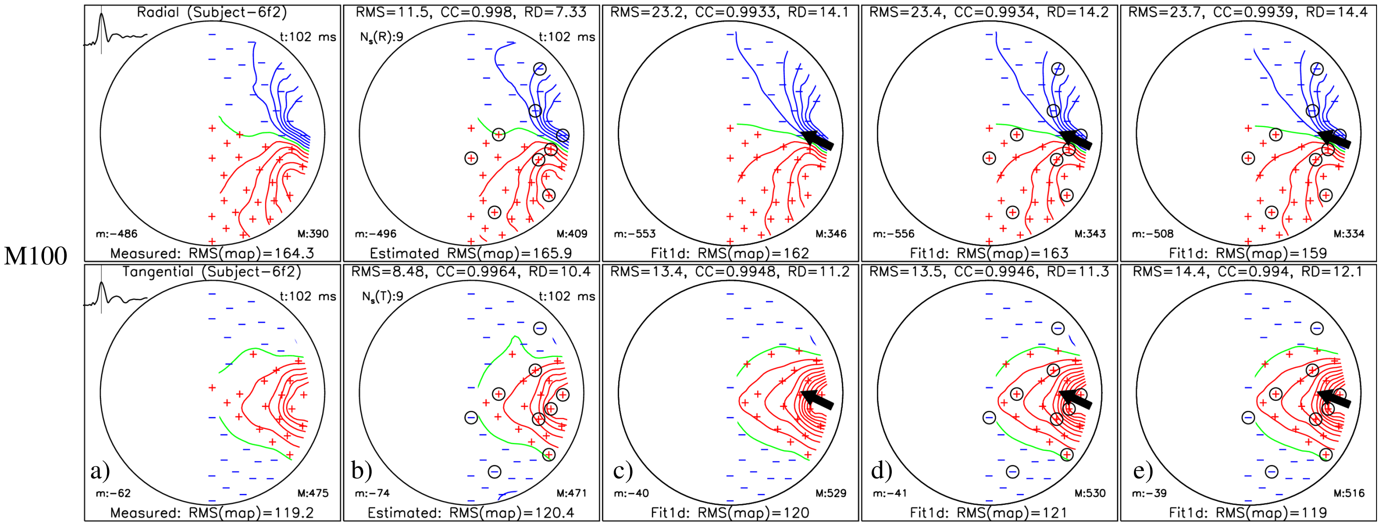

Fig. S.65: Subject-6f2: 18 selected channels using protocol **III** on right hemisphere only, fitting M100 with 1 dipole:
a) Measured data, b) Estimated data map,
c) measured map fit: $\vec{r}$=(53.6,-2.9,8.6), $\vec{p}$=(3.3,-4.4,-22.1),
d) estimated map fit – source parameters: $\vec{r}$=(53.8,-2.7,9.4), $\vec{p}$=(3.6,-4.3,-21.7),
reconstructed source errors: $\Delta\vec{r}$=(0.3,0.2,0.8), Δr=0.9, $\Delta\vec{p}$=(0.3,0.1,0.4), Δp=0.5, $\Delta\varphi$=0.015,
e) selected chan. fit: $\vec{r}$=(52.9,-2.4,9.7), $\vec{p}$=(3.9,-3.8,-22.1),
reconstructed source errors: $\Delta\vec{r}$=(-0.6,0.5,1.1), Δr=1.4, $\Delta\vec{p}$=(0.6,0.6,0), Δp=0.8, $\Delta\varphi$=0.037.

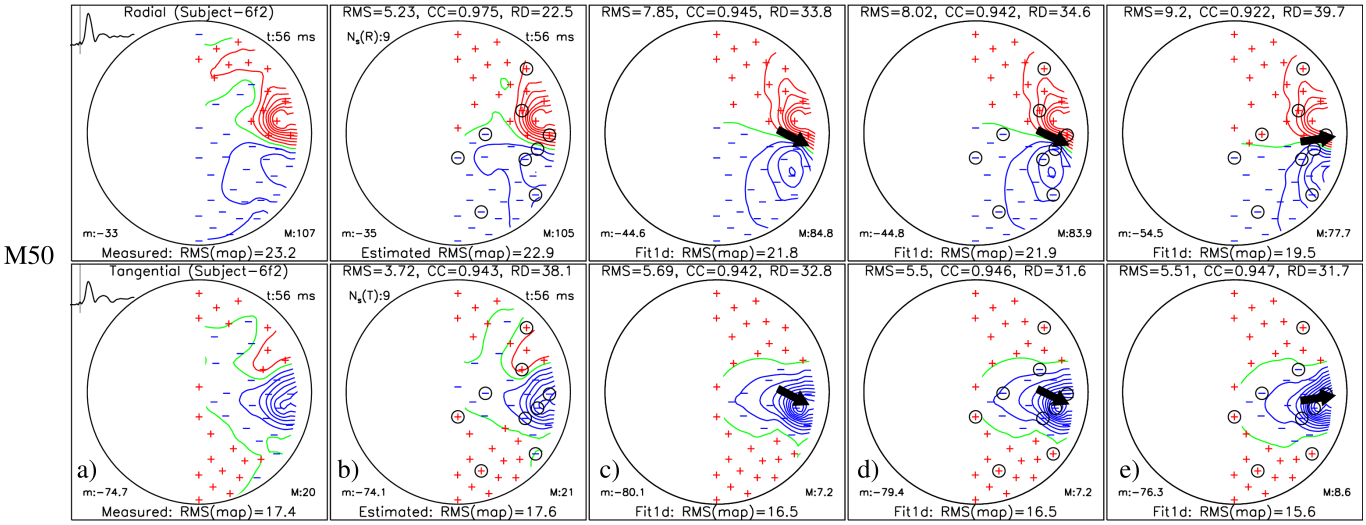

Fig. S.66: Subject-6f2: 18 selected channels using protocol **III** on right hemisphere only, fitting M50 with 1 dipole:
a) Measured data, b) Estimated data map,
c) measured map fit: $\vec{r}$=(63.6,-2.4,22.6), $\vec{p}$=(-0.5,0.3,1.4),
d) estimated map fit – source parameters: $\vec{r}$=(63.5,-2.3,21.5), $\vec{p}$=(-0.5,0.3,1.5),
reconstructed source errors: $\Delta\vec{r}$=(-0.1,0.1,-1.1), Δr=1.1, $\Delta\vec{p}$=(0,0,0), Δp=0, $\Delta\varphi$=0.021,
e) selected chan. fit: $\vec{r}$=(66.5,-3.4,14.3), $\vec{p}$=(-0.3,-0.1,1.3),
reconstructed source errors: $\Delta\vec{r}$=(3,-1,-8.3), Δr=8.9, $\Delta\vec{p}$=(0.2,-0.4,-0.2), Δp=0.5, $\Delta\varphi$=0.324.

M100

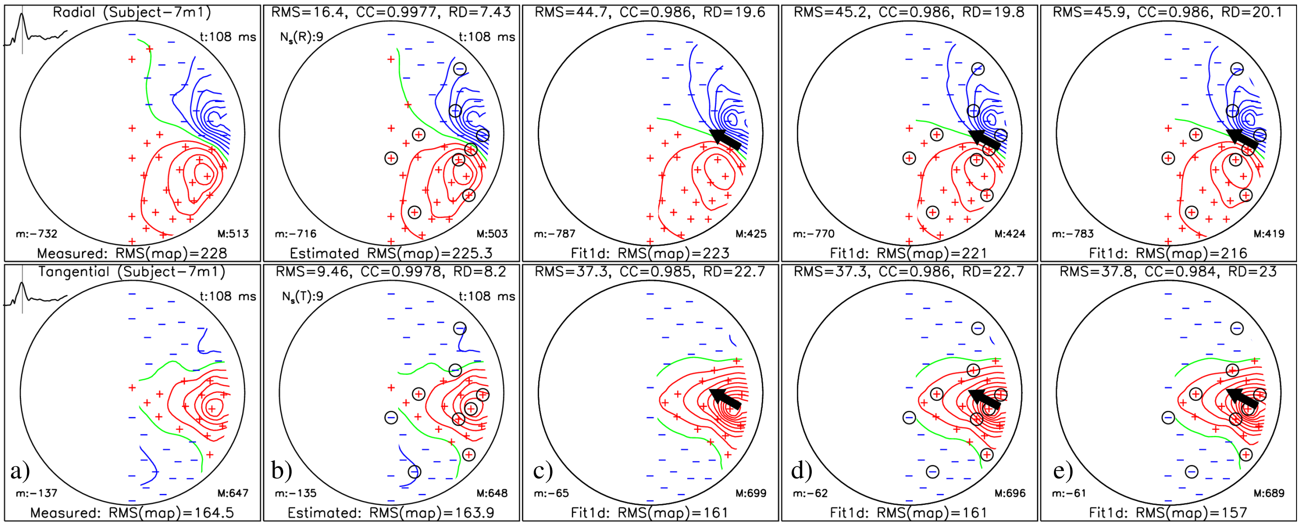

Fig. S.67: Subject-7m1: 18 selected channels using protocol **III** on right hemisphere only, fitting M100 with 1 dipole:
a) Measured data, b) Estimated data map,
c) measured map fit: $\vec{r}$=(57.7,-2.8,23.3), $\vec{p}$=(7.3,-6.3,-18.9),
d) estimated map fit – source parameters: $\vec{r}$=(57.5,-3.1,22.8), $\vec{p}$=(7.3,-5.8,-19.3),
reconstructed source errors: $\Delta\vec{r}$=(-0.3,-0.3,-0.5), Δr=0.6, $\Delta\vec{p}$=(0,0.5,-0.4), Δp=0.6, $\Delta\varphi$=0.028,
e) selected chan. fit: $\vec{r}$=(58.4,-2.5,24.8), $\vec{p}$=(7.1,-5.6,-17.4),
reconstructed source errors: $\Delta\vec{r}$=(0.7,0.3,1.5), Δr=1.7, $\Delta\vec{p}$=(-0.2,0.7,1.6), Δp=1.8, $\Delta\varphi$=0.022.

M50

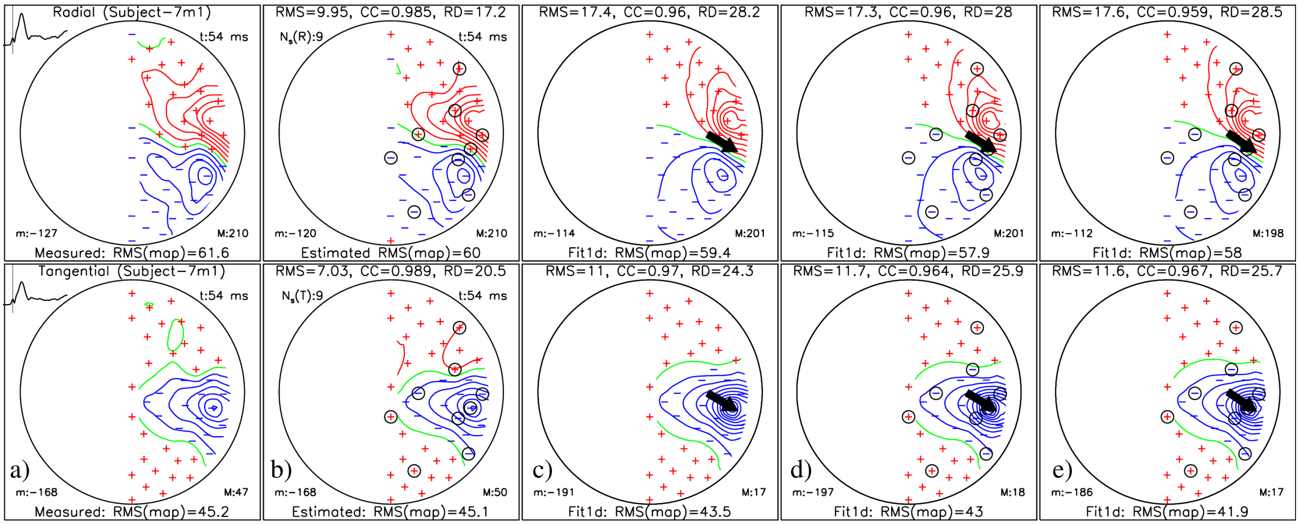

Fig. S.68: Subject-7m1: 9 selected sites using protocol **III** on right hemisphere only, fitting M50 with 1 dipole:
a) Measured data, b) Estimated data map,
c) measured map fit: $\vec{r}$=(57.4,-6.3,24.5), $\vec{p}$=(-2,1.3,5.1),
d) estimated map fit – source parameters: $\vec{r}$=(59.1,-6.3,25), $\vec{p}$=(-1.8,1.2,4.6),
reconstructed source errors: $\Delta\vec{r}$=(1.6,0,0.5), Δr=1.7, $\Delta\vec{p}$=(0.2,-0.1,-0.5), Δp=0.6, $\Delta\varphi$=0.011,
e) selected chan. fit: $\vec{r}$=(57.7,-6,22.4), $\vec{p}$=(-1.8,1.6,5),
reconstructed source errors: $\Delta\vec{r}$=(0.3,0.3,-2.1), Δr=2.1, $\Delta\vec{p}$=(0.2,0.2,-0.1), Δp=0.3, $\Delta\varphi$=0.06.

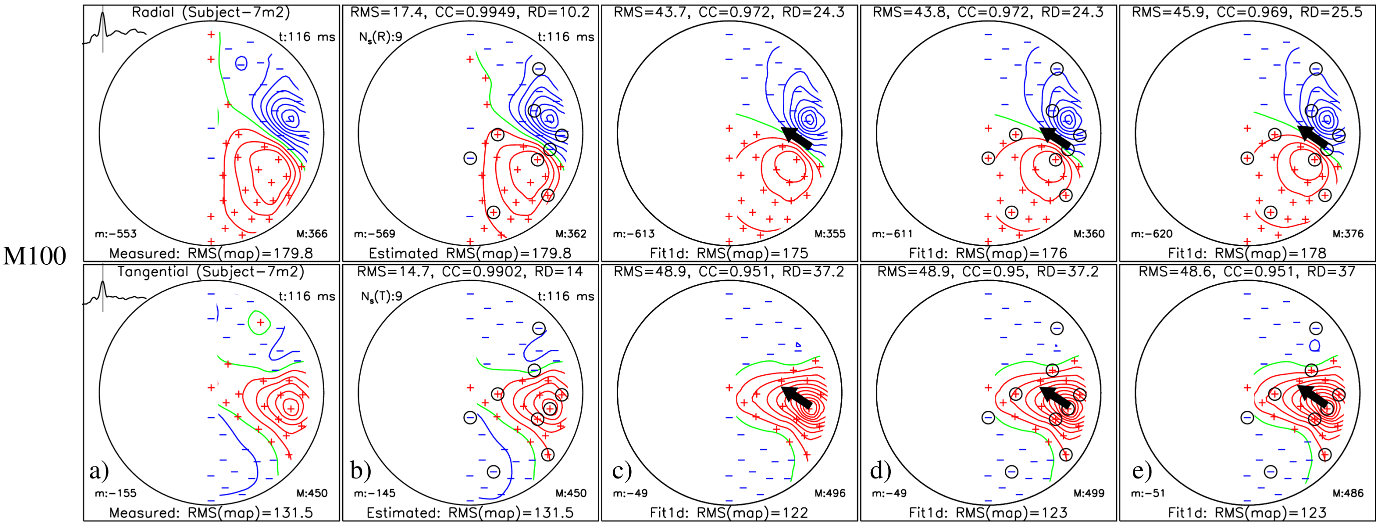

Fig. S.69: Subject-7m2: 18 selected channels using protocol **III** on right hemisphere only, fitting M100 with 1 dipole:
a) Measured data, b) Estimated data map,
c) measured map fit: $\vec{r}$=(56.2,-1.8,32), $\vec{p}$=(7.1,-6.1,-12.9),
d) estimated map fit – source parameters: $\vec{r}$=(56.2,-1.8,32.5), $\vec{p}$=(7.2,-5.9,-12.8),
reconstructed source errors: $\Delta\vec{r}$=(0.1,-0.1,0.5), Δr=0.5, $\Delta\vec{p}$=(0.1,0.2,0.1), Δp=0.2, $\Delta\varphi$=0.01,
e) selected chan. fit: $\vec{r}$=(56.3,-0.8,34.6), $\vec{p}$=(7.4,-6.4,-12.1),
reconstructed source errors: $\Delta\vec{r}$=(0.1,1,2.6), Δr=2.8, $\Delta\vec{p}$=(0.2,-0.3,0.8), Δp=0.8, $\Delta\varphi$=0.048.

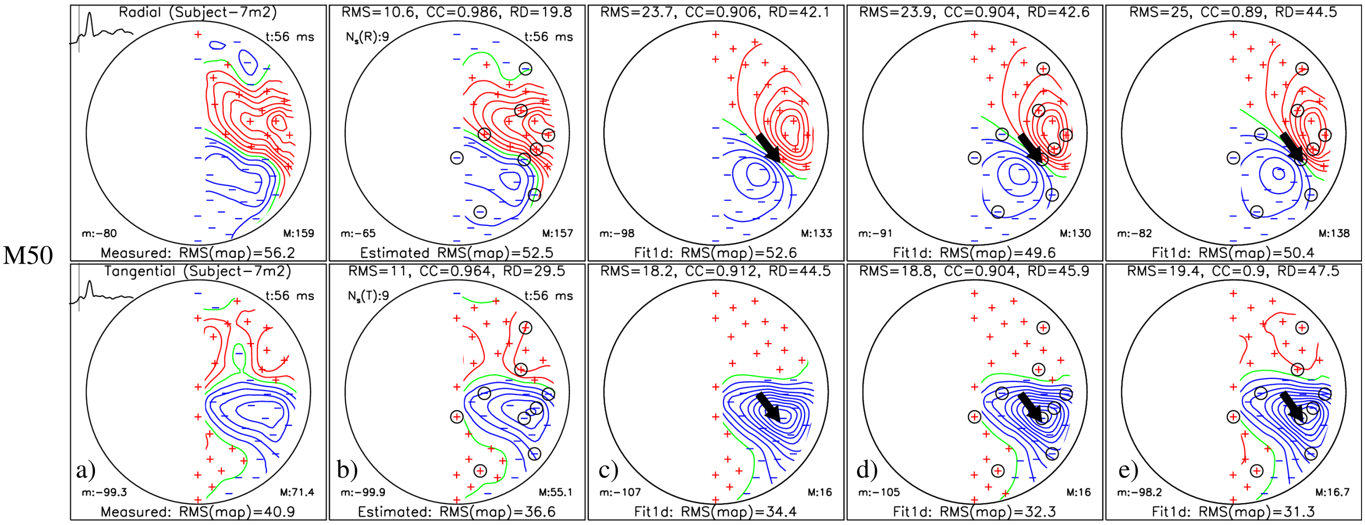

Fig. S.70: Subject-7m2: 18 selected channels using protocol **III** on right hemisphere only, fitting M50 with 1 dipole:
a) Measured data, b) Estimated data map,
c) measured map fit: $\vec{r}$=(47.5,-12.2,41.9), $\vec{p}$=(-2.8,2.7,3.9),
d) estimated map fit – source parameters: $\vec{r}$=(50.4,-12.3,40), $\vec{p}$=(-2.2,2.3,3.5),
reconstructed source errors: $\Delta\vec{r}$=(2.9,-0.1,-1.9), Δr=3.4, $\Delta\vec{p}$=(0.5,-0.3,-0.4), Δp=0.7, $\Delta\varphi$=0.045,
e) selected chan. fit: $\vec{r}$=(49.5,-10.9,35.9), $\vec{p}$=(-2.2,2.9,3.9),
reconstructed source errors: $\Delta\vec{r}$=(2,1.3,-6), Δr=6.5, $\Delta\vec{p}$=(0.5,0.3,0), Δp=0.6, $\Delta\varphi$=0.112.

M100

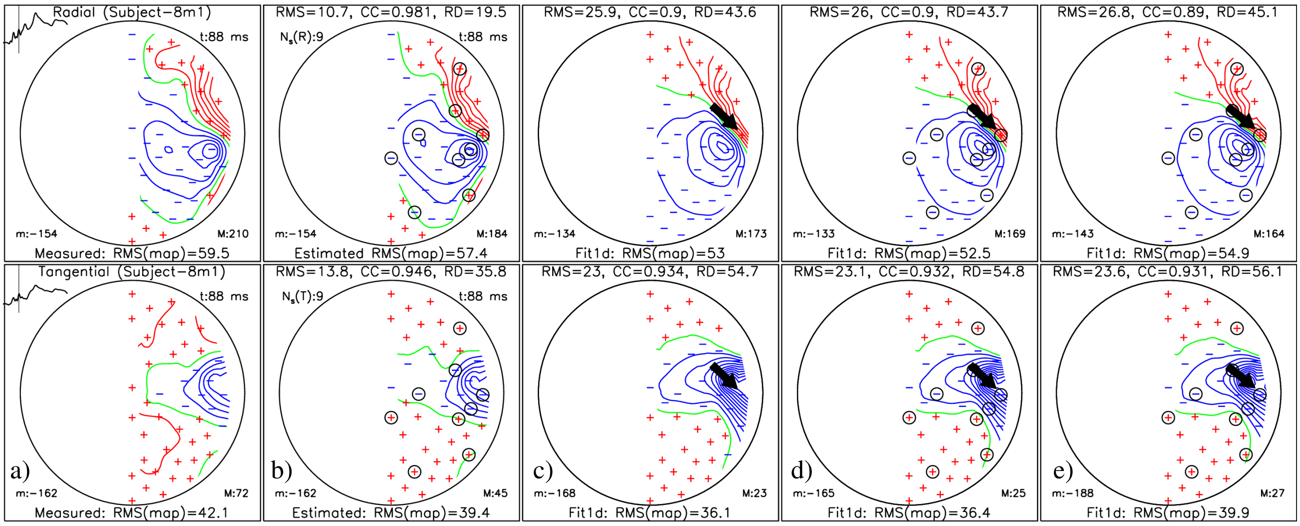

Fig. S.71: Subject-8m1: 18 selected channels using protocol **III** on right hemisphere only, fitting M100 with 1 dipole:
a) Measured data, b) Estimated data map,
c) measured map fit: $\vec{r}$=(57.2,13.2,21.1), $\vec{p}$=(-2.2,3.4,3.8),
d) estimated map fit – source parameters: $\vec{r}$=(57.7,13.5,22), $\vec{p}$=(-2.1,3.1,3.7),
reconstructed source errors: $\Delta\vec{r}$=(0.6,0.3,0.9), Δr=1.1, $\Delta\vec{p}$=(0.1,-0.3,-0.1), Δp=0.3, $\Delta\varphi$=0.027,
e) selected chan. fit: $\vec{r}$=(58.6,13.6,23.5), $\vec{p}$=(-2.1,2.7,3.8),
reconstructed source errors: $\Delta\vec{r}$=(1.4,0.4,2.4), Δr=2.8, $\Delta\vec{p}$=(0.1,-0.8,0), Δp=0.8, $\Delta\varphi$=0.11.

M50

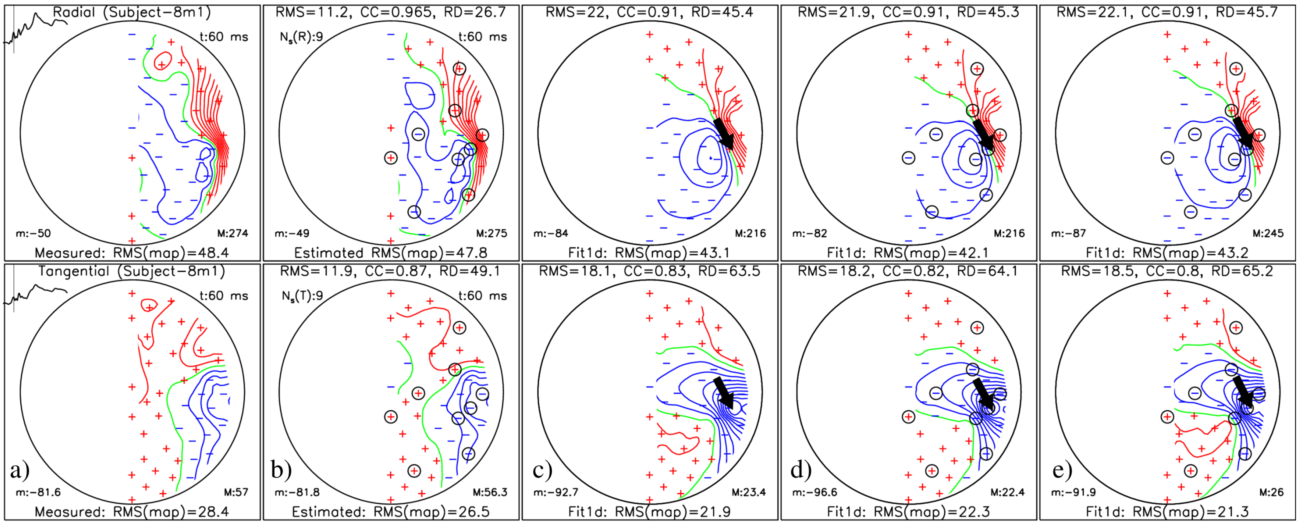

Fig. S.72: Subject-8m1: 18 selected channels using protocol **III** on right hemisphere only, fitting M50 with 1 dipole:
a) Measured data, b) Estimated data map,
c) measured map fit: $\vec{r}$=(56.7,0,22.4), $\vec{p}$=(-1,3.7,2.6),
d) estimated map fit – source parameters: $\vec{r}$=(57.8,-0.8,21.9), $\vec{p}$=(-0.9,3.3,2.6),
reconstructed source errors: $\Delta\vec{r}$=(1.1,-0.8,-0.5), Δr=1.5, $\Delta\vec{p}$=(0.1,-0.4,0), Δp=0.4, $\Delta\varphi$=0.05,
e) selected chan. fit: $\vec{r}$=(59.8,0.8,21.7), $\vec{p}$=(-0.8,3.2,2.2),
reconstructed source errors: $\Delta\vec{r}$=(3.1,0.8,-0.7), Δr=3.3, $\Delta\vec{p}$=(0.2,-0.4,-0.4), Δp=0.6, $\Delta\varphi$=0.032.

M100

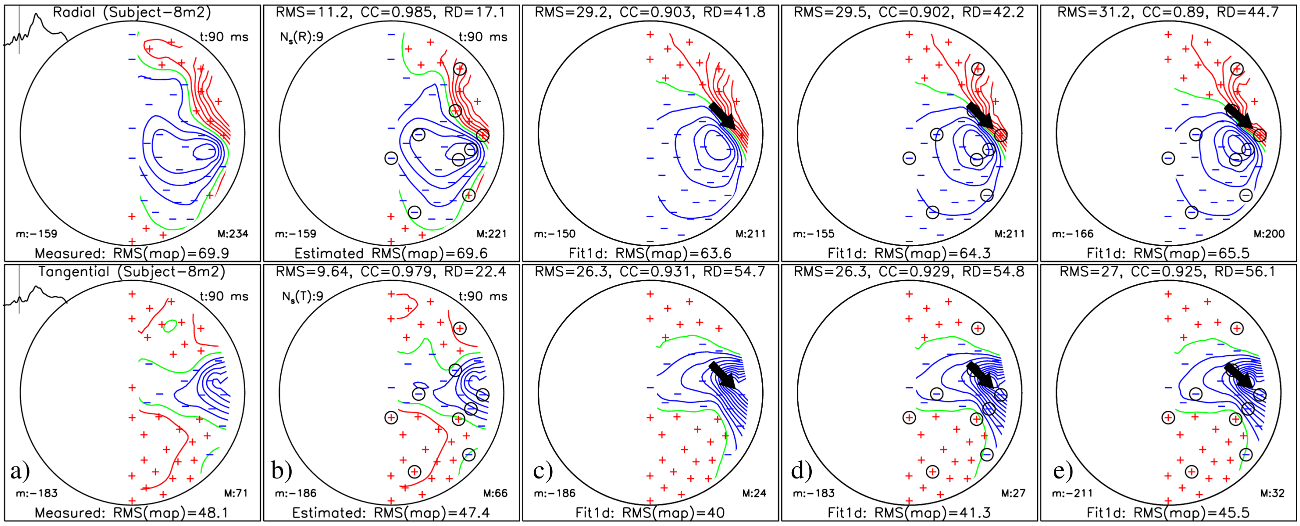

Fig. S.73: Subject-8m2: 18 selected channels using protocol **III** on right hemisphere only, fitting M100 with 1 dipole:
a) Measured data, b) Estimated data map,
c) measured map fit: $\vec{r}$=(54.9,13.4,21.9), $\vec{p}$=(-3.1,5,4.6),
d) estimated map fit – source parameters: $\vec{r}$=(55.7,14,23.5), $\vec{p}$=(-3,4.6,4.4),
reconstructed source errors: $\Delta\vec{r}$=(0.8,0.6,1.5), Δr=1.8, $\Delta\vec{p}$=(0.1,-0.4,-0.2), Δp=0.5, $\Delta\varphi$=0.023,
e) selected chan. fit: $\vec{r}$=(57.6,14.1,25.2), $\vec{p}$=(-2.8,3.6,4.3),
reconstructed source errors: $\Delta\vec{r}$=(2.7,0.7,3.2), Δr=4.3, $\Delta\vec{p}$=(0.3,-1.4,-0.3), Δp=1.5, $\Delta\varphi$=0.127.

M50

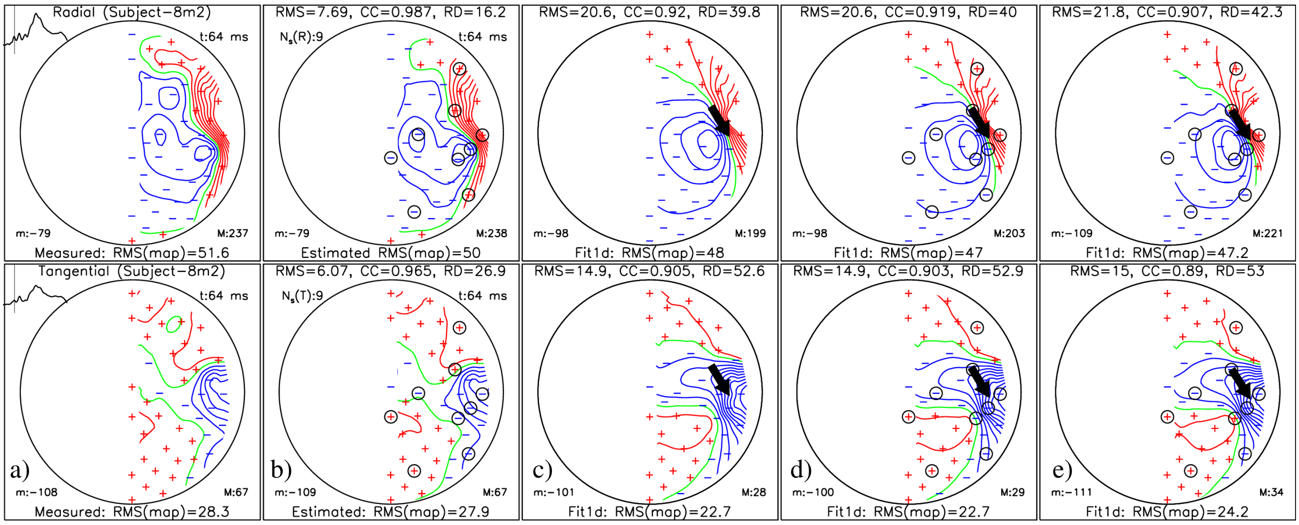

Fig. S.74: Subject-8m2: 18 selected channels using protocol **III** on right hemisphere only, fitting M50 with 1 dipole:
a) Measured data, b) Estimated data map,
c) measured map fit: $\vec{r}$=(53.1,9.8,24.8), $\vec{p}$=(-2,5,2.3),
d) estimated map fit – source parameters: $\vec{r}$=(54.8,8.5,24.5), $\vec{p}$=(-1.7,4.5,2.3),
reconstructed source errors: $\Delta\vec{r}$=(1.7,-1.3,-0.3), Δr=2.1, $\Delta\vec{p}$=(0.3,-0.5,0), Δp=0.6, $\Delta\varphi$=0.041,
e) selected chan. fit: $\vec{r}$=(59.5,7.6,24.3), $\vec{p}$=(-1.3,3.4,2.1),
reconstructed source errors: $\Delta\vec{r}$=(6.4,-2.3,-0.5), Δr=6.8, $\Delta\vec{p}$=(0.7,-1.6,-0.2), Δp=1.8, $\Delta\varphi$=0.123.

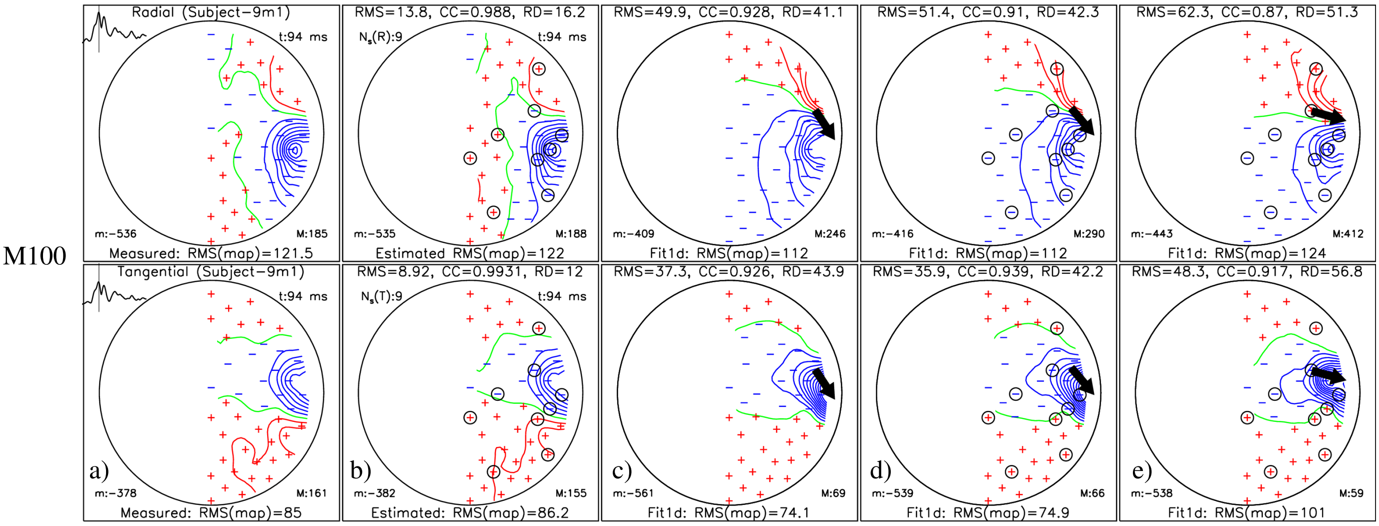

Fig. S.75: Subject-9m1: 18 selected channels using protocol **III** on right hemisphere only, fitting M100 with 1 dipole:
a) Measured data, b) Estimated data map,
c) measured map fit: $\vec{r}$=(64.9,6.8,-2.4), $\vec{p}$=(-0.5,7.1,7.2),
d) estimated map fit – source parameters: $\vec{r}$=(63.8,8.9,-1.1), $\vec{p}$=(-0.7,6.2,8.1),
reconstructed source errors: $\Delta\vec{r}$=(-1.2,2.2,1.3), Δr=2.8, $\Delta\vec{p}$=(-0.2,-0.9,0.9), Δp=1.3, $\Delta\varphi$=0.128,
e) selected chan. fit: $\vec{r}$=(65,14.9,14.5), $\vec{p}$=(-2.2,1.6,8.1),
reconstructed source errors: $\Delta\vec{r}$=(0.1,8.2,16.9), Δr=18.7, $\Delta\vec{p}$=(-1.7,-5.4,0.9), Δp=5.8, $\Delta\varphi$=0.605.

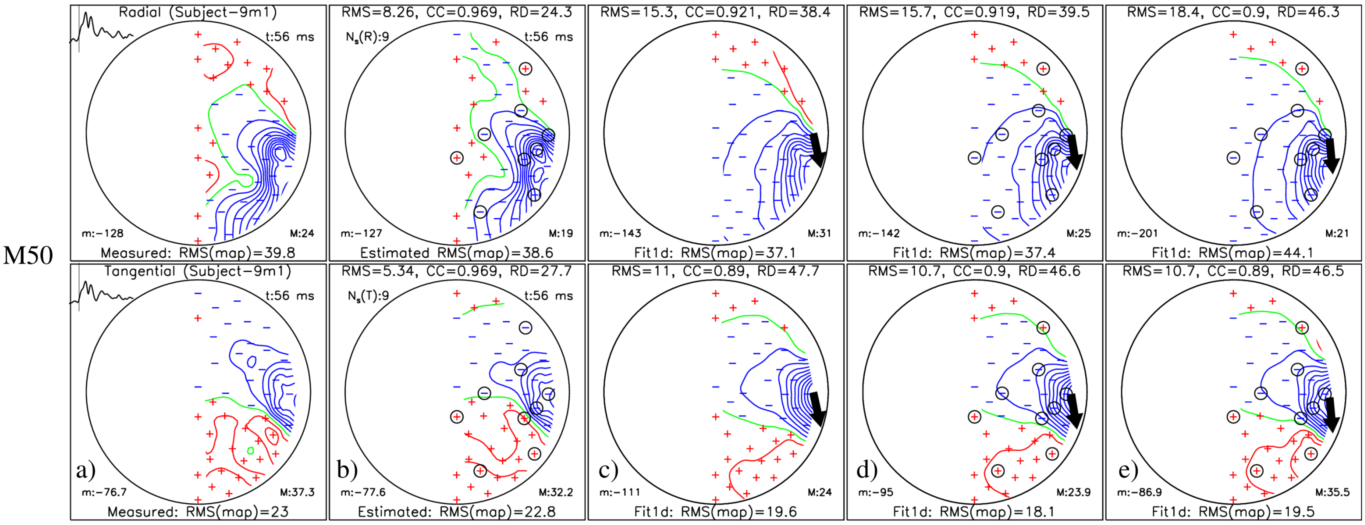

Fig. S.76: Subject-9m1: 18 selected channels using protocol **III** on right hemisphere only, fitting M50 with 1 dipole:
a) Measured data, b) Estimated data map,
c) measured map fit: $\vec{r}$=(61.4,-9.5,-11.3), $\vec{p}$=(1,3.5,2.6),
d) estimated map fit – source parameters: $\vec{r}$=(63.2,-11.1,-9.3), $\vec{p}$=(0.9,3.4,2),
reconstructed source errors: $\Delta\vec{r}$=(1.7,-1.6,1.9), Δr=3, $\Delta\vec{p}$=(-0.1,-0.1,-0.6), Δp=0.6, $\Delta\varphi$=0.102,
e) selected chan. fit: $\vec{r}$=(67.9,-14.7,-8.6), $\vec{p}$=(0.9,3.2,1.5),
reconstructed source errors: $\Delta\vec{r}$=(6.4,-5.1,2.7), Δr=8.7, $\Delta\vec{p}$=(-0.1,-0.2,-1.1), Δp=1.1, $\Delta\varphi$=0.2.

M100

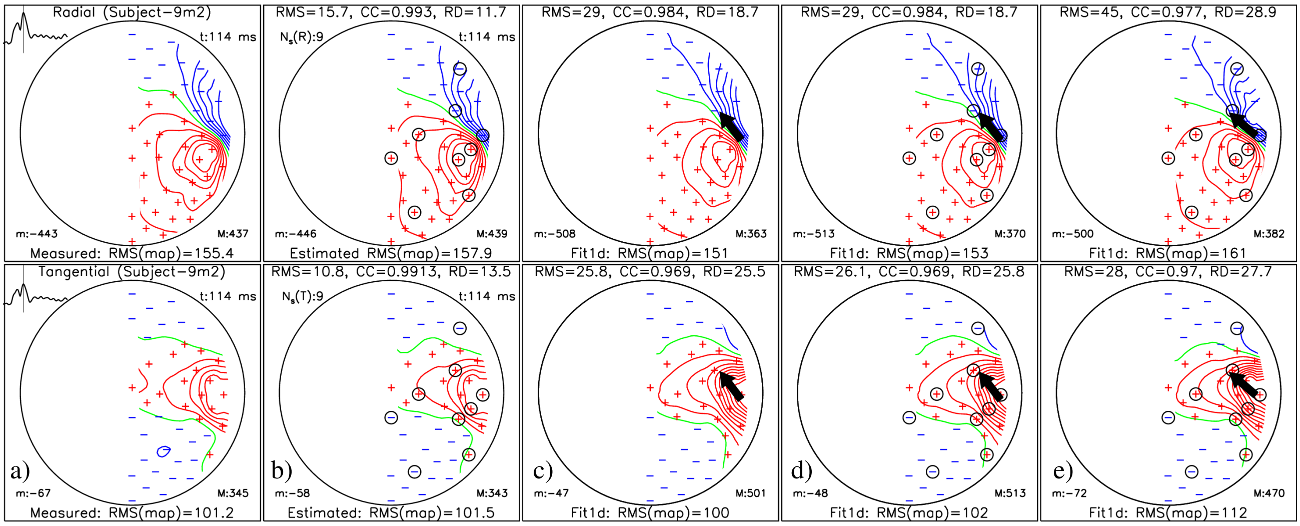

Fig. S.77: Subject-9m2: 18 selected channels using protocol **III** on right hemisphere only, fitting M100 with 1 dipole:
a) Measured data, b) Estimated data map,
c) measured map fit: $\vec{r}$=(54,5.9,14.9), $\vec{p}$=(5.6,-11.9,-15.5),
d) estimated map fit – source parameters: $\vec{r}$=(54.2,5.6,14.6), $\vec{p}$=(5.5,-11.8,-15.8),
reconstructed source errors: $\Delta\vec{r}$=(0.2,-0.3,-0.3), Δr=0.5, $\Delta\vec{p}$=(-0.1,0.2,-0.3), Δp=0.3, $\Delta\varphi$=0.017,
e) selected chan. fit: $\vec{r}$=(57.3,8.1,22.5), $\vec{p}$=(6.1,-7.7,-12.7),
reconstructed source errors: $\Delta\vec{r}$=(3.3,2.2,7.6), Δr=8.6, $\Delta\vec{p}$=(0.5,4.3,2.8), Δp=5.1, $\Delta\varphi$=0.153.

M50

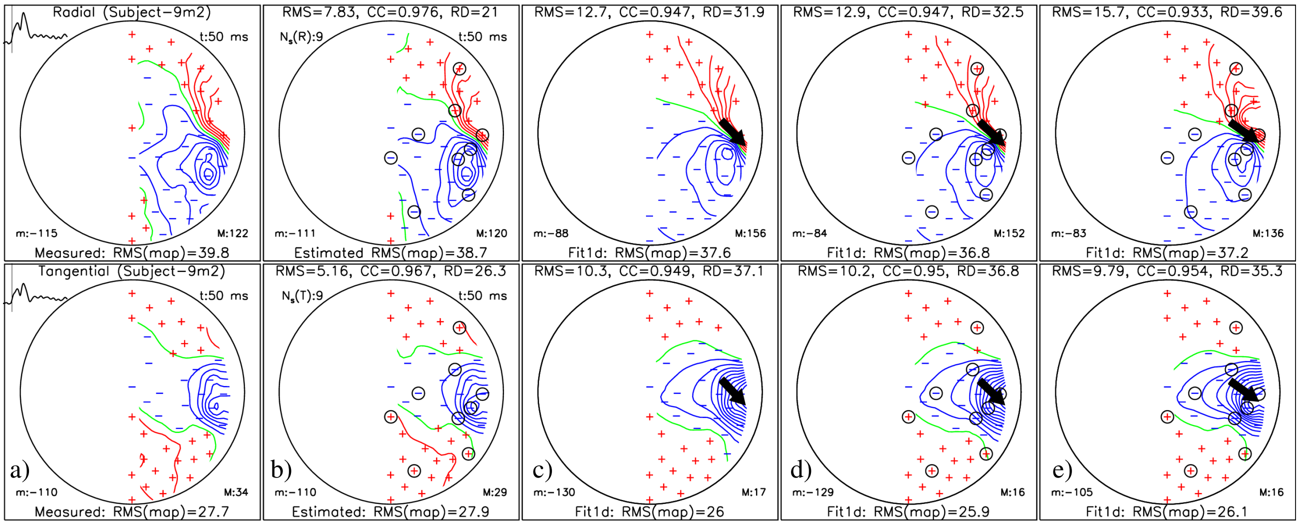

Fig. S.78: Subject-9m2: 18 selected channels using protocol **III** on right hemisphere only, fitting M50 with 1 dipole:
a) Measured data, b) Estimated data map,
c) measured map fit: $\vec{r}$=(57.3,1.1,12.5), $\vec{p}$=(-0.8,2.2,3.7),
d) estimated map fit – source parameters: $\vec{r}$=(57.7,0.7,12.6), $\vec{p}$=(-0.8,1.9,3.6),
reconstructed source errors: $\Delta\vec{r}$=(0.4,-0.4,0.2), Δr=0.5, $\Delta\vec{p}$=(0,-0.3,-0.1), Δp=0.3, $\Delta\varphi$=0.055,
e) selected chan. fit: $\vec{r}$=(60.3,2,21.1), $\vec{p}$=(-1,1.1,2.8),
reconstructed source errors: $\Delta\vec{r}$=(3,0.9,8.7), Δr=9.2, $\Delta\vec{p}$=(-0.2,-1.1,-0.9), Δp=1.4, $\Delta\varphi$=0.198.
